# Utx Is Required for Proper Induction of Ectoderm and Mesoderm during Differentiation of Embryonic Stem Cells

**DOI:** 10.1371/journal.pone.0060020

**Published:** 2013-04-03

**Authors:** Cristina Morales Torres, Anne Laugesen, Kristian Helin

**Affiliations:** 1 Biotech Research and Innovation Centre (BRIC), University of Copenhagen, Copenhagen, Denmark; 2 Centre for Epigenetics, University of Copenhagen, Copenhagen, Denmark; 3 The Danish Stem Cell Center, University of Copenhagen, Copenhagen, Denmark; National University of Singapore, Singapore

## Abstract

Embryonic development requires chromatin remodeling for dynamic regulation of gene expression patterns to ensure silencing of pluripotent transcription factors and activation of developmental regulators. Demethylation of H3K27me3 by the histone demethylases *Utx* and *Jmjd3* is important for the activation of lineage choice genes in response to developmental signals. To further understand the function of Utx in pluripotency and differentiation we generated *Utx* knockout embryonic stem cells (ESCs). Here we show that Utx is not required for the proliferation of ESCs, however, Utx contributes to the establishment of ectoderm and mesoderm in vitro. Interestingly, this contribution is independent of the catalytic activity of Utx. Furthermore, we provide data showing that the Utx homologue, Uty, which is devoid of detectable demethylase activity, and Jmjd3 partly compensate for the loss of Utx. Taken together our results show that Utx is required for proper formation of ectoderm and mesoderm in vitro, and that Utx, similar to its *C.elegans* homologue, has demethylase dependent and independent functions.

## Introduction

Mouse embryonic stem cells (ESCs) are pluripotent cells derived from the inner cell mass of a blastocyst that can be propagated as a cell line in tissue culture. They have the capability of self-renewal and are able to differentiate into all three germ layers (ectoderm, mesoderm and endoderm) that will later differentiate into the distinct cell types present in adult mice. During this process changes in chromatin structure is accompanied by the de-repression of lineage-specific genes and repression of pluripotent transcription factors, such as Oct4 and Nanog [1], [2].

Several mechanisms are involved in the regulation of chromatin architecture in ESCs, including post-translational modifications of histone tails like acetylation, methylation, phosphorylation, and ubiquitylation [3]–[6]. The combination of these modifications regulates chromatin structure and functions mainly by modulating histone-DNA interactions and the binding of effector proteins that recognize specific modified/unmodified states of the histones translating this information into different biological outcomes [5], [7].

Lysine residues of histone tails can be mono-, di- or tri-methylated and the degree of methylation as well as the specific residue methylated influences which proteins can bind to chromatin and modify the chromatin state [5]. For instance, while methylation of histone H3 Lysine 27 (H3K27me) is associated with transcriptional repression [8]–[11], methylation of H3 Lysine 4 (H3K4me) is found at sites of active transcription [12]–[15].

The Polycomb repressive complex 2 (PRC2), and more specifically its catalytic subunit Ezh2, is responsible for di- and tri-methylation of H3K27. Whereas the PRC2 core components Ezh2, Suz12 and Eed are essential for early embryonic development, they are not required for ESC proliferation [16]–[19]. However, consistent with their essential role in embryonic development, the three PRC2 subunits are required for ESC differentiation [20], [21]. PRC2 controls the expression of a number of genes required for lineage determination. Many of these genes are typically associated with “bivalent” chromatin marks containing trimethylated H3K4 and H3K27. It has been hypothesized that through these bivalent marks, differentiation genes controlled by PRC2 may be poised for activation upon removal of their repressive epigenetic marks [11], [22]–[24].

Utx (Kdm6a) and Jmjd3 (Kdm6b) catalyze the demethylation of H3K27me3 and H3K27me2. *Utx* is localized on the X chromosome, is ubiquitiously expressed and escapes X-chromosome inactivation [25]. *Uty*, a homologue of *Utx*, is present on the Y chromosome, and its protein product Uty does not appear to be catalytically active [26]–[30].

UTX is required for the activation of homeotic genes [27], [31], [32] and is part of a subset of the mixed lineage leukemia (Mll) protein complexes. The Mll proteins are members of the trithorax group of proteins that catalyze the trimethylation of H3K4 and are involved in gene activation. The Utx-Mll complexes contain either Mll3 or Mll4, together with Pa1, Ptip and NcoA6 as specific subunits of these complexes [31]–[34].

Utx homologues in zebrafish (Utx-1 Utx-2) and *C. elegans* (Utx-1) have important roles in normal development [27], [35]. In flies, Utx co-localizes with the elongating form of RNAPII, suggesting a role for H3K27 demethylation in transcriptional elongation [36]. Utx is highly expressed in mouse embryos, in particular in developing heart, neural tube, neural crest cells, somites, otic placode, limb buds, brachial arches, isthmus, cortex and eyes [37]. *Utx* knockout mice display abnormal or truncated posterior bodies, and defects in cardiac development and neural tube closure [29], [30], [37], [38]. Whereas knockout females show embryonic lethality at 10.5 dpc, knockout males display a partial embryonic lethality phenotype with increased tumor formation during adulthood [29], [30], [37], [38]. These results suggest that the catalytically inactive Uty can compensate for some of the functions of Utx.

A demethylase independent role of UTX has indeed been described in *C. elegans* [35], and while this manuscript was in preparation it has been shown that Utx is required for induction [39], and for mesoderm differentiation of ESCs independent of its H3K27 demethylase activity [30], [38]. In this study, we have addressed the role of Utx in ESC differentiation. Consistent with the recent published results we show that Utx is required for mesodermal differentiation of ESCs. In addition we show that Utx also has a role in the regulation of ectoderm differentiation, and that Uty and Jmjd3 play partly redundant roles in this regulation.

## Materials and Methods

### ESCs Culture and Differentiation

ESCs were grown on 0.2% gelatin (Sigma) coated tissue culture plates (Nunc) and cultured in 2i medium [40] containing: DMEM/F12 (Gibco; 31331) and Neurobasal (Gibco; 12348) 1∶1, N2 supplement (Invitrogen; 17502048), B27 supplement (Invitrogen; 17504044), 1 mM glutamine (Invitrogen), Pen-Strep (Gibco; 15140), Sodium Pyruvate 100 mM (Gibco; 11360) 2-mercaptoethanol 50 mM (Gibco; 31350), nonessential amino acids (Gibco; 11140), LIF (ESGRO), GSK3 (3 mM) and MEK1 (2 mM) inhibitors. When ESCs were differentiated a 2i-modified media was used as described above, but without LIF and inhibitors. To induce ESC monolayer differentiation, 7.5×10^5^ cells per 10-cm dish were plated and induced to differentiate 16 h after by adding modified 2i medium with 1 µM All Trans Retinoic Acid (RA) (Sigma) for 72 h, taking samples every 24 h. EBs were generated using the ‘hanging drop’ method. Drops of 1000 cells in 20 µl 2i modified medium were placed in the lid of tissue culture dishes. Aggregates were allowed to form for 48 h, harvested and plated into bacterial dishes in the same medium. Samples were taken before and after 3 d, 6 d and 9 d of differentiation.

Homogeneous cardiomyocytes were generated as previously described [41]. Briefly, EBs were generated as hanging drops and were plated on bacterial dishes 48 h after aggregates were formed. After 7 days, EBs were plated individually into tissue culture dishes coated with 0.1% gelatin. Spontaneous beating areas could be detected as early as day 8.

### shRNA-mediated Knockdown

All knockdown experiments were done using shRNAs. Viral transductions were performed using pLKO vectors from Sigma (NM_009484.1-809s1c1 (Uty-shRNA1), NM_009484.1-1113s1c1 (Uty-shRNA2), NM-009484.1-808s1c1 (Uty-shRNA3), NM_001017426.1-2839s1c1 (Jmjd3-shRNA1), NM_001017426.1-5159s1c1 (Jmjd3-shRNA2), NM_001017426.1-3013s1c1 (Jmjd3-shRNA3) and SHC201 (*Scr-*shRNA). Lentiviral particles were produced in 293FT cells. ESCs were transduced with lentiviral particles for 16 h, and selected with 2 µg/ml Puromycin (Invitrogen) 48 h after transduction.

### Generation of *Utx* Conditional Construct and Knockout ESCs

We designed a conditional targeting vector that after deletion of exon 3 produce a frame shift and introduce a translational stop codon. The Utx conditional construct was generated using the methodology described by Zhang et al. [42] **.** Briefly, the conditional allele had been generated by an in vivo λKO-2 based 129/SvEvBrd mouse genomic library screening, in a two step homologous recombination process that introduces two loxP recombination sites flanking exon 3 within the gene, together with a kanamycin/neomycin resistance cassette for positive selection. The construct also contains a Thymidine Kinase (TK) gene. Targeted ES cells were generated at the University of California, Davis, Mouse Biology Program (MBP) by electroporation of the construct into R1 (129/Sv) mouse ES-cells [43]. Targeted cells were selected with 200 µg/ml of G418 and 2 µM of Ganciclovir (positive and negative selection) after 24 h and 48 h, respectively. Screening of targeted ES-clones was performed via Loss-of-Allele (LOA) assay and vector copy number analysis. Homologous recombination as well as distal loxP site integrity was confirmed by long-range PCR. Positive clones carrying the integrated targeting vector were subject to karyotype analysis and one of them (P1-E04) selected to proceed with FLP expressing vector electroporation to delete the Kn/Neo resistance cassette, thereby generating floxed clones. Three floxed confirmed clones (D02, D05, D06) were karyotyped and two (D02, D05) were transfected with a PGK-Cre-GFP plasmid and GFP expressing cells were FACS sorted. GFP positive cells were cloned by limited dilution and two clones for each previous floxed clone (D02: KO1, KO2 and D05: KO3, KO4) were genotyped for exon 3 deletion. For genotyping of Utx knockout ESCs, 5′-TTGATTTGGAAATAGGTTTGATTG-3′ forward, 5′-CCCAAAACTGGCAGGATATG-3′ reverse primers were used to discriminate wild type (1030 bp), floxed (1113 bp) and deleted (361 bp) alleles.

### Complementation of *Utx* Knockout ESCs with BACs Expressing Wild Type and Catalytic Inactive Mutant Utx

Briefly, using the mouse BAC RP23-214H5 that covers the mouse *Utx* gene locus, we C-terminally tagged the *Utx* gene with a 2xTy1-PreS-lox5171-mVenus-lox5171-Biotin-rox-T2A-gb3-Blasticidin/Kanamycin-rox-3xFlag cassette in a two-step homologous recombination process in *E. coli* [44]. For the catalytic inactive mutant we first mutated, in the BAC (using counter-selection BAC Modification kit from Gene Bridges), two of the known critical amino acids (H1146>A and E1148>A) of the JmjC domain responsible for iron binding [32].

Utx tagged BACs (wild type: +Wt, and catalytic inactive mutant: +Mut) were electroporated, using Amaxa - nucleofector II (Lonza) into *Utx* knockout clone 2 (KO2) and selected with Blasticidin (Sigma) 4 µg/ml for 10 days. The expression of Utx was tested in the selected clones by western blotting using an antibody to Utx [31] and Ty1 antibody (Diagenode, MAb-054-050).

### Antibodies

Utx polyclonal antibody was generated by immunizing rabbits with bacterially expressed affinity-purified GST–UTX (amino acids 453–753). The polyclonal antibody was affinity-purified using GST–UTX-coupled Sepharose [31]. The JMJD3 antisera were generated in rabbits, using affinity-purified GST-JMJD3 (amino acids 798–1095). The polyclonal antibody was affinity-purified on GST-JMJD3. The specificity of the antibodies was confirmed by immunoblotting and immunoprecipitation [45]. The other antibodies used in this study were: anti-H3K4me3 (Cell Signaling, C42D8), anti-H3K27me3 (Cell Signaling, D18C8), anti-H3 (Abcam, 1791), anti-Vinculin (Sigma, V9131), anti-Nanog (Abcam, 80892), anti-Oct4 (Abcam, 19857), anti-BrdU (Becton Dickinson, 347580), anti-ß-Tubulin (Santa Cruz Biotechnologies, 5274), anti-Ty1 (Diagenode, MAb-054-050).

### Gene Expression Analysis

Total RNA was isolated using the RNAeasy Minikit (Qiagen) according to the manufacturer’s instructions. cDNA was synthesized using the TaqMan Reverse Transcription kit (Applied Biosystems). qPCR was performed using SYBR Green 2× PCR Master mix (Applied Biosystems) on an ABI Prism 7300 Real-Time PCR system (Applied Biosystems) or on a LightCycler 480 System (Roche Applied Science), using the LightCycler 480 SYBR Green I Master mix (Roche Applied Science) according to the manufacturers’ instructions. Error bars represent standard deviation of three PCR amplifications for each sample. Similar results were obtained in at least three independent experiments. Primer sequences are provided in Table 1.

**Table 1:** Mouse expression and ChIP primers.

Expression primers	Primer Fw 5′–3′	Primer Rv 5′–3′
Oct4	GAGGAGTCCCAGGACATGAA	AGATGGTGGTCTGGCTGAAC
Nanog	AGGCTGATTTGGTTGGTGTC	CCAGGAAGACCCACACTCA
Sox2	ACAGATGCAACCGATGCACC	TGGAGTTGTACTGCAGGGCG
Msi1	CCATGCTGATGTTCGACAAAAC	TCAAACGTGACAAATCCAAACC
Sox1	CAAGATGCACAACTCGGAGATC	CTCGGACATGACCTTCCACTC
Otx2	GGTATGGACTTGCTGCATCC	CTTCCAGAACGTCGAGCTGT
Pax6	CATGGCAAACAACCTGCCTAG	GCACGAGTATGAGGAGGTCTGAC
Tubb3	ACTTGGAACCTGGAACCATGG	GGCCTGAATAGGTGTCCAAAGG
Gfap	CTCAATGACCGCTTTGCTAGC	CCTTGTTTTGCTGTTCCAGGAAG
Nestin	TTCCTGAGGTCTCCAGAAGC	GCCATCTGCTCCTCTTTCAC
T	GCTCTAAGGAACCACCGGTCATC	ATGGGACTGCAGCATGGACAG
Flk1	CCTGGTCAAACAGCTCATCA	AAGCGTCTGCCTCAATCACT
Gata4	GTGGCCTCTATCACAAGATGAAC	GTGGTGGTAGTCTGGCAGTTG
Sall4	AACAAATGCTGTGCCGAGTTC	TGCAACTTTTCTTGTGTTCCATG
FoxA2	GATGGAAGGGCACGAGCC	GTATGTGTTCATGCCATTCATCCC
Pax3	TCCCATGGTTGCGTCTCTAAG	CTCCACGTCAGGCGTTGTC
Utx	AAGGCTGTTCGCTGCTACG	GGATCGACATAAAGCACCTCC
Jmjd3	CTCTGGAAGTTTCATGCCGG	CTTAGCCCCATAGTTCCGTTTG
Uty	TGGTGCATCCAACCTAACTG	TGGCCCTGATCGACTGTAAT
rpPO	TTCATTGTGGGAGCAGAC	CAGCAGTTTCTCCAGAGC
Afp	AGTGCGTGACGGAGAAGAAT	TGTCTGGAAGCACTCCTCCT
Sox17	ACGGAATTCGAACAGTATCTGCC	CCTGGTAGGGAAGACCCATCTC
Hoxb1	CATCAGCCTACGACCTCCTC	GCACGGCTCAGGTATTTGTT
**ChIP primers**	**Primer Fw 5′–3′**	**Primer Rv 5′–3′**
Hoxb1	CTCTGGTCCCTTCTTTCC	GGCCAGAGTTTGGCAGTC
T	GCAGGGACCCAGGTGTAAT	CAGGTGGTCCACTCGGTACT
Flk1	GCATACCGCCTCTGTGACTT	GAGTGGGCTTCTTACCCACA
Msi1	GGGCACTGAGCTATCTCCAA	CCTAGTCCTTCCTCCCTGGT
Sox1	TGCACCTGTTTGCACAGTTCA G	GTGCACAAACCACTTGCCAAA G
FoxA1	CTAGCCCATCTCCTGCTGTC	TAAAGGAAGGGACTCCACCA

### ChIP Assays

ChIP analyses were performed as described [20]. Briefly, cells were fixed in 1% formaldehyde/PBS for 10 min. Then they were blocked with 0.125 M glycine for 5 min, washed extensively in PBS, collected in SDS buffer, pelleted and re-suspended in IP buffer. Samples were sonicated with the Diagenode Bioruptor in 1.3 ml for 8 min at high power and the sonicated chromatin controlled on 2% agarose gels. The DNA was sonicated to 700–400 bp in all experiments. For each IP, 1 mg of chromatin was used. Primary antibodies were incubated overnight at 4°C on a rotating platform. To each sample, 30 µl of 50% slurry of protein A-Sepharose (GE Healthcare) beads were added for 2–3 h. Beads were washed three times in 150 mM wash buffer and one time in 500 mM wash buffer. Beads (and input samples) were re-suspended in 120 µl of 0.1% SDS, 0.1 M NaHCO3 buffer and de-cross-linked at 65°C for a minimum of 3 h. DNA was purified using Qiagen PCR purification kit following the manufacturer’s instruction and eluted in 200 µl of H2O. Eluted material of 1–2 µl was used for each real-time quantitative PCR (qPCR) reaction. Primer sequences are provided in Table 1.

### Flow Cytometry

Cells were fixed in 70% ethanol and stained with primary antibody for 1 h, followed by 1 h incubation with Alexa Fluor 488 or 647 anti-rabbit (Invitrogen). Cells were pulsed with 33 µM bromodeoxyuridine (BrdU) for 30 min. DNA was counterstained by 0.1 mg/ml propidium iodide supplemented with RNase for 1 h at 37°C. Analysis was performed on a FACS Calibur using CellQuest software (BD). Quantification and analysis of cell-cycle profiles were obtained using FlowJo (Tree Star, Inc).

### Cumulative Growth Curve

3×10^5^ ESCs were seeded in a 6 cm-dish during 48 h, and then ESCs were trypsinized and counted with a counting chamber. 3×10^5^ cells were seeded again in another 6-cm dish. This process was repeated 3 more times. Cumulative population doublings at each sub-cultivation was calculated and added to the previous population doubling level, to yield the cumulative population doubling level.

### Embryoid Body Histology

Histological analysis was performed according to standard procedures. Briefly, ten days differentiated embryoid bodies were embedded in OCT (Sakura, 25608-930) and frozen for cryosectioning. Cuts of 8–10 µM sections were deposited over a super frost plus slide (VWR-Menzel, 631–9483) and stained with hematoxylin and eosin, dehydrated and mounted using VectaMount (Vector).

## Results

### Generation of Utx Conditional Construct and Knockout ESCs

**Figure 1:**
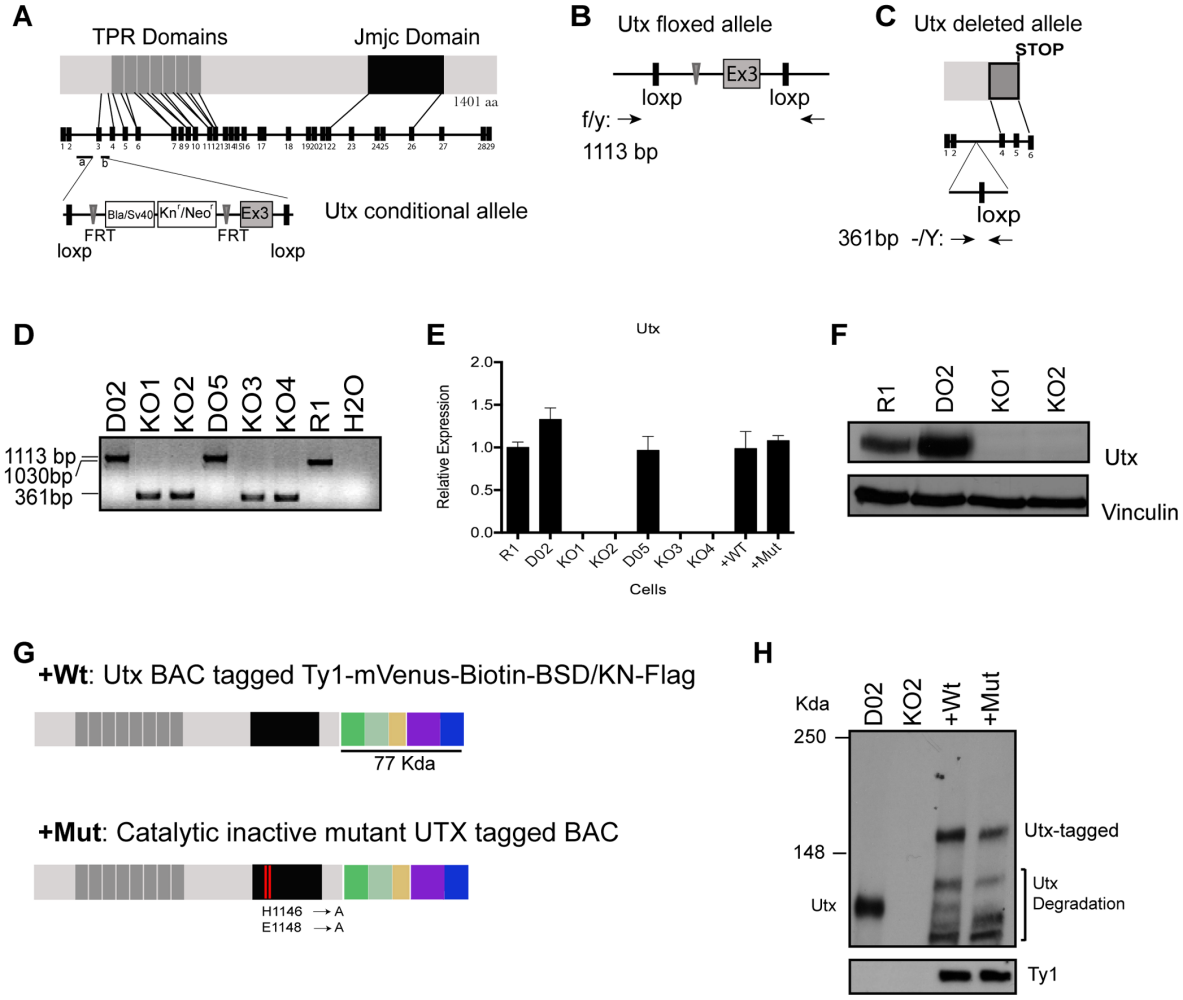
Generation of Utx knockout ESCs, and knockout ESCs complemented with wild type and catalytic mutant Utx. (A) Overview of the functional domains in Utx, the genomic locus of *Utx*, and the conditional targeting cassette for exon 3. (B) Predicted *Utx* floxed allele showing targeted cassette after treating targeted Utx clones with Flp recombinase. (C) Predicted *Utx* locus and deleted allele after treating with Cre recombinase. (D) Genotyping of *Utx* locus in ESCs after Flp and Cre recombination: agarose gel showing PCR amplification of wild type untargeted allele (R1∶1030 bp), floxed allele (D02, D05∶1113 bp) and deleted allele ESCs (KO Clones 1–4∶361 bp). (E) *Utx* expression levels determined by quantitative RT-PCR analysis (normalized to *Rplp0* and the levels expressed in the R1 ESC line). (F) Western blot analysis of Utx expressed in WT, floxed, KO1 and KO2 ESCs. Vinculin served as a loading control. (G) Schematic representation of wild type and catalytic inactive mutant BAC proteins tagged with two copies of the Ty1 peptide, the Venus fluorescence protein, a biotin tag, two rox sites surrounding the coding regions of Blasticidin/Kanamycin resistance gene and three copies of the flag tag. (H) Immunoblot showing endogenous and tagged Utx levels and Ty1 expression in floxed (D02), knockout clone (KO2), wild type Utx BAC (+WT) and catalytic mutant Utx BAC (+Mut).

To study the function of Utx in ESC self-renewal and differentiation, we generated a Utx conditional construct that was used to target male 129/Sv mouse derived R1 ESCs, introducing two loxP sites surrounding exon 3 (Fig. 1A and Fig. S1). Targeted cells were treated with Flp recombinase to generate floxed cells (D02 and D05) (Fig. 1B). Finally, after Cre recombinase transfection we obtained Utx knockout ESCs (Fig. 1C). Floxed and knockout clones were karyotyped and genotyped (Fig. 1D), and the absence of Utx in four mutant clones (KO1, KO2, KO3, KO4) were confirmed by RT-qPCR and western blot analysis (Fig. 1E–F and Fig. S2A–B).

### Re-introduction of Utx Wild Type and Catalytic Inactive Mutant into Knockout ESCs

For the analysis of Utx function we also generated cells expressing wild type Utx or catalytic inactive mutant in Utx KO ESCs by introducing BAC transgenes encoding a tagged version of the wild type and mutated *Utx* gene (Fig. 1G). The BACs were electroporated into Utx knockout cells and positive ESCs clones were selected and shown to express similar amounts of Utx as wild type ESCs (Fig. 1H).

### Characterization of Utx Knockout ESCs

**Figure 2:**
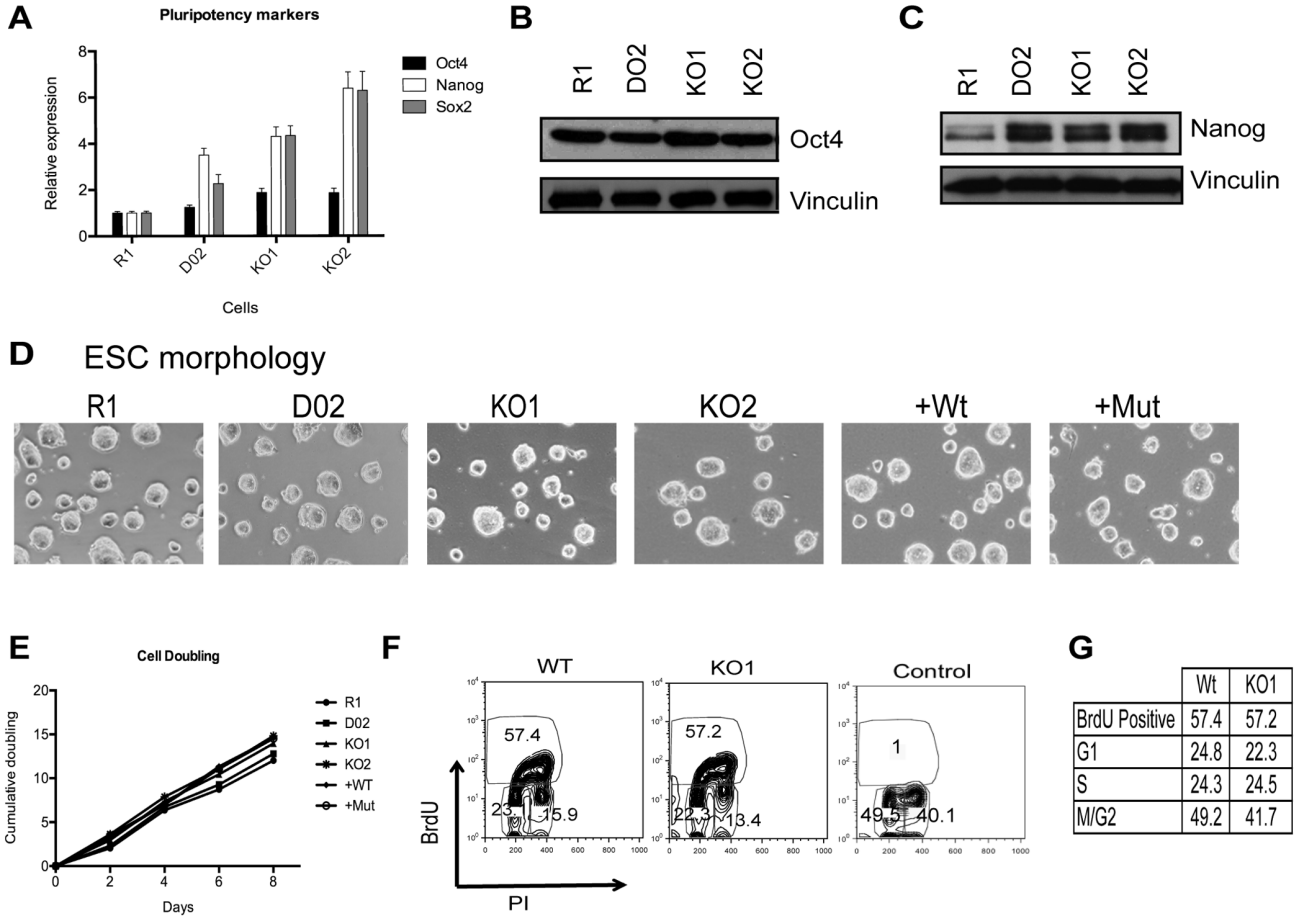
Utx is not required for ESC proliferation. (A) mRNA expression levels of Oct4, Nanog and Sox2. RT-qPCR normalized for Rplp0 and R1. (B–C) Immunoblots showing Oct4 and Nanog levels. Vinculin was used as loading control. (D) Morphology of Controls (R1, D02), KO (KO1, KO2) and complemented (+WT, +Mut) ESCs. (E) Cell proliferation analysis of the indicated ESCs measured at the indicated days. (F) DNA/BrdU flow cytometry analysis of WT (D02) and KO1 cells. Control represents cells without BrdU pulsing. PI is propidium iodine. (G) Cell cycle analysis by flow cytometry of WT and KO1.

Utx is highly expressed in mouse ESCs (Fig. 1E–F, Fig. S2A–B). The deletion of Utx and the re-expression of wild type (+Wt) and catalytic inactive mutant (+Mut) Utx in the knockout cells did not compromise the expression of the pluripotency markers Oct4, Nanog or Sox2 (Fig. 2A–C and Fig. S2C–D) or ESC morphology (Fig. 2D and Fig. S2E. Moreover, Utx KO ESCs, +Wt and +Mut, have similar cell cycle distribution and proliferation rate as floxed (D02, D05) and Wt (R1) ESCs (Fig. 2E–F and Fig. S2F–H). Importantly, transcription across the *Utx* locus in knockout ESCs was absent (Fig. S2J), indicating that no truncated or alternatively spliced Utx protein can be produced. Taken together these results demonstrate that Utx is not required for ESC proliferation.

### Utx is Required for Proper Expression of Ectoderm and Mesoderm Marker Genes during ESC Differentiation

Next, we assessed the involvement of Utx in ESC differentiation in vitro using two well-described differentiation strategies: 1) All-trans retinoic acid (RA) treatment to induce monolayer differentiation of ESCs and 2) Spontaneous embryoid body (EB) formation. In these assays we did not observe any difference in phenotypes between floxed D02, D05 and wild type R1 ESCs, and we therefore only included results from the floxed D02 or D05 as controls.

**Figure 3:**
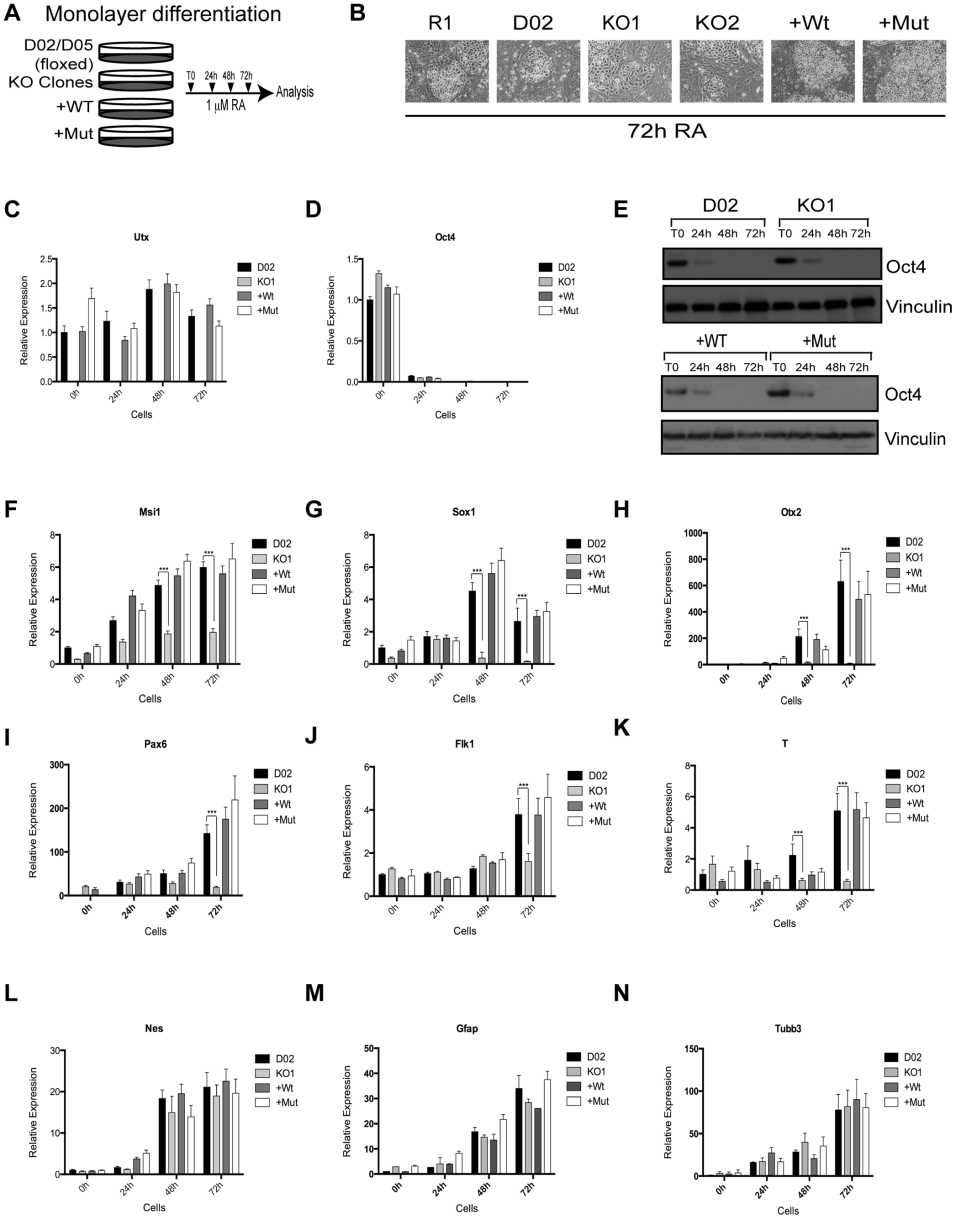
Utx regulates timely activation of developmental regulators during differentiation. (A) Scheme of ESC differentiation. Samples were taken 24, 48 and 72 hours after RA treatment and analyzed by RT-qPCR. All RT-qPCRs were normalized to Rplp0 and the levels in D02 at T0 (B) ESC morphology 72 h after RA treatment. (C) The expression of Utx mRNA during the time course (D, E) Pluripotency marker Oct4 mRNA and protein expression levels during differentiation. Vinculin was used as loading control. (F–N) Expression analysis of the indicated genes during differentiation. Ectoderm markers: Msi1, Sox1, Otx2, Pax6, Nes, Gfap, Tubb3; mesoderm markers: Flk1, T. Error bars represent SD, n = 3 independent assays (***p<0.0005, two tailed Student’s test).

**Figure 4:**
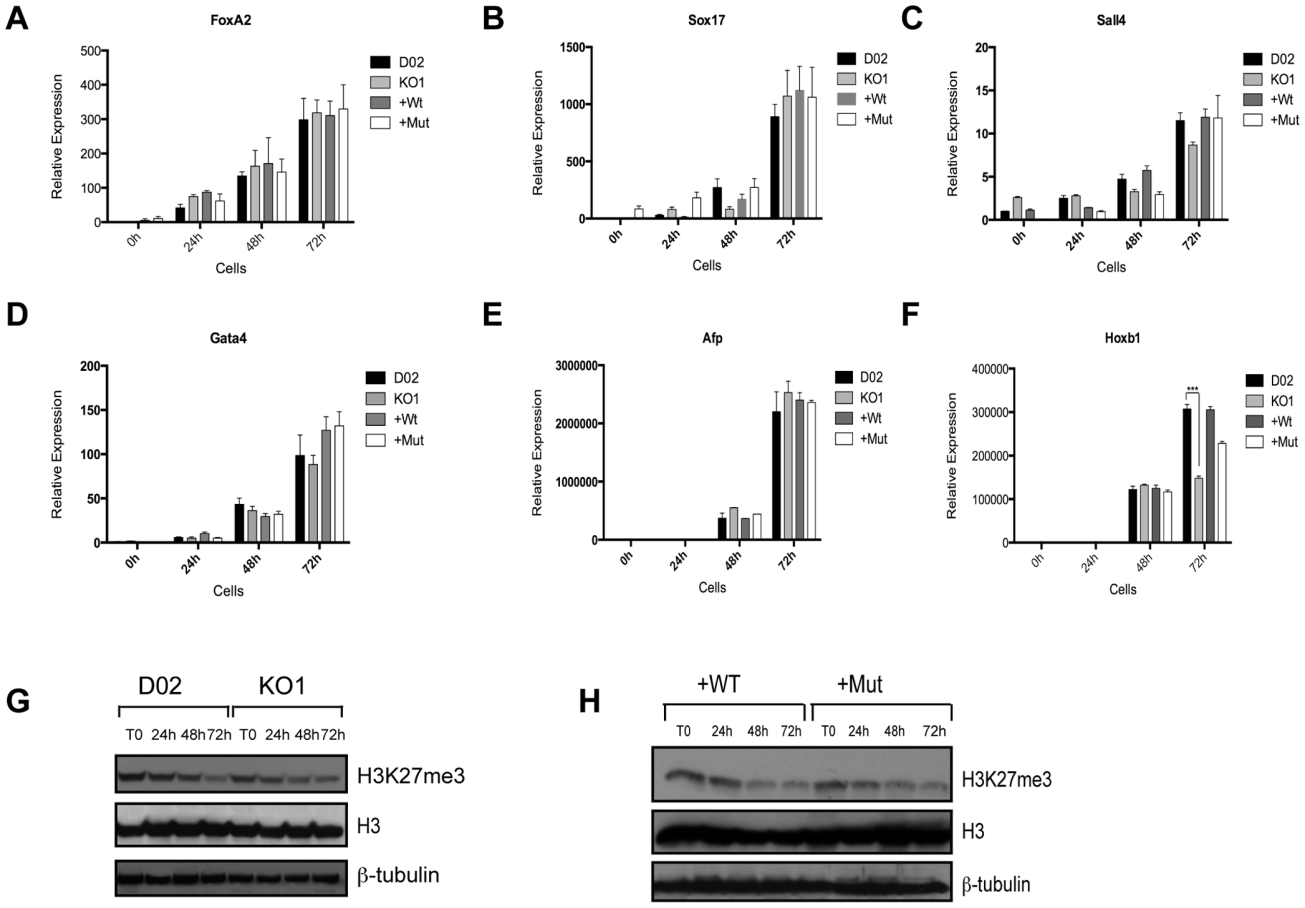
Utx is important for normal ESC differentiation. (A–F) Expression analysis of endoderm markers FoxA2, Sox17, Sall4, Gata4, Afp and homeobox gene Hoxb1. Error bars represent SD, n = 3 independent assays (***p<0.0005, two tailed Student’s test). (G–H) H3K27me3 protein levels during differentiation. ß-tubulin and H3 were used as loading controls.

For the differentiation of ESCs in monolayer, the ESCs were treated with 1 µM RA for 24, 48 and 72 hours (Fig. 3A). Using this treatment, we did not observe any morphological changes in the Utx knockout, +Wt, or +Mut, when compared to control cells (Fig. 3B and Fig. S3A). Although Utx expression levels have been reported to decrease during differentiation along with Nanog and Oct4 [38], we did not detect any decrease in Utx expression (Fig. 3C and Fig. S3B). Moreover, Oct4 expression was completely lost 48 h after RA treatment (Fig. 3D–E and Fig. S3C–D). Interestingly, the lack of Utx resulted in a general de-regulation of the normal expression pattern of some ectoderm and mesoderm markers during differentiation, which was rescued by the re-expression of both wild type and catalytic inactive Utx. Specifically the ectoderm markers Musashi 1 (Msi1), SRY-box containing gene 1 (Sox1), Orthodenticle homolog 2 (Otx2), Paired box gene 6 (Pax6) (Fig. 3F–I and Fig. S3E–F) and mesoderm markers vascular endothelial growth factor receptor 2 (Flk1/Kdr/Vegfr2) and Brachyury (T) (Fig. 3J–K and Fig. S3G–H) were not induced to the same extent as in wild type ESCs. However, Nestin (Nes), Glia fibrillary acidic protein (Gfap) and Beta III Tubulin (Tubb3), other ectoderm markers (Fig. 3L–N, Fig. S3I), and endoderm markers Forkhead box A2 (FoxA2), SRY-box containing gene 17 (Sox17), Sal-like 4 (Sall4), Gata binding protein 4 (Gata4) and Alpha fetoprotein (Afp) were expressed at the same levels as in wild type ESCs during differentiation (Fig. 4A–E and Fig. S3J). These results suggest that Utx plays a critical role in activating the expression of certain genes involved in ectoderm and mesoderm differentiation independently of its catalytic activity.

Hoxb1 is a member of the homeobox transcription factor family that confers tissue identity along the anterior-posterior axis during mouse and human development. Interestingly, full activation of Hoxb1 also requires Utx, and although the levels are not completely rescued by the re-expression of catalytic inactive Utx, the expression is higher than in knockout cells (Fig. 4F and Fig. S4A). These findings are in accordance with previous reports [31], [32], where Utx catalytic activity is necessary for the activation of some homeotic genes during ESC differentiation. Importantly, the global level of H3K27me3 was decreased to a similar extent in all the ESCs analyzed, and the decrease was therefore not dependent on Utx (Fig. 4G–H and Fig. S4B).

**Figure 5:**
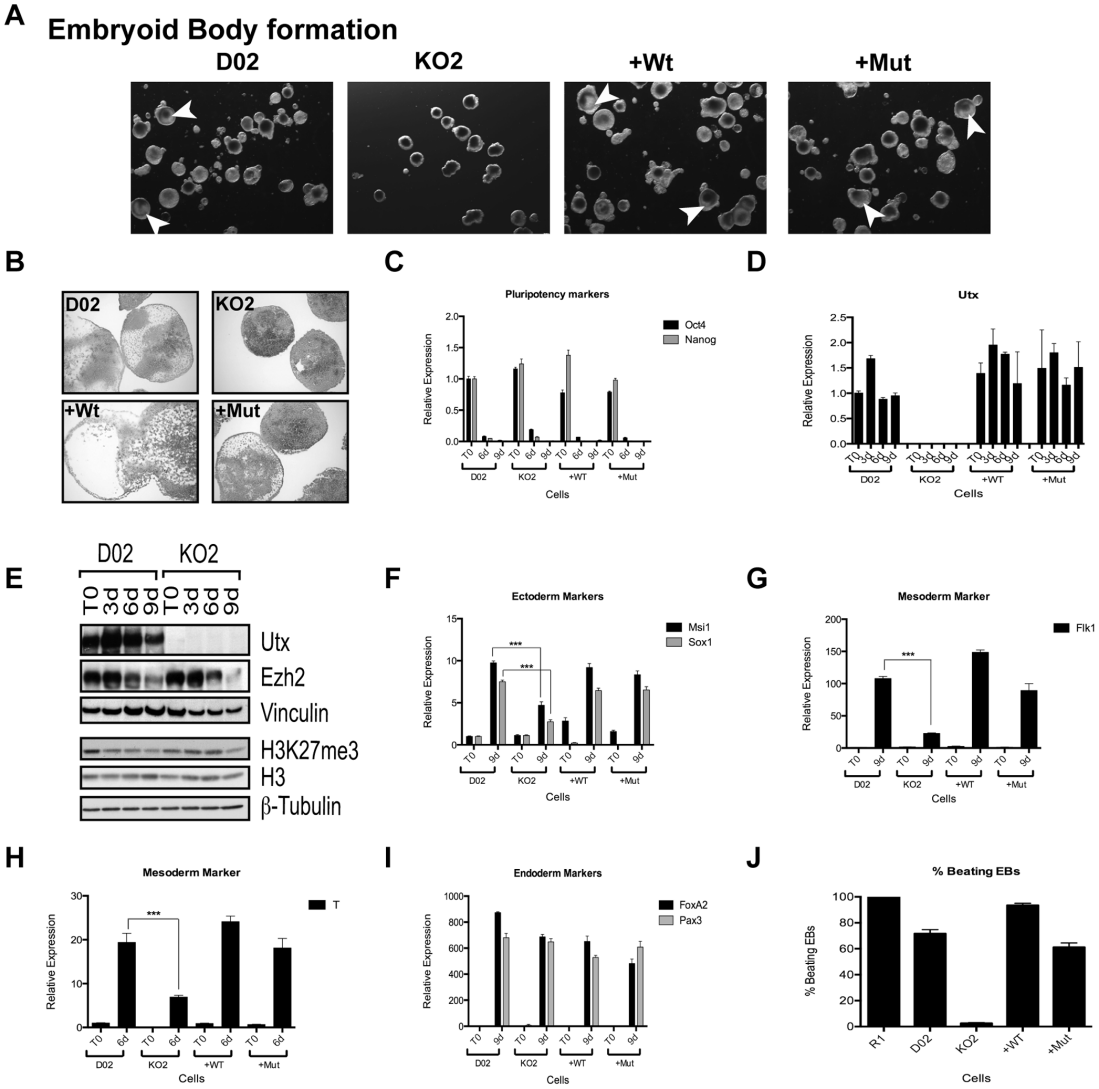
Utx is required for proper differentiation of ESCs. (A) Morphology of embryoid bodies 9 days after formation using the indicated ESCs as a starting material. White arrowheads depict some internal cavitation. (B) H&E staining of EBs harvested at day 10 post differentiation. (C) Pluripotency markers Oct4 and Nanog expression after 6 and 9 days of differentiation. (D) Utx expression levels before and after 3, 6 and 9 days of EB differentiation. (E) Western blot for Utx, Ezh2 and H3K27me3 during EB formation of ESCs. Vinculin, H3 and ß-tubulin were used as loading controls. (F, G, I) Gene activation of ectodermal (Msi1, Sox1), mesodermal (Flk1) and endodermal (FoxA2, Pax3) markers after 9 days of EB differentiation. (H) Expression levels of mesodermal marker Brachyury after 6 days of differentiation. All RT-qPCRs were normalized to the expression in D02 at T0 and Rplp0. (J) R1, D02, KO2, +Wt and +Mut percentage of beating EBs after EB formation and cardiac lineage differentiation. Error bars represent SD, n = 3 independent assays (***p<0.0005, two tailed Student’s test)

EBs are the in vitro developmental equivalent of the mouse embryo at early developmental stages [46]. They appear as three-dimensional aggregates of pluripotent ESCs that undergo differentiation and cell specification into all three germ layers in the absence of LIF. Even though no differences in size were observed in Utx knockout EBs after 9 days of differentiation (Fig. 5A), they showed abnormal morphology with visible lack of internal organized structures, characteristic of defective EB formation. This appearance of dense mass of cells observed at Utx knockout EBs disappeared, when wild type or catalytic mutant versions of Utx were re-expressed (Fig. 5A). Hematoxylin-Eosin staining of EB sections confirmed a diminished mesoderm and ectoderm differentiation potential indicated as the lack of internal cavitation in Utx knockout EBs (Fig. 5B). Pluripotency markers Oct4 and Nanog decreased as expected, during differentiation (Fig. 5C). In agreement with the results for RA-differentiated cells, Utx levels did not decrease during spontaneous EB formation (Fig. 5D). In contrast, Utx levels showed a transient increase during EB formation (Fig. 5D–E). During the differentiation process the protein levels of EZH2, the major H3K27me3 methyltransferase was decreased as were the global levels of H3K27me3. However, in contrast to the monolayer differentiation assay, the reduction of H3K27me3 levels was slower in *Utx* KO EBs as compared to wild type controls (Fig. 5E), suggesting that Utx contributes to this downregulation.

Similar.to the monolayer differentiation assay, Utx is also required for the proper activation of ectodermal (*Msi1*, *Sox1, Otx2 and Pax6*) and mesodermal (*T*, *Flk1*) genes during EB formation (Fig. 5F–H and Fig. S4C–D). This expression was rescued both by the re-expression of wild type and catalytic inactive Utx (Fig. 5F–H and Fig. S4C–D). In contrast, Utx is not required for the normal induction of ectodermal marker genes *Gfap* and *Tubb3* or endodermal genes, *FoxA2*, *Pax3, Sox17, Sall4, Gata4 and AFP* (Fig. 4I and Fig. S4E–J), and mouse ESCs therefore appear to have different requirements for Utx than human ESCs, in which UTX was recently shown to be required to differentiate to endoderm lineage [47].

To further understand the role of Utx in mesodermal differentiation, we differentiated wild type, floxed, Utx knockout, +Wt and +Mut ESCs into cardiomyocytes (CM), which exhibit spontaneous contractile activity. In agreement with our previous results, Utx KO cells failed to differentiate into cardiomyocytes (mesoderm derived lineage) and did not produce contractile cells (Fig. 5J).

In summary, our results show that Utx is required for ectoderm and mesoderm differentiation in vitro, independently of its catalytic activity. However, a subset of genes, like Hoxb1, requires the catalytic activity of Utx for their normal activation during differentiation [31], [32].

### Utx Binds to and Regulates Developmental Genes

**Figure 6:**
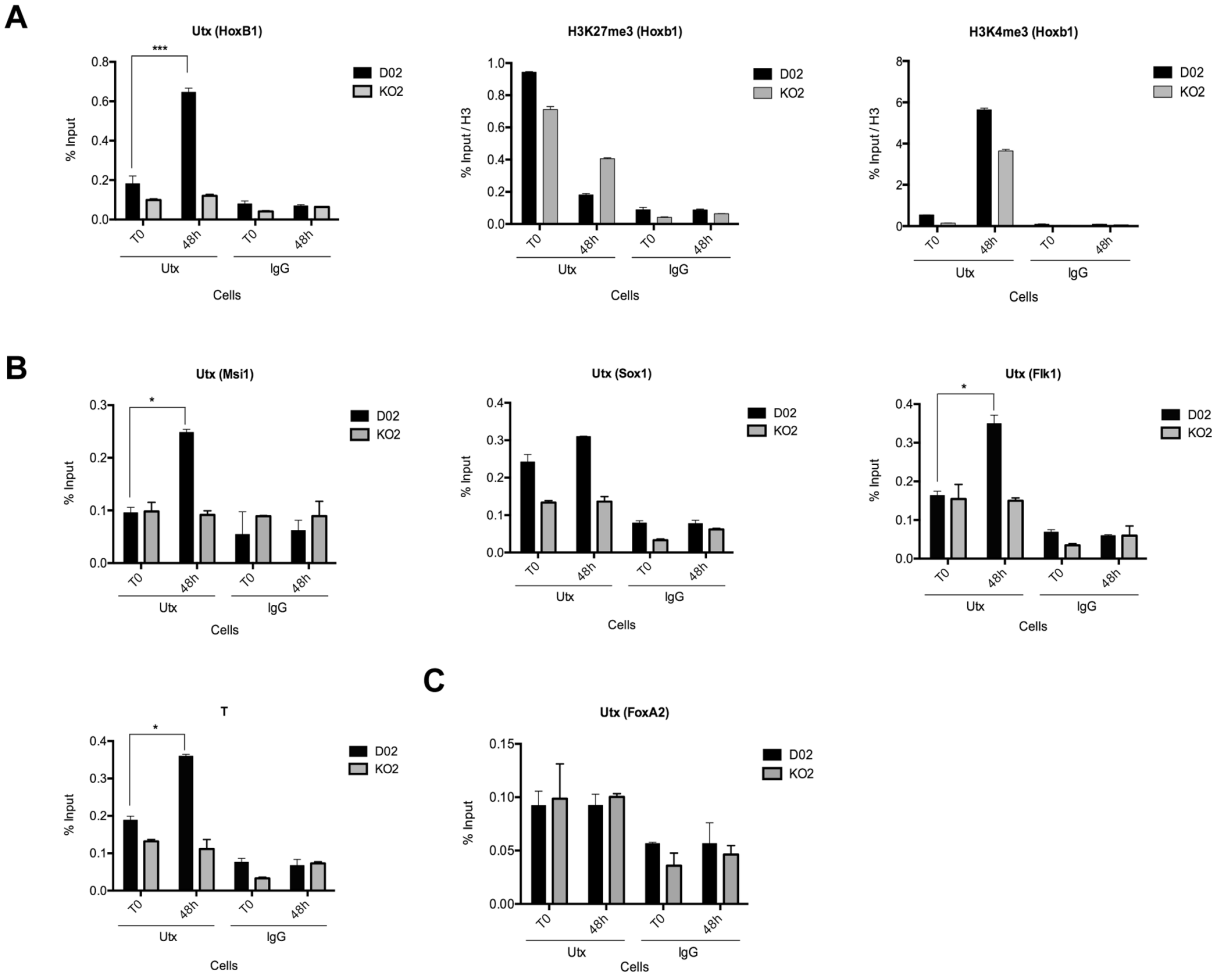
Utx binds to promoter regions of developmental genes. (A) ChIP assays of Utx and the indicated histone modifications on the *Hoxb1* promoter at the indicated times during differentiation. (B–F) Utx binding to promoter regions of ectoderm (*Msi1*, *Sox1*); mesoderm (*Flk1*, *T*) and endoderm (*FoxA2*) developmental regulators as assayed by ChIP. “% input” represents (bound/input material x 100). Error bars represent SD, n = 3 independent assays. (*p<0.05; ***p<0.0005, two tailed Student’s test).

To investigate the mechanism by which Utx regulates ESC differentiation, we tested if Utx binds to genes involved in ectoderm, mesoderm and endoderm differentiation. As a control we showed that Utx is recruited to the previously identified Utx target gene, *Hoxb1* during differentiation [31], [32]. Moreover, we showed that H3K27me3 levels decreased and H3K4me3 levels increased at the Hoxb1 promoter after differentiation in both wild type and knockout cell, although the changes are much lower in the knockout cells (Fig. 6A). Similarly, Utx was also found to be recruited to promoters of genes involved in ectoderm (Msi1, Sox1) and mesoderm (T, Flk1) differentiation, but was not found associated with the promoter of the endoderm gene *FoxA2* (Fig. 6B–C). Consistent with the catalytic activity of Utx not being required for the regulation of ectoderm and mesoderm genes we did not observe any changes in H3K27me3 of these genes during differentiation (Fig. S5A–E). In contrast, H3K4me3 levels increased after differentiation in wild type ESCs, which was completely absent in *Utx* KO ESCs (Fig. S5A–E), demonstrating that Utx, most likely by recruiting the histone methyltransferases Mll3 and Mll4, is required for H3K4 methylation of ectoderm and mesoderm promoters during differentiation.

### Uty and Jmjd3 Contribute to the Regulation of Ectoderm, Mesoderm and Homeotic Genes during Differentiation of Mouse ESCs

**Figure 7:**
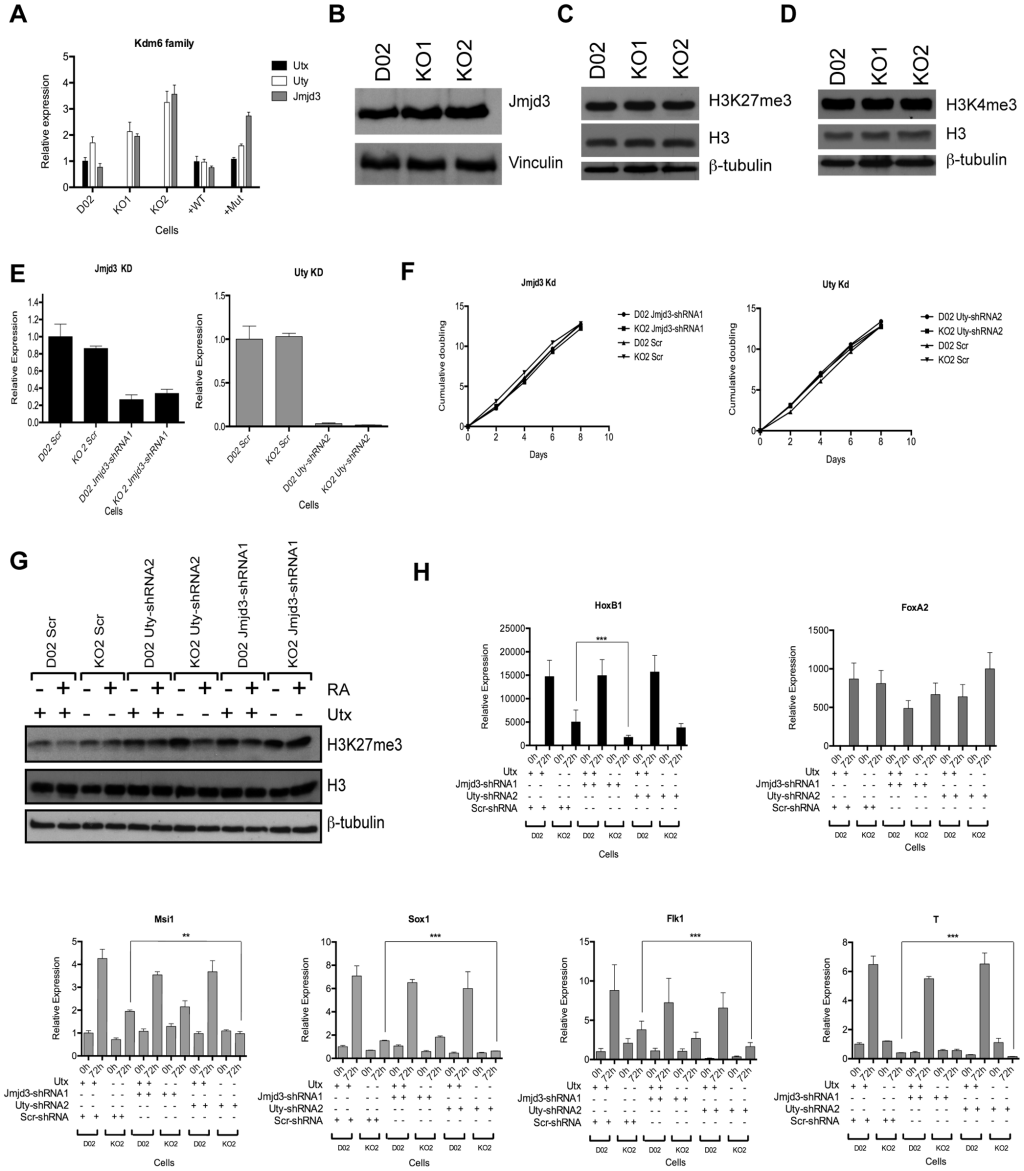
Jmjd3 and Uty contribute to the regulation of developmental genes during differentiation. (A) The expression levels of the Kdm6 family in ESCs measured by RT-qPCR and normalized to Rplp0 and D02. (B) Expression levels of Jmjd3 in D02, KO1, and KO2 ESCs. Vinculin was used as loading control. (C–D) Western blots showing the H3K27me3 and H3K4me3 levels in the indicated cell lines. ß-tubulin and H3 were used as loading controls. (E) The efficiency of Jmjd3-shRNA1 and Uty-shRNA2 knockdown in the indicated cell lines as measured by RT-qPCR and normalized to Rplp0. (F) Cell proliferation analysis of the indicated cells lines. (G) Western blot showing H3K27m3 levels in the indicated cell lines before and after 72h of RA differentiation (H) mRNA expression levels of Utx target genes in Utx knockout (KO2) cells with and without knocking down Jmjd3 or Uty knockdown cells. All RT-qPCRs were normalized to Rplp0 and the expression levels in D02 Scr at T0. Error bars represent SD, n = 3 independent assays (**p<0.005; ***p<0.0005, two tailed Student’s test).

Deletion of *Utx* leads to a slight increase in Uty and Jmjd3 expression levels (Fig. 7A–B and Fig. S2A–B). Since the loss of Utx does not affect the global levels of H3K27me3 (Fig. 7C–D and Fig. S2I), it could suggest that Uty and Jmjd3 might partially compensate for Utx loss.

To address this, we knocked down (kd) the expression of Jmjd3 or Uty in floxed (DO2) and *Utx* KO ESCs (Fig. 7E and Fig. S6A). The knockdown of Jmjd3 and Uty did not affect cell viability or proliferation (Fig. 7F). Moreover, the H3K27me3 global levels did not change in Jmjd3 kd and Uty kd ESCs, indicating that the PRC2 complex is the major determinant of global H3K27me3 levels in mouse ESCs. However, the reduction of H3K27me3 levels in Jmjd3 kd ESCs during differentiation was not as pronounced as in wild type ESCs (Fig. 7G and Fig. S6B). Interestingly, the expression level of homeotic gene Hoxb1, show a slight but significant decrease in Utx KO ESCs treated with RA in which Jmjd3 was downregulated as compared to Utx KO ESCs. Moreover, when Uty is downregulated in Utx knockout cells, the expression levels of genes specific for ectodermal and mesodermal tissues, also showed a small but significant reduction when compared with Utx KO ESCs (Fig. 7H and Fig. S6C). Taken together these results suggest that Uty and Jmjd3 partially compensate for the loss of Utx during ESC differentiation.

## Discussion

In this study we demonstrate that Utx is dispensable for ESC proliferation and that its deletion does not lead to a global increase in H3K27me3 levels. Moreover, we show that Utx is required for the proper differentiation of ESCs into ectoderm and mesoderm, independently of its catalytic activity. Our results show that Utx is required for the expression of several genes involved in regulating differentiation, and the binding of Utx to the promoters of these suggest that it directly regulates their transcription. While the catalytic activity of Utx is not required for the regulation of several genes involved in ectoderm and mesoderm specification, the catalytic activity of Utx is required for the activation of a subset of genes, including *Hoxb1*.

### Utx Binds to the Promoter Regions of Developmental Genes and Regulates their Expression during Differentiation

The expression of Utx is maintained during ESC differentiation, and in agreement with the notion that regulation of chromatin structure by posttranslational modifications is important for regulating gene expression during differentiation, we have shown that Utx is a critical regulator of this process. Interestingly, we found that while the expression level of pluripotency marker Oct4 is decreased in Utx knockout ESCs undergoing differentiation, several differentiation markers were not induced. This suggests that Utx is not essential for initiating the differentiation program in ESCs, but for the activation of genes contributing to differentiation.

Specifically, we have shown that genes involved in ectoderm formation such as *Msi1*, *Sox1, Otx2 and Pax6* are only marginally activated by differentiation signals in *Utx* KO ESCs when compared to wild type ESCs, whereas *Nes*, *Gfap*, *Tubb3* levels, consistent with a previous report [38], are not affected. These results suggest that Utx, although it does not regulate all ectodermal genes, is essential for the de-repression and/or activation of some RA-inducible genes involved in the formation of neuroectoderm.

During vertebrate development, the formation of the central nervous system (CNS) and neural plate (future neural tube) from a region of the primitive ectoderm is a result of the activation of specific genes, which in turn promote the formation of the nervous system. Otx2 is a member of the bicoid sub-family of home domain-containing transcription factors. Otx2 protein plays an important role in brain and sensory organ development. It is activated in the entire ectoderm before gastrulation, and is one of the earliest genes expressed in the anterior neuroectoderm, demarcating rostral brain regions [48], [49]. Mutant mice homozygous for Otx2 are early embryonic lethal with their forebrain and midbrain regions are deleted due to a defective anterior neuroectoderm specification during gastrulation [50]–[52]. Pax6 is a member of the murine paired-box-containing gene family. At embryonic day 8, Pax6 is expressed in discrete regions of the forebrain and the hindbrain. It is expressed in the ventral neural tube before neural differentiation starts and plays a role in the regional specification of cells in the neural tube with respect to the dorsal-ventral axis. Pax6 is also expressed in the developing pituitary, the olfactory epithelium and in the developing eye and has an important regulatory role in the development of the main structures of the eye [53], [54]. Small eye mice homozygous for mutations in the *Pax6* gene showed severe CNS abnormalities including absence of lenses and nasal cavities [55], defects in forebrain patterning [56]–[58], axonal path finding [59], and motor neuron and glial cell subtype specification [60]–[63]. Sox1 is one of the earliest transcription factors expressed in ectodermal cells committed to neural fate. It is involved in maintenance of neural progenitor identity, and it also promotes neuronal lineage commitment [64]. Sox1 expression is restricted to neuroectoderm in the mouse embryo [65] and although Sox1-deficient mice are viable, they exhibit lens defects and suffer from spontaneous epilepsy seizures associated with abnormal forebrain development and olfactory cortex hyper-excitability [66], [67]. Musashi1 (*Msi1*) is an RNA-binding protein that regulates gene expression at the post-transcriptional level, and its expression pattern is conserved among different species, including fly, worms and humans [68]. Musashi is strongly expressed in the central nervous system in zebra fish and mouse, especially in neural stem cells of the mouse embryo around the ventricular zone of the neural tube [69]–[73]. *Msi1* is not essential for embryonic viability; however, Msi1 knockout animals develop hydrocephaly [74]. We have shown that Utx binds to the promoter regions of *Msi1* and *Sox1*, and is required for *Otx2*, *Pax6*, *Msi1* and *Sox1* expression in a demethylase independent manner. Utx might instead contribute to the activation of these promoters by regulating the H3K4 methylation activity of the MLL3/4 complex, as has previously been suggested for the *C. elegans* homologue [35] or by recruiting a chromatin remodeling complex. The requirement for Utx for the activation of *Otx2*, *Pax6*, *Msi1* and *Sox1* during differentiation might, in addition to the effects observed in vitro, also explain the neural tube closure defects observed in *Utx* knockout mice [29], [30], [37], [38].

We have also shown that Utx is enriched at the Flk1/Kdr/Vegfr2 and Brachyury (T) promoters during ESC differentiation, and that Utx is required for the activation of these two genes. Flk1 is a type III transmembrane kinase receptor that plays a critical role in vascular endothelial cell development. Flk1-deficient mice die in utero between E8.5 and E9.5 due to defective development of yolk sac blood islands, endothelial and hematopoietic cells [75]–[77]. Importantly, Shpargel et al. [30] recently showed that *Utx* knockout mice exhibit a reduction in the yolk sac vasculature and deficient hematopoiesis. Moreover, Liu et al. [78] have recently demonstrated that Utx plays an important role in normal and malignant hematopoiesis. Since both these phenotypes are pheno-copied in *Flk1* knockout mice, this might suggest that lower expression levels of Flk1 could explain the yolk sac and hematopoietic defects in the Utx KO mice during development. Moreover, a recent report has shown that Vegf promotes cardiomyocyte differentiation predominantly by Erk-mediated Flk1 activation in ESCs [79]. Since we, in agreement with a previous publication [37], have shown that Utx KO ESCs are completely unable to acquire the contractile characteristics of cardiomyocytes when differentiated, this phenotype could also been explained by the lack of Flk1 expression in Utx KO ESCs during differentiation.

Brachyury (T) is a DNA binding protein that functions as a transcription factor and form part of the T-Box family of proteins. In mice, Brachyury affects the development of the posterior mesoderm during gastrulation in a dose dependent-manner. Homozygous mutant mice die around E10 dpc with a general failure in notochord morphogenesis [80]. Interestingly, Brachyury is also required for the early expression of *Nodal* and the proper left sided expression of *lefty-1* and *lefty-2;* all three genes are known to be important for the left-right asymmetry during heart formation [81]. Thus, at embryonic day 9.5 Brachyury mutant mice show structurally abnormal hearts with randomized heart looping and orientation and a consistent and aberrant expression of both lefty genes and nodal [81]. Importantly, *Utx* knockout mice have been described to display posterior truncation and severe cardiac malformations, which include linear heart tube (unable to loop) and deficient chamber development [29], [30], [37], [38]. These phenotypes are very similar to those found in homozygous Brachyury mutant mice.

In summary, based on the observation that Utx is required for the activation of *Otx2*, *Pax6*, *Msi1*, *Sox1*, *Flk1* and *T* during differentiation of ESCs in vitro, and the phenotypes of the mutant mice described above, we propose that Utx is also required for the regulation of these genes during normal embryonic development.

### Potential Overlapping Functions of Utx, Jmjd3 and Uty

Our analysis of gene expression during ESC differentiation also showed that Uty and Jmjd3 contribute to the regulation of tissue specific genes, and that they might partially compensate for the loss of Utx in a demethylase-independent and -dependent manner, respectively. Nevertheless, it has been shown that Jmjd3 also exhibits demethylase independent activity by recruiting chromatin remodeling complexes at the promoter of T-box genes [82], so perhaps not only Uty but also Jmjd3 could compensate for Utx loss in a demethylase independent way. The data for Uty is in line with several recent publications showing a more severe phenotype associated with knocking out Utx in female than in male mice [29], [30], [37], [38].

In summary we have shown that Utx is essential for normal ESC differentiation, that it is required for the activation of mesodermal and ectodermal genes independently of its catalytic activity. Our results suggest that Utx, Jmjd3 and Uty are dispensable for ESC proliferation and that they have partially overlapping functions during ESC differentiation. More in vivo experiments are required to completely to understand the function of this interesting family of proteins and its catalytic activity.

## Supporting Information

Figure S1 **The generation of Utx knockout ESCs.** (A) DNA long-range PCR analysis of selected ESC clones after the electroporation of the Utx targeting construct. The primers were designed to amplify a 3 kb fragment. One primer was located outside of the homology arm of the targeting construct, and the other one inside the targeting construct covering the closest loxp site, Vector and wild type R1 DNA were used as negative controls. (B) Agarose gel showing PCR amplification of a 215 bp band with primers designed to amplify the other loxP site, ensuring its integrity after homologous recombination. R1 wild type DNA was used as a negative control. (C) Analysis of a qPCR designed to amplify the Neomycin cassette at the targeted allele to ensure the integration of a unique copy of the construct at the ESCs genome. Karyotype analyses of the targeted ESCs guide us to select P1-E04 to proceed with the study. (D) Table collecting results from the analysis by long range PCR of three floxed clones (D02, D05, D06) obtained after flp recombinase electroporation and selection. Correct excision of Kanamycin-Neomycin resistance cassette is designated as HR and the absence of the neomycin-encoding gene as Neo-. (E) Graphics showing the karyotype analysis of the three floxed clones. Karyotype analyses were used to choose clones D02 and D05 (with 75% and 71% of metaphases showing 40 chromosomes, respectively) for further studies. (F) Representative metaphases for clone D02 and D05.
(TIF)

Figure S2 **Characterization of Utx KO clones.** (A) The expression of Kdm6 family members was determined by quantitative RT-PCR analysis (normalized to Rplp0 and to the expression in R1 ESCs at T0). (B) Utx and Jmjd3 protein levels in floxed and in KO clones 3 and 4 ESCs. Vinculin was used as loading control. (C) mRNA expression levels of the pluripotency markers Oct4, Nanog and Sox2 in the indicated clones. (D) Western blots showing Oct4 and Nanog levels in the indicated clones. Vinculin was used as loading control. (E) Morphology of ESCs in the indicated clones. (F) Cell proliferation assay of WT, floxed and KO ESCs cell at the indicated days after plating. (G) Flow cytometry analysis of WT (D02) and KO4 pulsed with BrdU and stained with anti-BrdU antibody. Control represents cells without BrdU pulsing. (H) Cell cycle analysis by flow cytometry of WT and KO clone stained with PI (propidium iodine). (I) Western blot analysis showing the levels of H3K27me3 and H3K4me3 in the indicated cell lines. ß-tubulin and H3 were used as loading controls (I) Utx expression levels in control D02 and KO2 ESCs determined by quantitative RT-PCR analysis (normalized to Rplp0 levels). Primers were designed to cover exons encoding the following functional domains: TPR 1–2 (E3–6), TPR 3–4 (E6–9), TPR 5–6 (E 9–11), TPR 7–8 (E 11–12), and JmjC (E22–26).
(TIF)

Figure S3 **Utx is required for normal ESC differentiation.** Time points were taken 24, 48 and 72 hours after treatment with 1 µM RA and analyzed by Western blotting or RT-qPCR. All qPCRs were normalized to Rplp0 and the level in D05 ESCs at T0. (A) The morphology of the indicated ESCs 72 h after addition of retinoic acid. (B) The levels of Utx mRNA expression during differentiation. (C, D) The expression of Oct 4 mRNA (C) and proteins levels (D) during RA-induced differentiation in the indicated cell lines. Vinculin was used as loading control. (E–J) mRNA expression analysis of the indicated genes during monolayer differentiation of control (D05) and Utx KO cells. Utx knockout cells transfected with wild type (+Wt) or catalytic mutant (+Mut) Utx BAC were also analyzed. Fold activation is presented for three ectodermal markers Msi1, Sox1, Nes; two mesodermal markers Flk1, T and one endoderm marker FoxA2. Error bars represent SD, n = 3 independent assays (***p<0.0005, two tailed Student’s test).
(TIF)

Figure S4 **Utx is important for normal ESC differentiation.** Monolayer differentiation: Time points were taken 24, 48 and 72 hours after treatment with 1 µM RA. Embryoid bodies formation: samples were taken before and after 3, 6 and 9 days of EB differentiation. Samples were analyzed by Western blotting or RT-qPCR. All qPCRs were normalized to Rplp0 and the level in D02 or D05 ESCs at T0. (A) mRNA expression analysis of Hoxb1 during RA-induced differentiation in D05, K04 ESCs. Utx knockout cells transfected with Wt (+Wt) or catalytic mutant (+Mut) Utx BAC were also analyzed. (B) Western blot of H3K27me3 levels in the indicated cell lines during differentiation. ß-tubulin and H3 were used as loading controls. (C–F) Expression levels of ectoderm markers Otx2, Pax6, Gfap and Tubb3 in the indicated cells. (G–J) mRNA analysis of endoderm markers Sox17, Sall4, Gata4 and Afp in D02, KO2, +Wt and +Mut ESCs. Error bars represent SD, n = 3 independent assays (***p<0.0005, two tailed Student’s test).
(TIF)

Figure S5 **H3K27me3 and H3K4me3 levels at the promoter regions of developmental genes.** (A–E) ChIP assays of the indicated histone modifications on the ectoderm (*Msi1, Sox1*); mesoderm (*Flk1, T*) and endoderm (*FoxA2*) promoters at the indicated times during differentiation. “% Input” represents (bound/input material x 100). Error bars represent SD, n = 3 independent assays.
(TIF)

Figure S6 **Overlapping functions of Utx, Jmjd3 and Uty.** (A) Levels of Jmjd3 and Uty after in the indicated ESCs expressing Jmjd3-shRNA3 and Uty-shRNA1 as measured by RT-qPCR and normalized to Rplp0. (B) Western blot showing H3K27m3 levels in the indicated cell lines before and after 72 h of RA differentiation. (C) mRNA expression levels of Utx target genes in Utx knockout (KO2) cells with and without knocking down Jmjd3 or Uty. All RT-qPCRs were normalized to Rplp0 and the expression levels in D02 Scr at T0. Error bars represent SD, n = 3 independent assays (**p<0.005; ***p<0.0005, two tailed Student’s test).
(TIF)
