# Correction: Utx Is Required for Proper Induction of Ectoderm and Mesoderm during Differentiation of Embryonic Stem Cells

**DOI:** 10.1371/journal.pone.0306360

**Published:** 2024-06-27

**Authors:** Cristina Morales Torres, Anne Laugesen, Kristian Helin

Following publication of this article, concerns were raised about images in Figs 1, 5, 7, and S2. Here the authors provide additional information, corrected figures, and underlying data for these experiments.

During the preparation of Fig 1D, the DO2 and DO5 lanes were erroneously duplicated. The DO2 lane is incorrect. An updated version of Fig 1 with the correct DO2 lane is provided here with splice lines clearly marked and including the correct DO2 lane. The full-length uncropped gel underlying Fig 1D is provided in S1 File.

In Fig 5E, the 3d, 6d, and 9d KO2 sample lanes appear similar. An updated version of Fig 5E is provided here where the 3d, 6d, and 9d KO2 sample lanes have been replaced with the corresponding lanes from the underlying blot (S1 File). The updated Fig 5E and underlying data (S1 File) show no signal in the 3d, 6d, and 9d KO2 sample lanes, supporting the results in the original figure.

In Fig 7C, the KO1 and KO2 lanes in the H3 and beta-tubulin panels are erroneous duplicates of the left-hand panel R1 and DO5 lanes in the H3 and beta-tubulin panels in Figure S2I. In Fig 7D the KO1 and KO2 lanes in the H3 panel are erroneous duplicates of the R1 and DO5 lanes in the H3 panel in the right-hand panel in Figure S2I. An updated Fig 7 is provided here in which the H3 and beta-tubulin panels in Fig 7C and 7D have been replaced with the correct western blot data from the original experiment. The full-length underlying blots supporting Fig 3 are in S2 and S3 Files. The underlying blots for panels contained in S2I are available in S4, S5 and S6 Files.

The underlying data for Figs 2B, 4H, S1A, S1B, S1C, S2D, S2J, and S4B are no longer available. All other data remain available upon request from the corresponding author as per the data availability policy in place at the time of this article’s publication.

The authors apologize for the errors in the published article.

**Fig 1:**
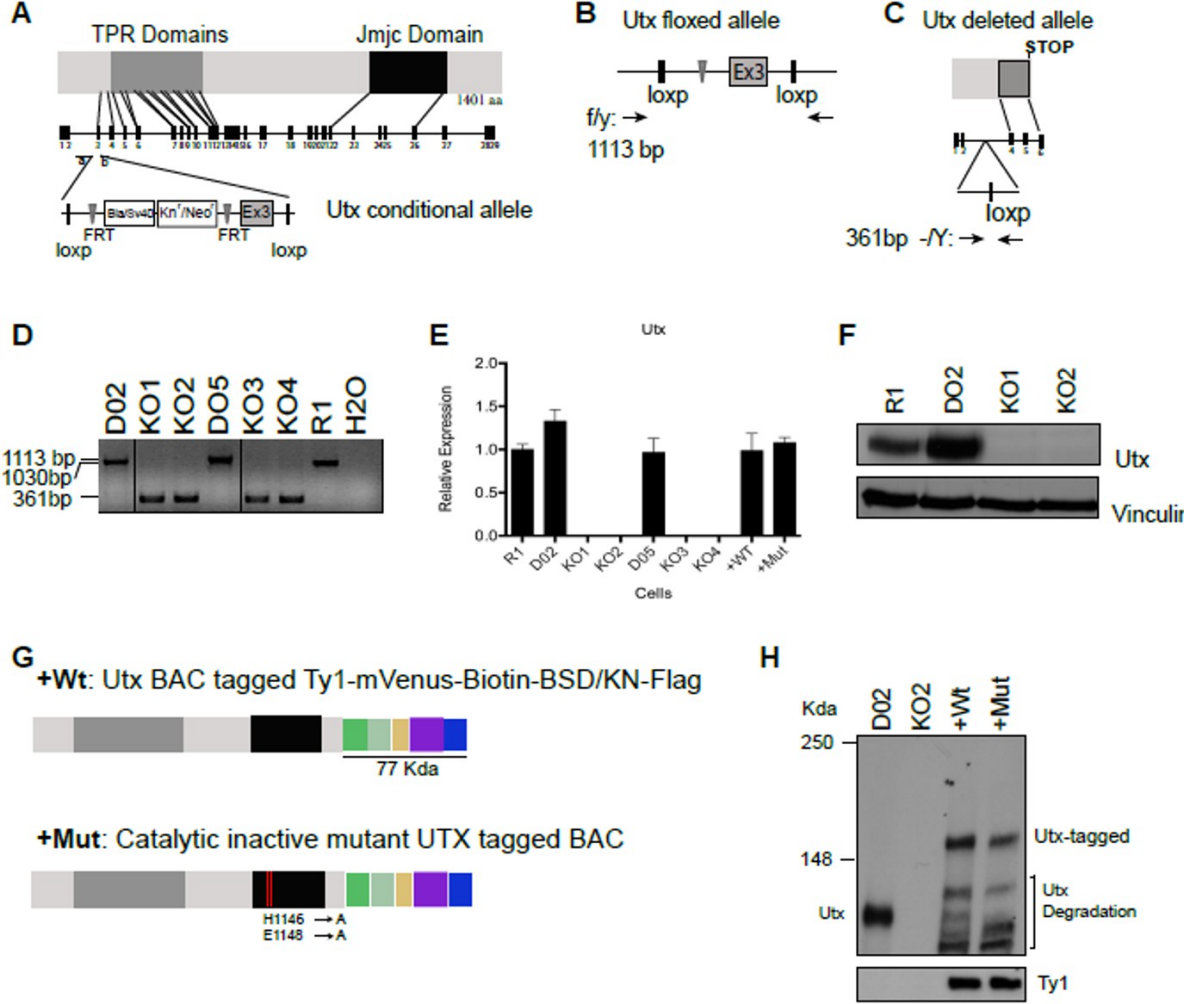
Generation of Utx knockout ESCs, and knockout ESCs complemented with wild type and catalytic mutant Utx. (A) Overview of the functional domains in Utx, the genomic locus of *Utx*, and the conditional targeting cassette for exon 3. (B) Predicted *Utx* floxed allele showing targeted cassette after treating targeted Utx clones with Flp recombinase. (C) Predicted *Utx* locus and deleted allele after treating with Cre recombinase. (D) Genotyping of *Utx* locus in ESCs after Flp and Cre recombination: agarose gel showing PCR amplification of wild type untargeted allele (R1:1030 bp), floxed allele (D02, D05:1113 bp) and deleted allele ESCs (KO Clones 1–4:361 bp). Black lines indicate where non-adjacent lanes from the same gel are spliced together. (E) *Utx* expression levels determined by quantitative RT-PCR analysis (normalized to *Rplp0* and the levels expressed in the R1 ESC line). (F) Western blot analysis of Utx expressed in WT, floxed, KO1 and KO2 ESCs. Vinculin served as a loading control. (G) Schematic representation of wild type and catalytic inactive mutant BAC proteins tagged with two copies of the Ty1 peptide, the Venus fluorescence protein, a biotin tag, two rox sites surrounding the coding regions of Blasticidin/Kanamycin resistance gene and three copies of the flag tag. (H) Immunoblot showing endogenous and tagged Utx levels and Ty1 expression in floxed (D02), knockout clone (KO2), wild type Utx BAC (+WT) and catalytic mutant Utx BAC (+Mut)].

**Fig 5:**
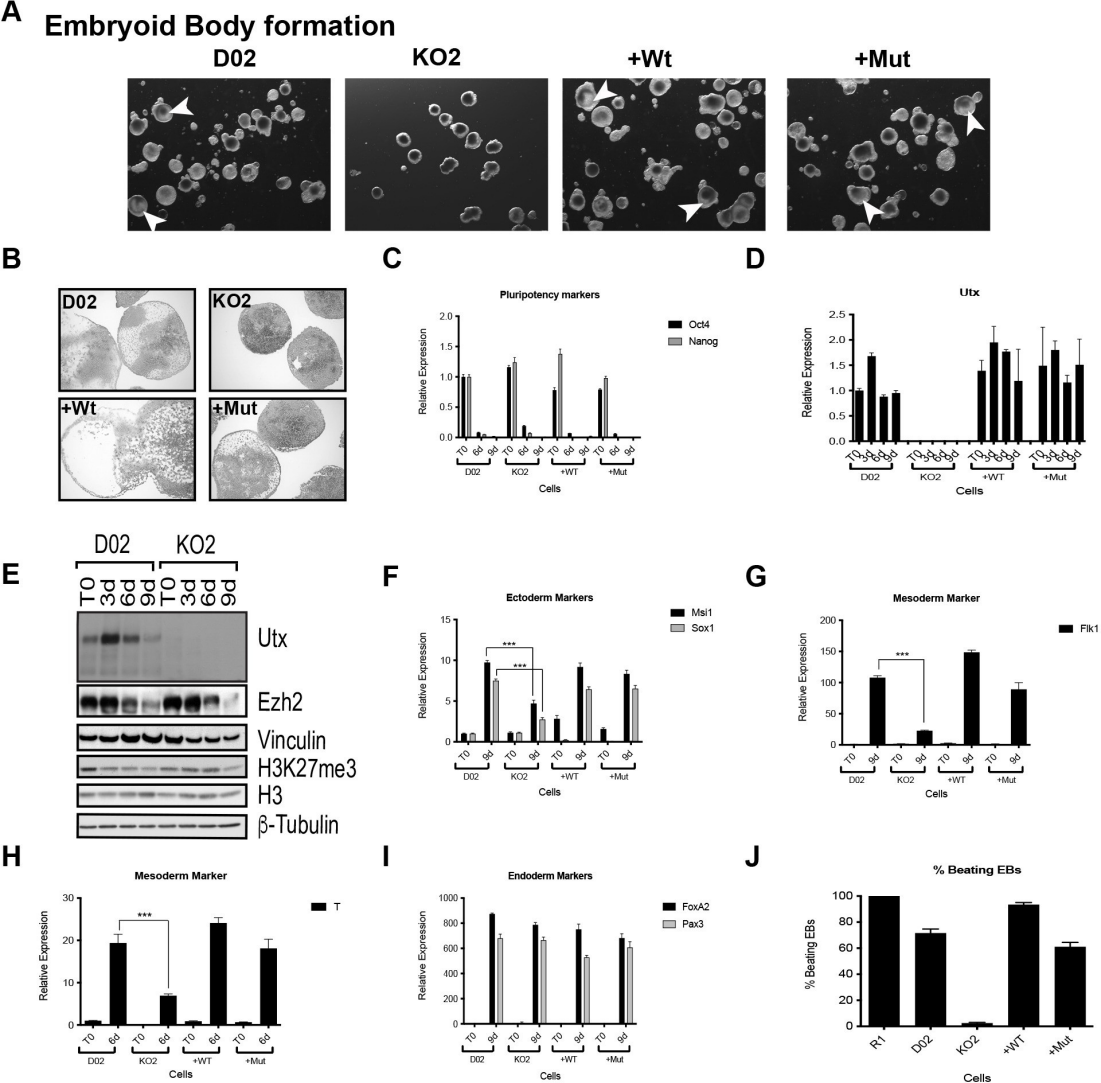
Utx is required for proper differentiation of ESCs. (A) Morphology of embryoid bodies 9 days after formation using the indicated ESCs as a starting material. White arrowheads depict some internal cavitation. (B) H&E staining of EBs harvested at day 10 post differentiation. (C) Pluripotency markers Oct4 and Nanog expression after 6 and 9 days of differentiation. (D) Utx expression levels before and after 3, 6 and 9 days of EB differentiation. (E) Western blot for Utx, Ezh2 and H3K27me3 during EB formation of ESCs. Vinculin, H3 and ß-tubulin were used as loading controls. (F, G, I) Gene activation of ectodermal (Msi1, Sox1), mesodermal (Flk1) and endodermal (FoxA2, Pax3) markers after 9 days of EB differentiation. (H) Expression levels of mesodermal marker Brachyury after 6 days of differentiation. All RT-qPCRs were normalized to the expression in D02 at T0 and *Rplp0*. (J) R1, D02, KO2, +Wt and +Mut percentage of beating EBs after EB formation and cardiac lineage differentiation. Error bars represent SD, n  =  3 independent assays (***p<0.0005, two tailed Student’s test).

**Fig 7:**
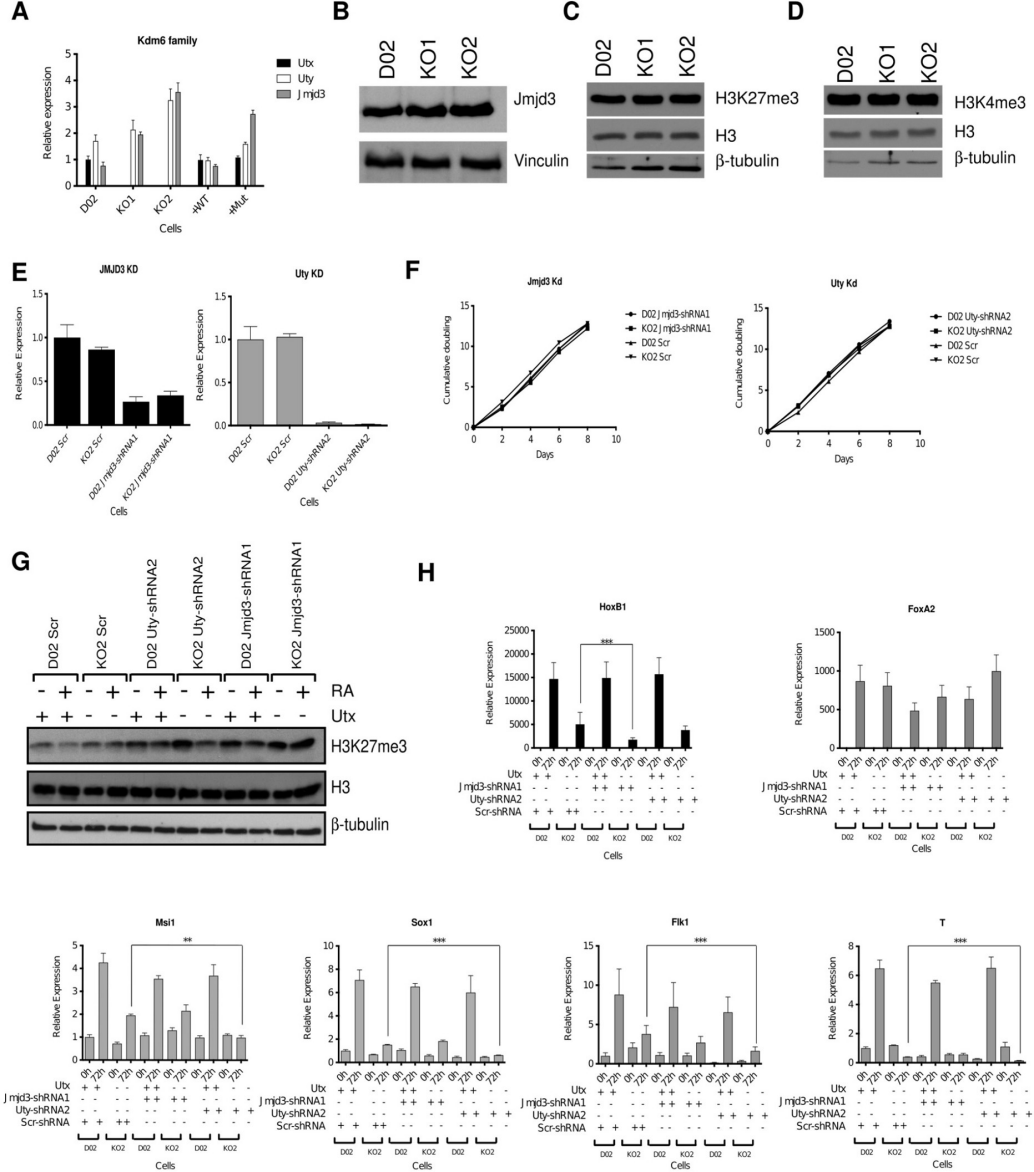
Jmjd3 and Uty contribute to the regulation of developmental genes during differentiation. (A) The expression levels of the Kdm6 family in ESCs measured by RT-qPCR and normalized to *Rplp0* and D02. (B) Expression levels of Jmjd3 in D02, KO1, and KO2 ESCs. Vinculin was used as loading control. (C–D) Western blots showing the H3K27me3 and H3K4me3 levels in the indicated cell lines. ß-tubulin and H3 were used as loading controls. (E) The efficiency of Jmjd3-shRNA1 and Uty-shRNA2 knockdown in the indicated cell lines as measured by RT-qPCR and normalized to *Rplp0*. (F) Cell proliferation analysis of the indicated cells lines. (G) Western blot showing H3K27me3 levels in the indicated cell lines before and after 72h of RA differentiation (H) mRNA expression levels of Utx target genes in Utx knockout (KO2) cells with and without knocking down Jmjd3 or Uty knockdown cells. All RT-qPCRs were normalized to *Rplp0* and the expression levels in D02 Scr at T0. Error bars represent SD, n  =  3 independent assays (**p<0.005; ***p<0.0005, two tailed Student’s test).

## Supporting information

S1 File Underlying data for Figures 1 and 5. (ZIP)

S2 File Underlying data for Figure 7, part 1. (ZIP)

S3 File Underlying data for Figure 7, part 2. (ZIP)

S4 File Underlying data for Figure S2, part 1. (ZIP)

S5 File Underlying data for Figure S2, part 2. (ZIP)

S6 File Underlying data for Figure S2, part 3. (ZIP)
