# Supplementary figures and images for: Utx Is Required for Proper Induction of Ectoderm and Mesoderm during Differentiation of Embryonic Stem Cells
Source: PLoS One. 2013 Apr 3;8(4):e60020. doi: 10.1371/journal.pone.0060020 (PMC3616089; doi:10.1371/journal.pone.0060020)

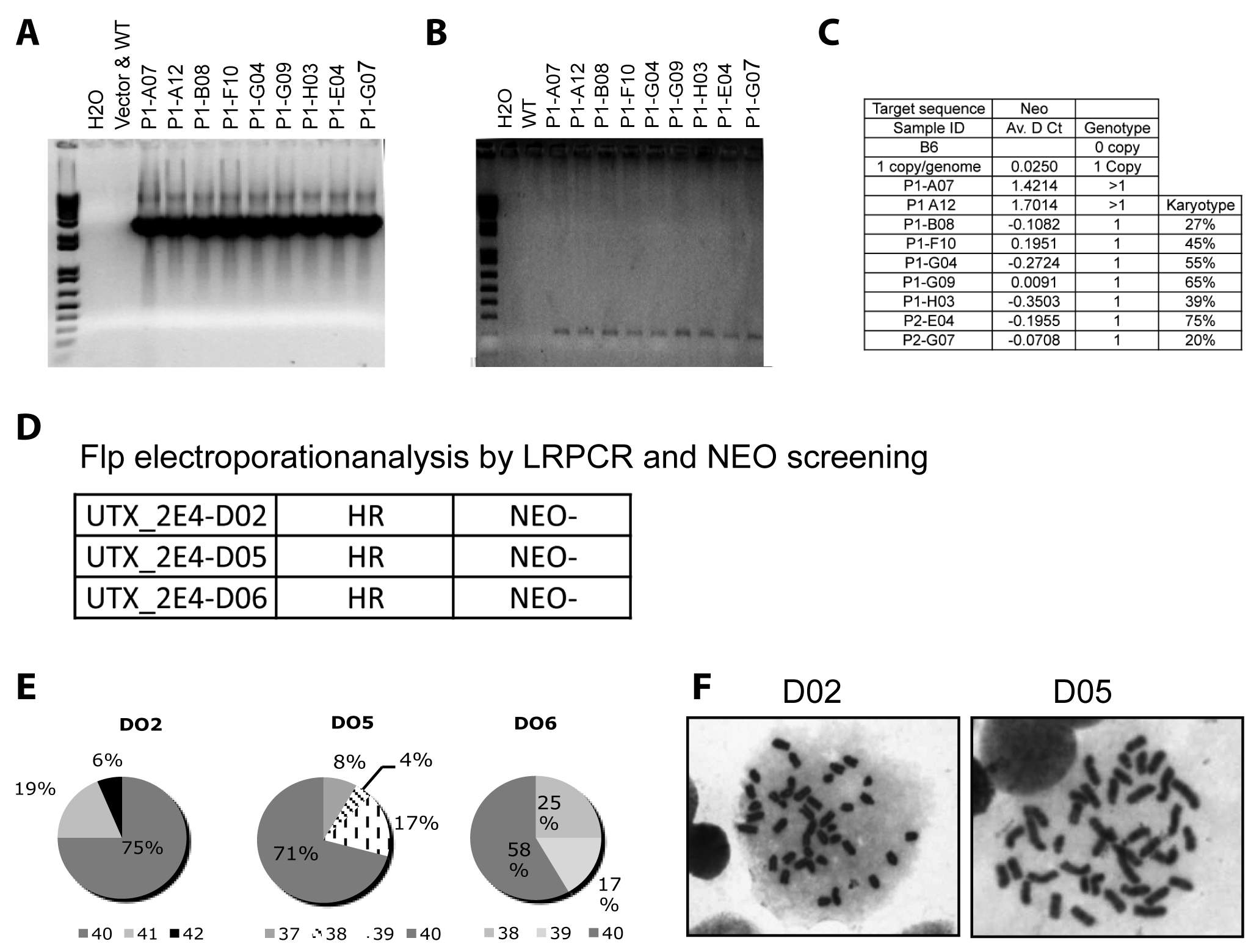

Supplement: Figure S1 — The generation of Utx knockout ESCs. (A) DNA long-range PCR analysis of selected ESC clones after the electroporation of the Utx targeting construct. The primers were designed to amplify a 3 kb fragment. One primer was located outside of the homology arm of the targeting construct, and the other one inside the targeting construct covering the closest loxp site, Vector and wild type R1 DNA were used as negative controls. (B) Agarose gel showing PCR amplification of a 215 bp band with primers designed to amplify the other loxP site, ensuring its integrity after homologous recombination. R1 wild type DNA was used as a negative control. (C) Analysis of a qPCR designed to amplify the Neomycin cassette at the targeted allele to ensure the integration of a unique copy of the construct at the ESCs genome. Karyotype analyses of the targeted ESCs guide us to select P1-E04 to proceed with the study. (D) Table collecting results from the analysis by long range PCR of three floxed clones (D02, D05, D06) obtained after flp recombinase electroporation and selection. Correct excision of Kanamycin-Neomycin resistance cassette is designated as HR and the absence of the neomycin-encoding gene as Neo-. (E) Graphics showing the karyotype analysis of the three floxed clones. Karyotype analyses were used to choose clones D02 and D05 (with 75% and 71% of metaphases showing 40 chromosomes, respectively) for further studies. (F) Representative metaphases for clone D02 and D05. (TIF) [file pone.0060020.s001.tif]

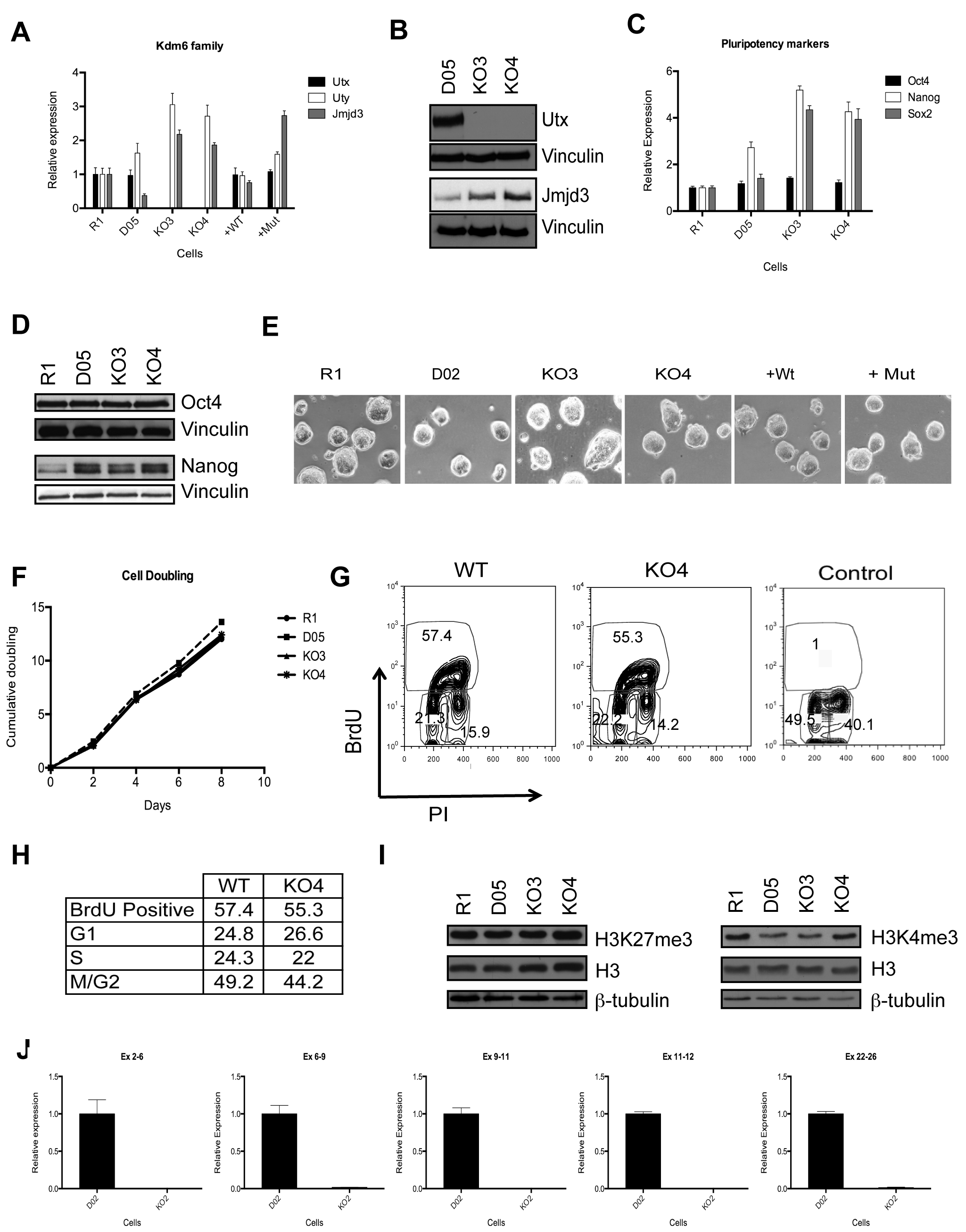

Supplement: Figure S2 — Characterization of Utx KO clones. (A) The expression of Kdm6 family members was determined by quantitative RT-PCR analysis (normalized to Rplp0 and to the expression in R1 ESCs at T0). (B) Utx and Jmjd3 protein levels in floxed and in KO clones 3 and 4 ESCs. Vinculin was used as loading control. (C) mRNA expression levels of the pluripotency markers Oct4, Nanog and Sox2 in the indicated clones. (D) Western blots showing Oct4 and Nanog levels in the indicated clones. Vinculin was used as loading control. (E) Morphology of ESCs in the indicated clones. (F) Cell proliferation assay of WT, floxed and KO ESCs cell at the indicated days after plating. (G) Flow cytometry analysis of WT (D02) and KO4 pulsed with BrdU and stained with anti-BrdU antibody. Control represents cells without BrdU pulsing. (H) Cell cycle analysis by flow cytometry of WT and KO clone stained with PI (propidium iodine). (I) Western blot analysis showing the levels of H3K27me3 and H3K4me3 in the indicated cell lines. ß-tubulin and H3 were used as loading controls (I) Utx expression levels in control D02 and KO2 ESCs determined by quantitative RT-PCR analysis (normalized to Rplp0 levels). Primers were designed to cover exons encoding the following functional domains: TPR 1–2 (E3–6), TPR 3–4 (E6–9), TPR 5–6 (E 9–11), TPR 7–8 (E 11–12), and JmjC (E22–26). (TIF) [file pone.0060020.s002.tif]

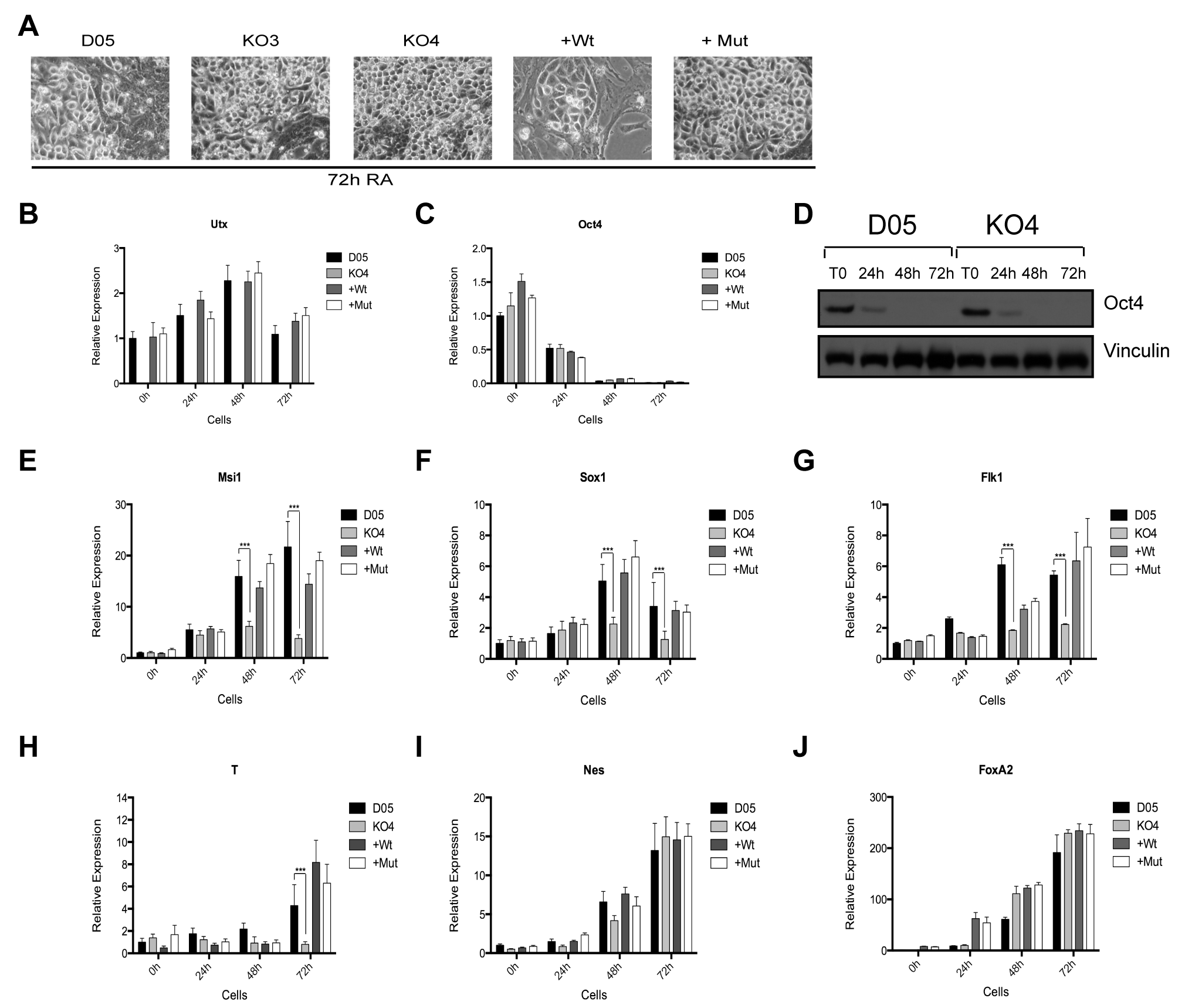

Supplement: Figure S3 — Utx is required for normal ESC differentiation. Time points were taken 24, 48 and 72 hours after treatment with 1 µM RA and analyzed by Western blotting or RT-qPCR. All qPCRs were normalized to Rplp0 and the level in D05 ESCs at T0. (A) The morphology of the indicated ESCs 72 h after addition of retinoic acid. (B) The levels of Utx mRNA expression during differentiation. (C, D) The expression of Oct 4 mRNA (C) and proteins levels (D) during RA-induced differentiation in the indicated cell lines. Vinculin was used as loading control. (E–J) mRNA expression analysis of the indicated genes during monolayer differentiation of control (D05) and Utx KO cells. Utx knockout cells transfected with wild type (+Wt) or catalytic mutant (+Mut) Utx BAC were also analyzed. Fold activation is presented for three ectodermal markers Msi1, Sox1, Nes; two mesodermal markers Flk1, T and one endoderm marker FoxA2. Error bars represent SD, n = 3 independent assays (***p<0.0005, two tailed Student’s test). (TIF) [file pone.0060020.s003.tif]

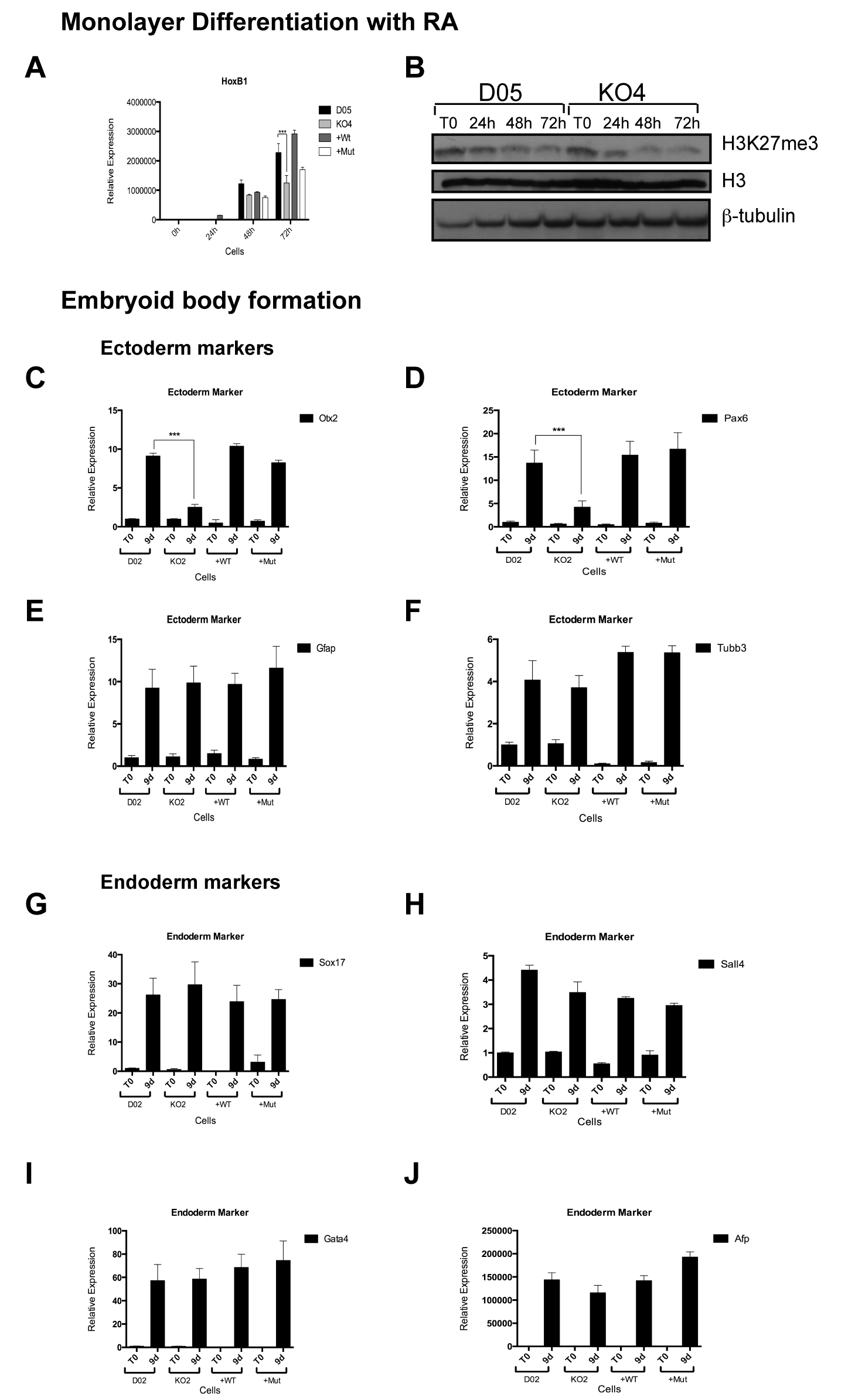

Supplement: Figure S4 — Utx is important for normal ESC differentiation. Monolayer differentiation: Time points were taken 24, 48 and 72 hours after treatment with 1 µM RA. Embryoid bodies formation: samples were taken before and after 3, 6 and 9 days of EB differentiation. Samples were analyzed by Western blotting or RT-qPCR. All qPCRs were normalized to Rplp0 and the level in D02 or D05 ESCs at T0. (A) mRNA expression analysis of Hoxb1 during RA-induced differentiation in D05, K04 ESCs. Utx knockout cells transfected with Wt (+Wt) or catalytic mutant (+Mut) Utx BAC were also analyzed. (B) Western blot of H3K27me3 levels in the indicated cell lines during differentiation. ß-tubulin and H3 were used as loading controls. (C–F) Expression levels of ectoderm markers Otx2, Pax6, Gfap and Tubb3 in the indicated cells. (G–J) mRNA analysis of endoderm markers Sox17, Sall4, Gata4 and Afp in D02, KO2, +Wt and +Mut ESCs. Error bars represent SD, n = 3 independent assays (***p<0.0005, two tailed Student’s test). (TIF) [file pone.0060020.s004.tif]

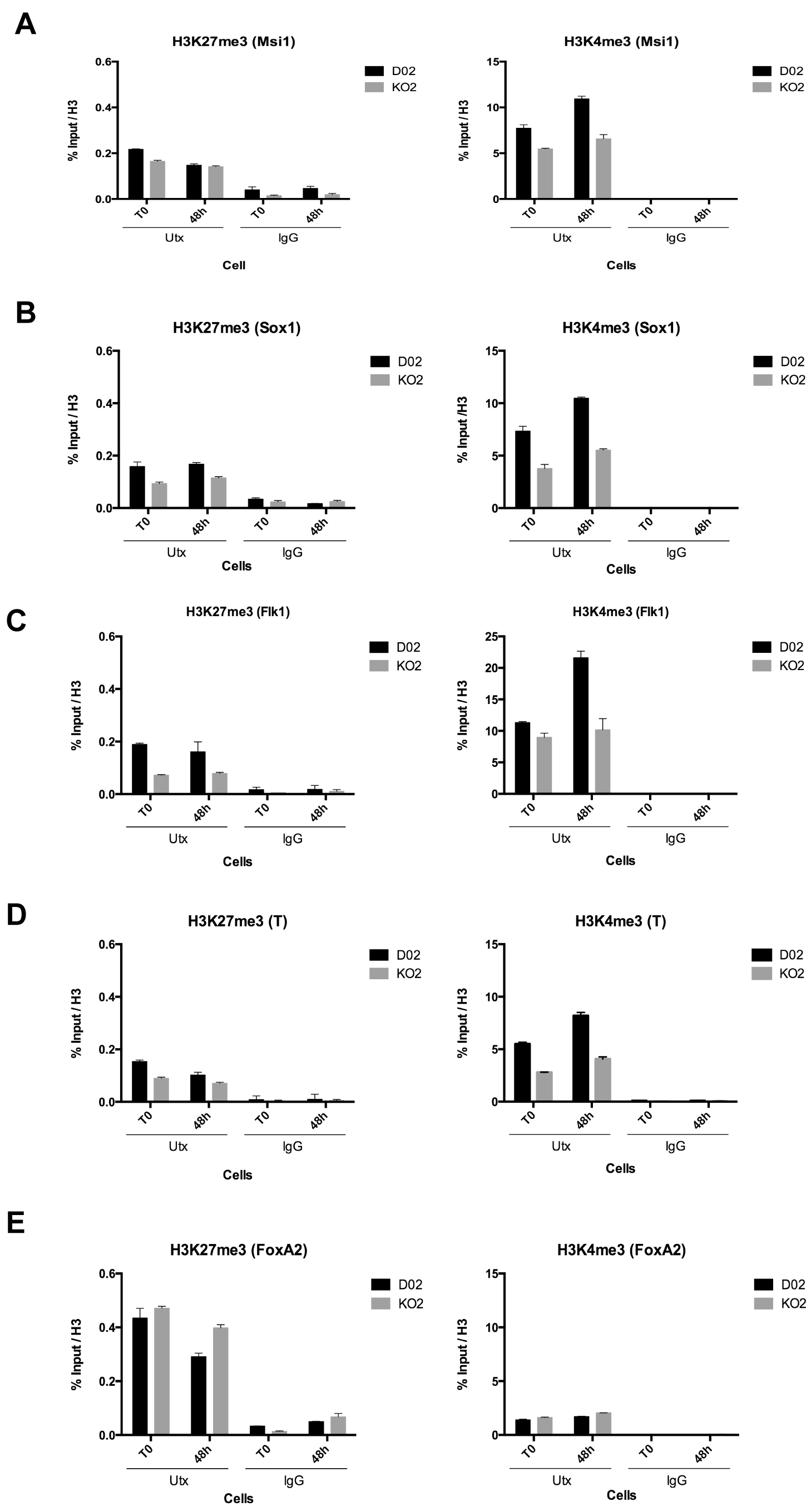

Supplement: Figure S5 — H3K27me3 and H3K4me3 levels at the promoter regions of developmental genes. (A–E) ChIP assays of the indicated histone modifications on the ectoderm (Msi1, Sox1); mesoderm (Flk1, T) and endoderm (FoxA2) promoters at the indicated times during differentiation. “% Input” represents (bound/input material x 100). Error bars represent SD, n = 3 independent assays. (TIF) [file pone.0060020.s005.tif]

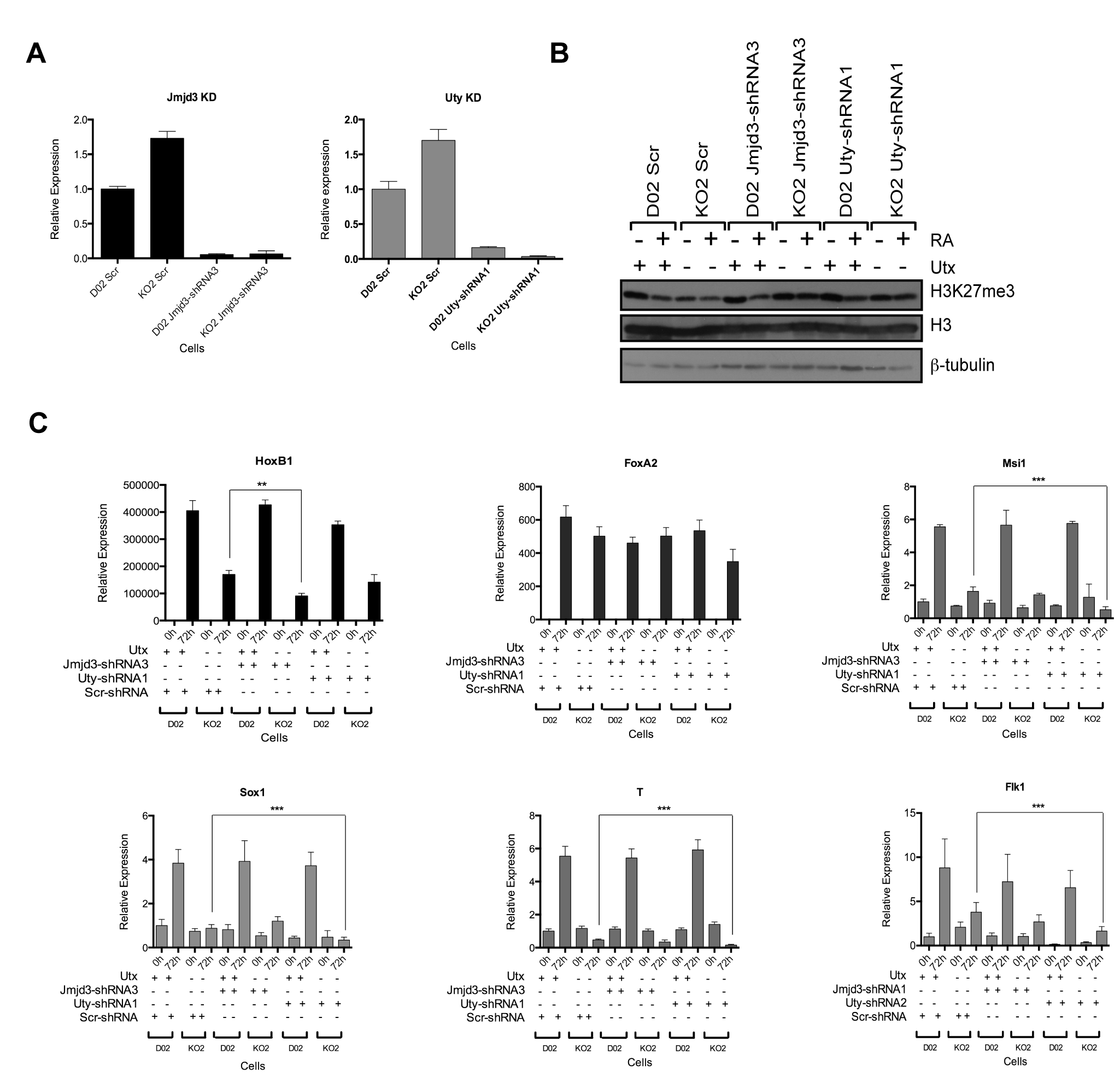

Supplement: Figure S6 — Overlapping functions of Utx, Jmjd3 and Uty. (A) Levels of Jmjd3 and Uty after in the indicated ESCs expressing Jmjd3-shRNA3 and Uty-shRNA1 as measured by RT-qPCR and normalized to Rplp0. (B) Western blot showing H3K27m3 levels in the indicated cell lines before and after 72 h of RA differentiation. (C) mRNA expression levels of Utx target genes in Utx knockout (KO2) cells with and without knocking down Jmjd3 or Uty. All RT-qPCRs were normalized to Rplp0 and the expression levels in D02 Scr at T0. Error bars represent SD, n = 3 independent assays (**p<0.005; ***p<0.0005, two tailed Student’s test). (TIF) [file pone.0060020.s006.tif]
