# Supplementary material for: Correction: Utx Is Required for Proper Induction of Ectoderm and Mesoderm during Differentiation of Embryonic Stem Cells
Source: PLoS One. 2024 Jun 27;19(6):e0306360. doi: 10.1371/journal.pone.0306360 (PMC11210840; doi:10.1371/journal.pone.0306360)
Supplement: S3 File — (ZIP) [file pone.0306360.s003.zip › S3 File/Figure 7G/Figure 7G H3K27me3_H3_b tubulin.pdf]

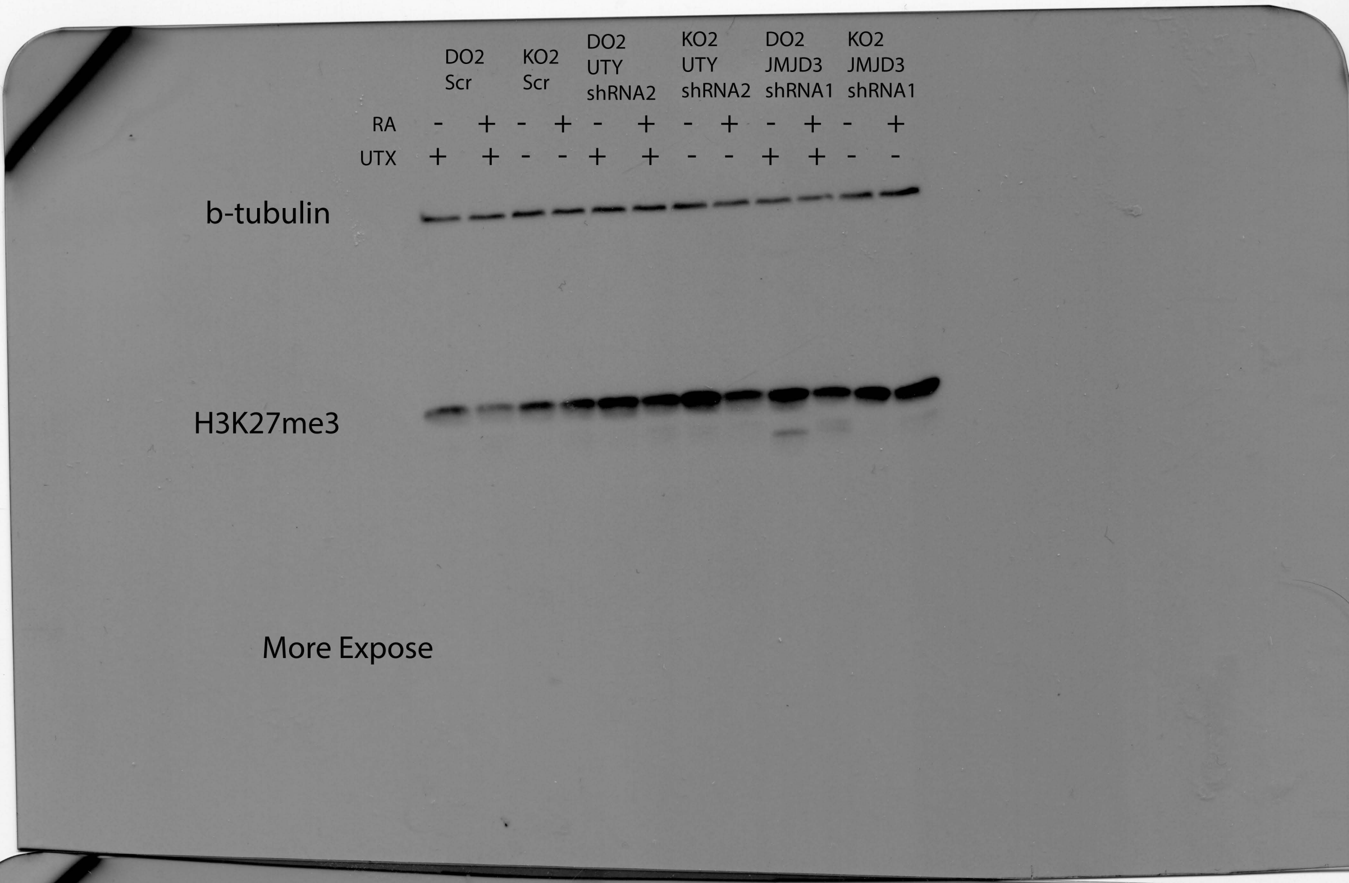
DO2 Scr
KO2 Scr
DO2 UTY shRNA2
KO2 UTY shRNA2
DO2 JMJD3 shRNA1
KO2 JMJD3 shRNA1
RA - + - + - + - + - + - +
UTX + + - - + + - - + + - -
b-tubulin
H3K27me3
More Expose

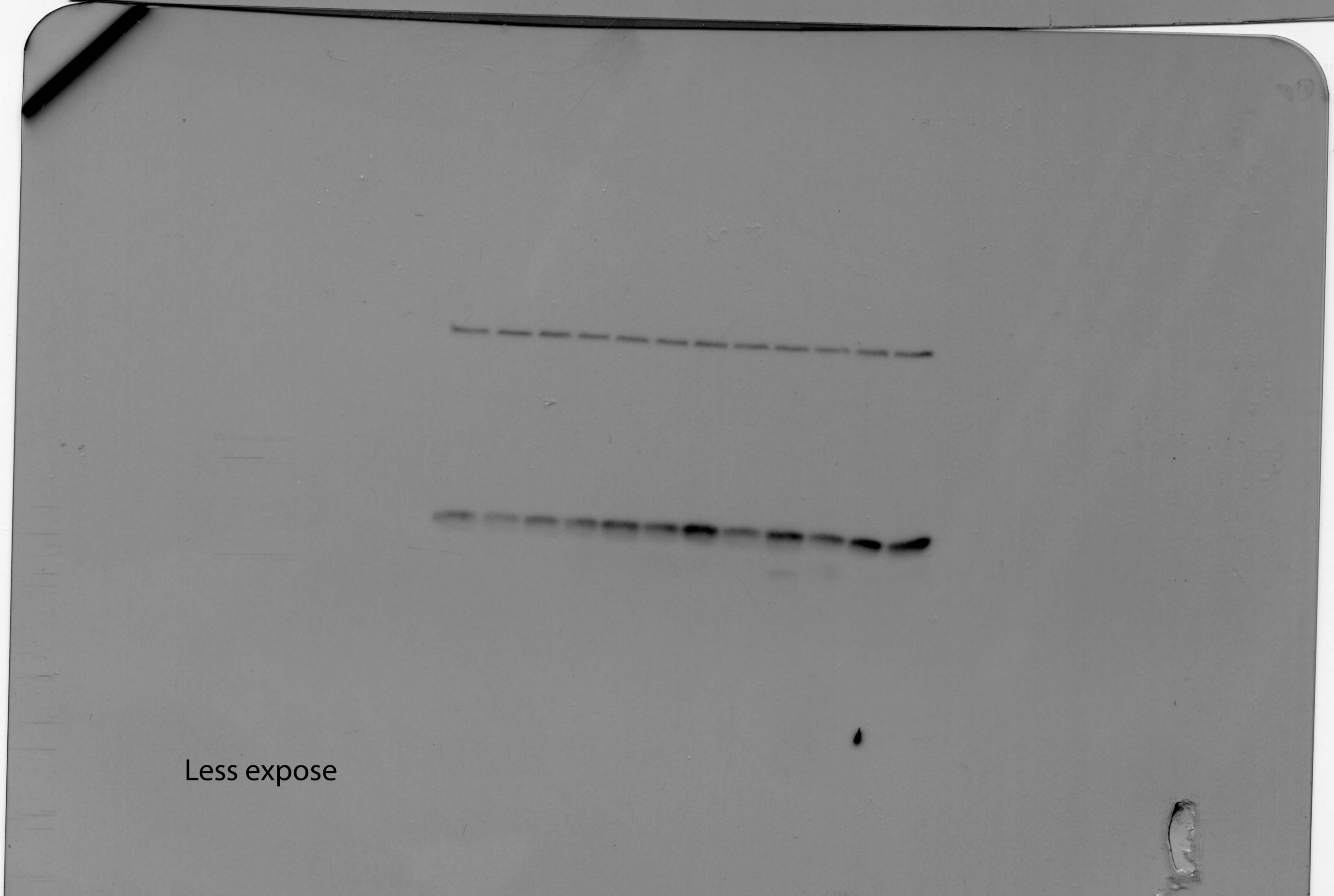
Less expose

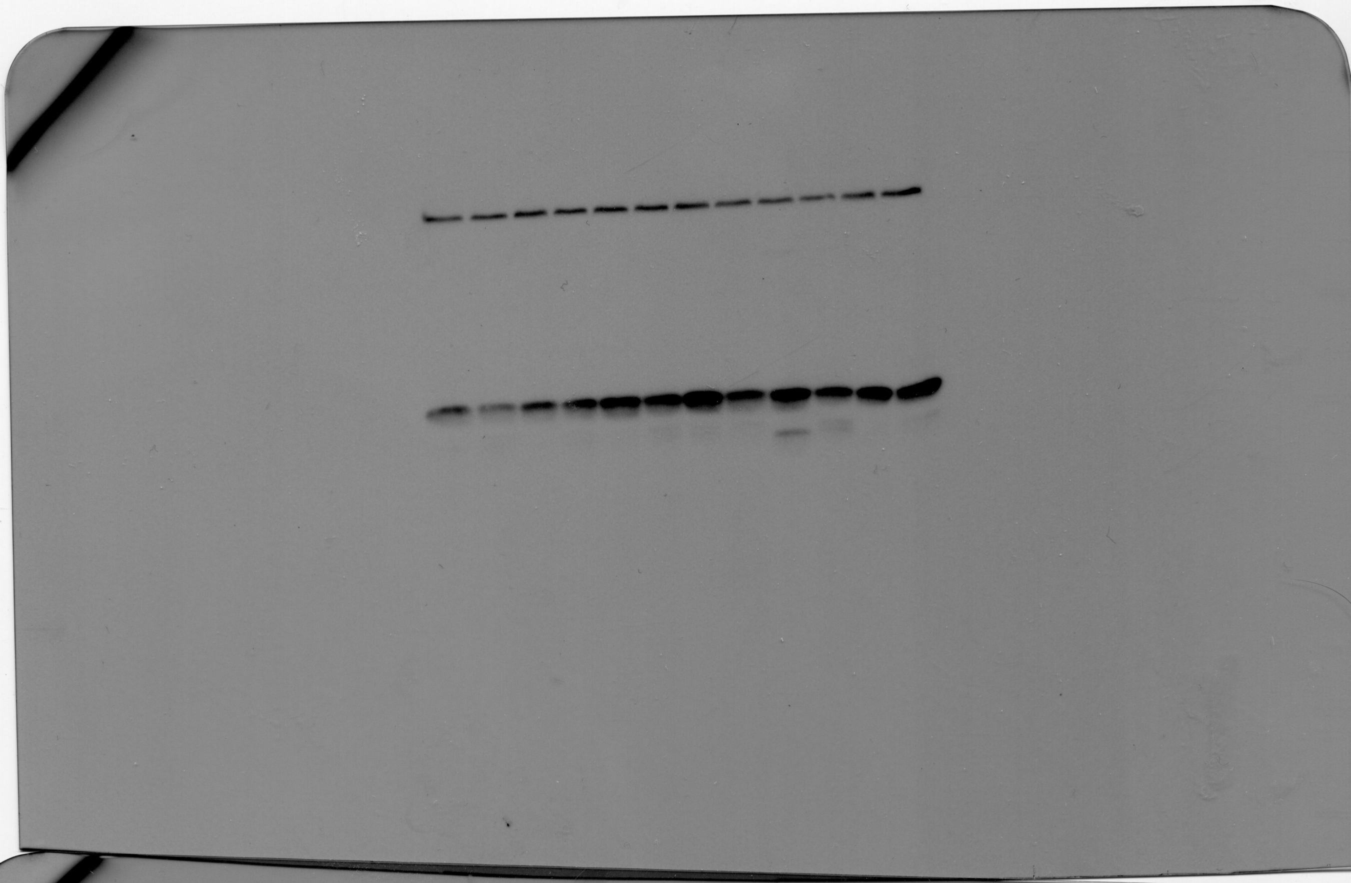

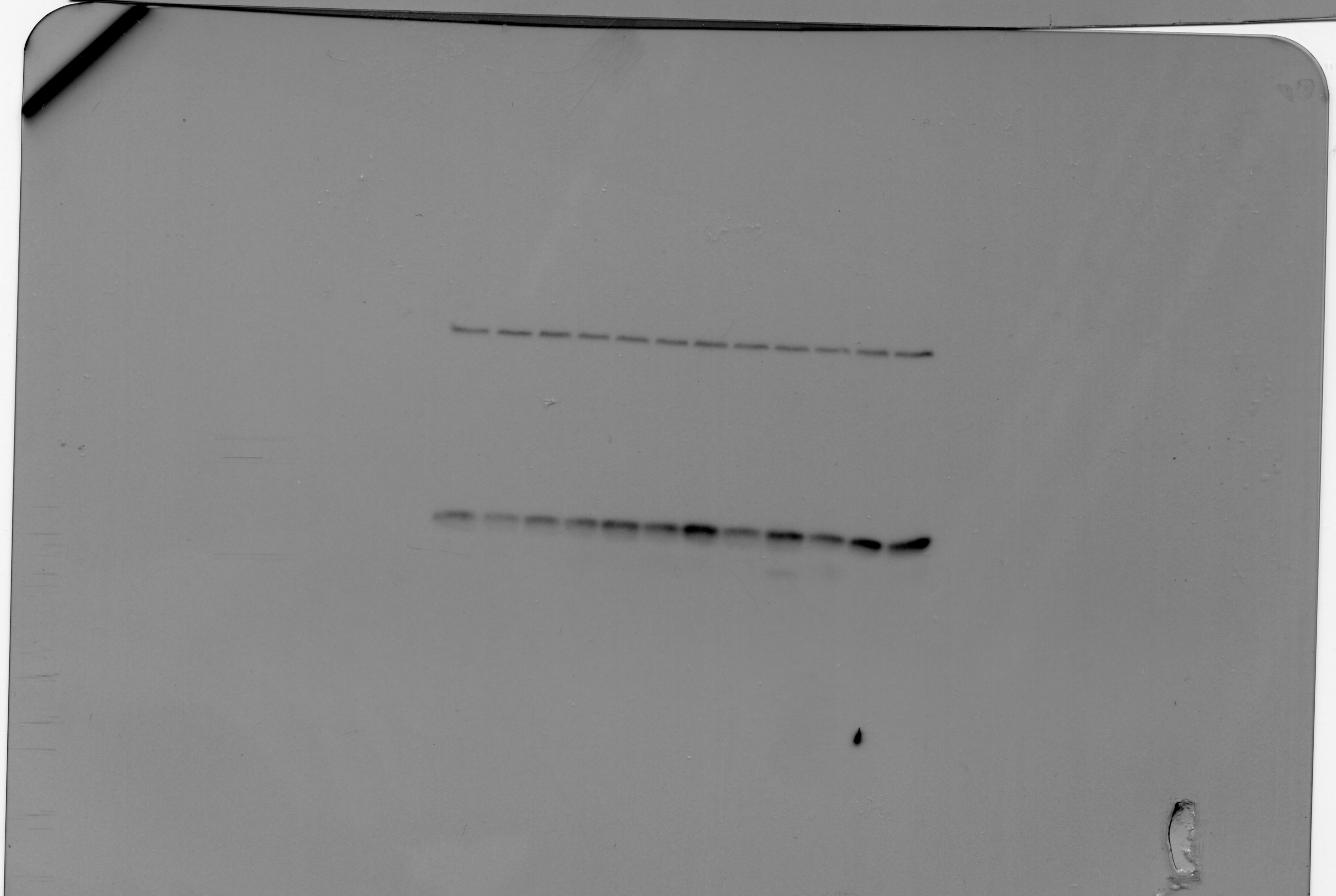

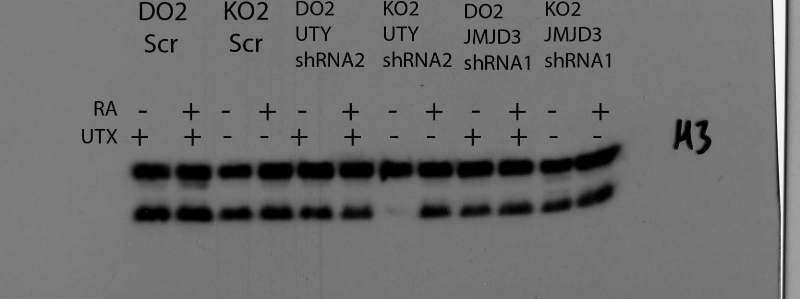
DO2 Scr
KO2 Scr
DO2 UTY shRNA2
KO2 UTY shRNA2
DO2 JMJD3 shRNA1
KO2 JMJD3 shRNA1
RA - + - + - + - + - + - +
UTX + + - - + + - - + + - -
H3

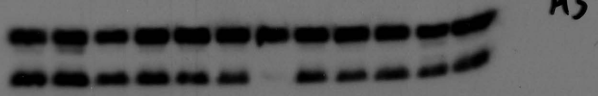
H3
