# Supplementary figures and images for: Correction: Utx Is Required for Proper Induction of Ectoderm and Mesoderm during Differentiation of Embryonic Stem Cells
Source: PLoS One. 2024 Jun 27;19(6):e0306360. doi: 10.1371/journal.pone.0306360 (PMC11210840; doi:10.1371/journal.pone.0306360)

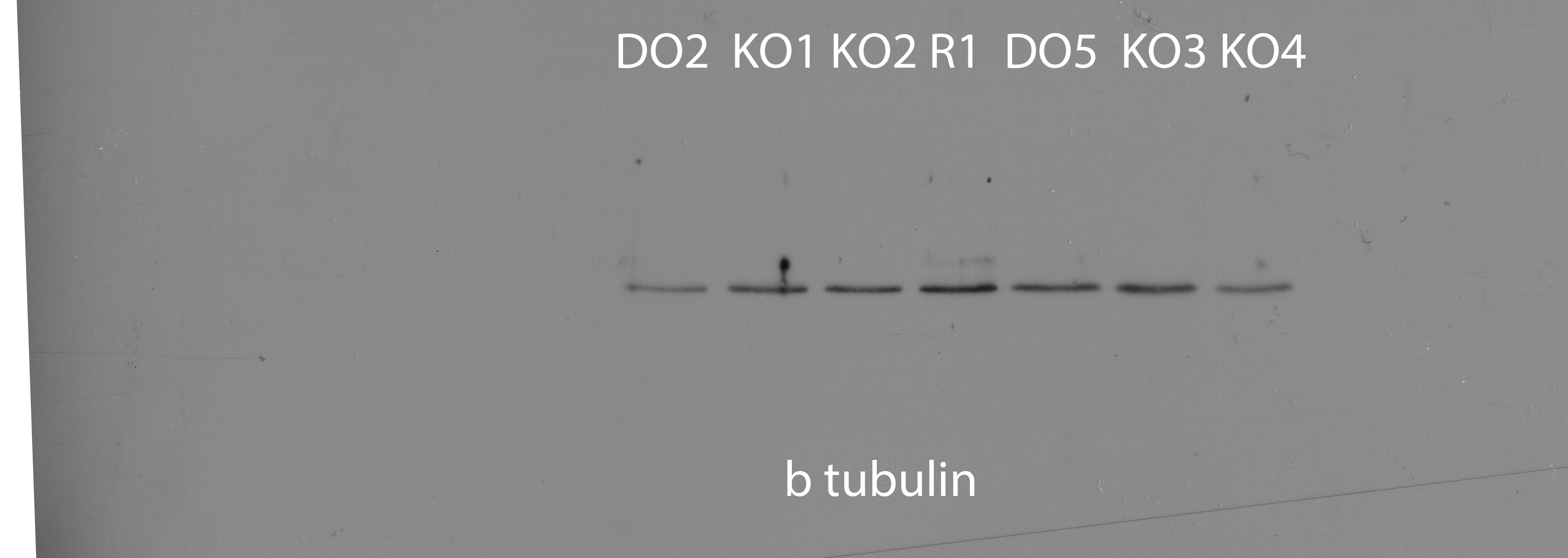

Supplement: S3 File — (ZIP) [file pone.0306360.s003.zip › S3 File/Figure 7D/Figure 7D and Figure S2I right panel b tubulin control Labelled.tif]

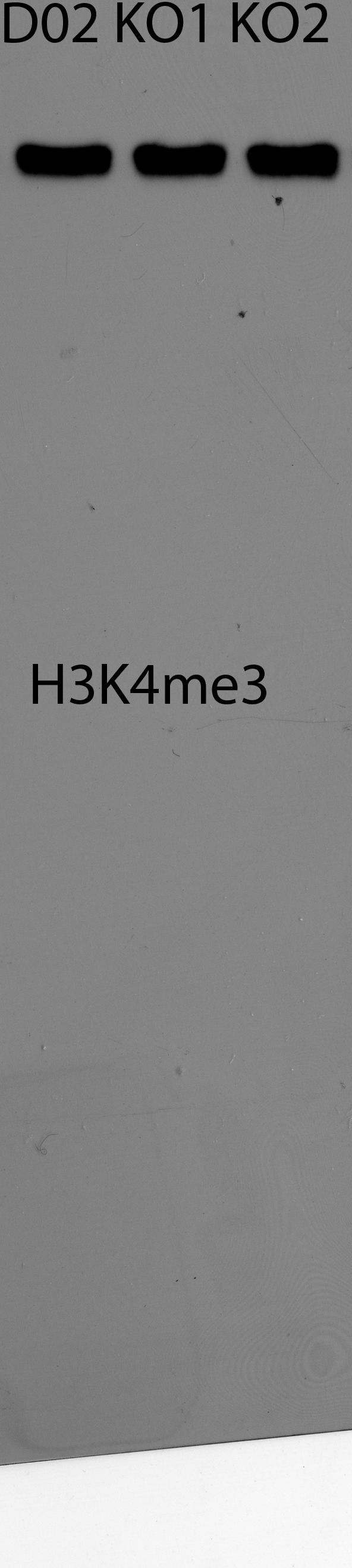

Supplement: S3 File — (ZIP) [file pone.0306360.s003.zip › S3 File/Figure 7D/Figure 7D H3K4me3 labelled.tif]

D02 KO1 KO2

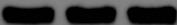

H3K4me3



DO2 KO1 KO2 R1 DO5 KO3 KO4

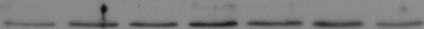

b tubulin

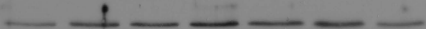

DO2 KO1 KO2 R1 DO5 KO3 KO4

H3 of H3K4me3

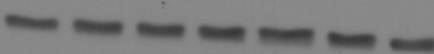

H3 of H3K4me3

Supplement: S3 File — (ZIP) [file pone.0306360.s003.zip › S3 File/Figure 7D/Figure 7D_H3K4me3_H3_B tubulin.pdf]

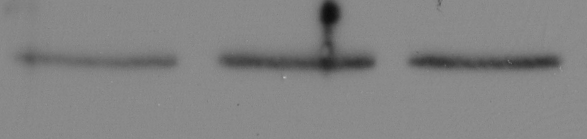

Supplement: S3 File — (ZIP) [file pone.0306360.s003.zip › S3 File/Figure 7D/New b tubulin.tif]

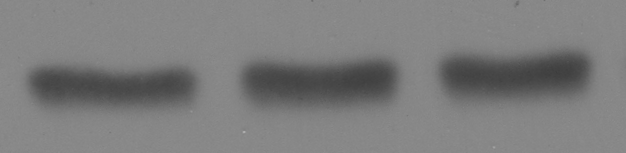

Supplement: S3 File — (ZIP) [file pone.0306360.s003.zip › S3 File/Figure 7D/New H3.tif]

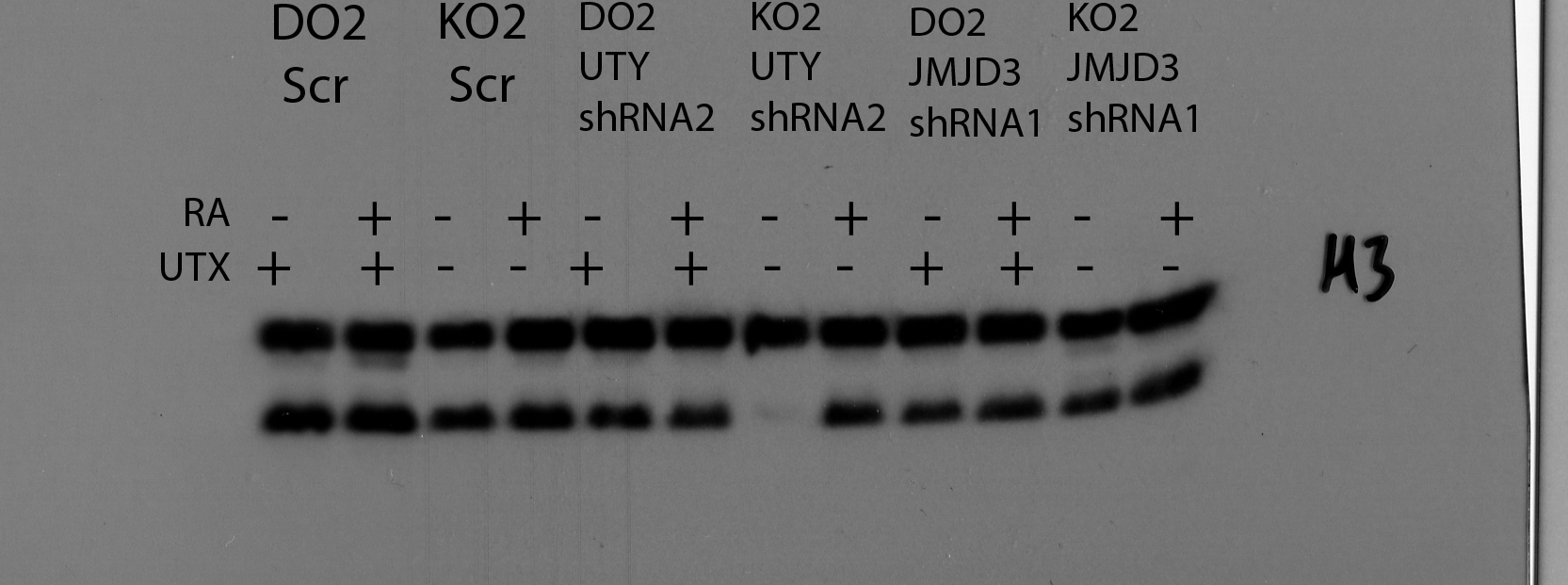

Supplement: S3 File — (ZIP) [file pone.0306360.s003.zip › S3 File/Figure 7G/Figure 7G_H3 control_labelled.tif]

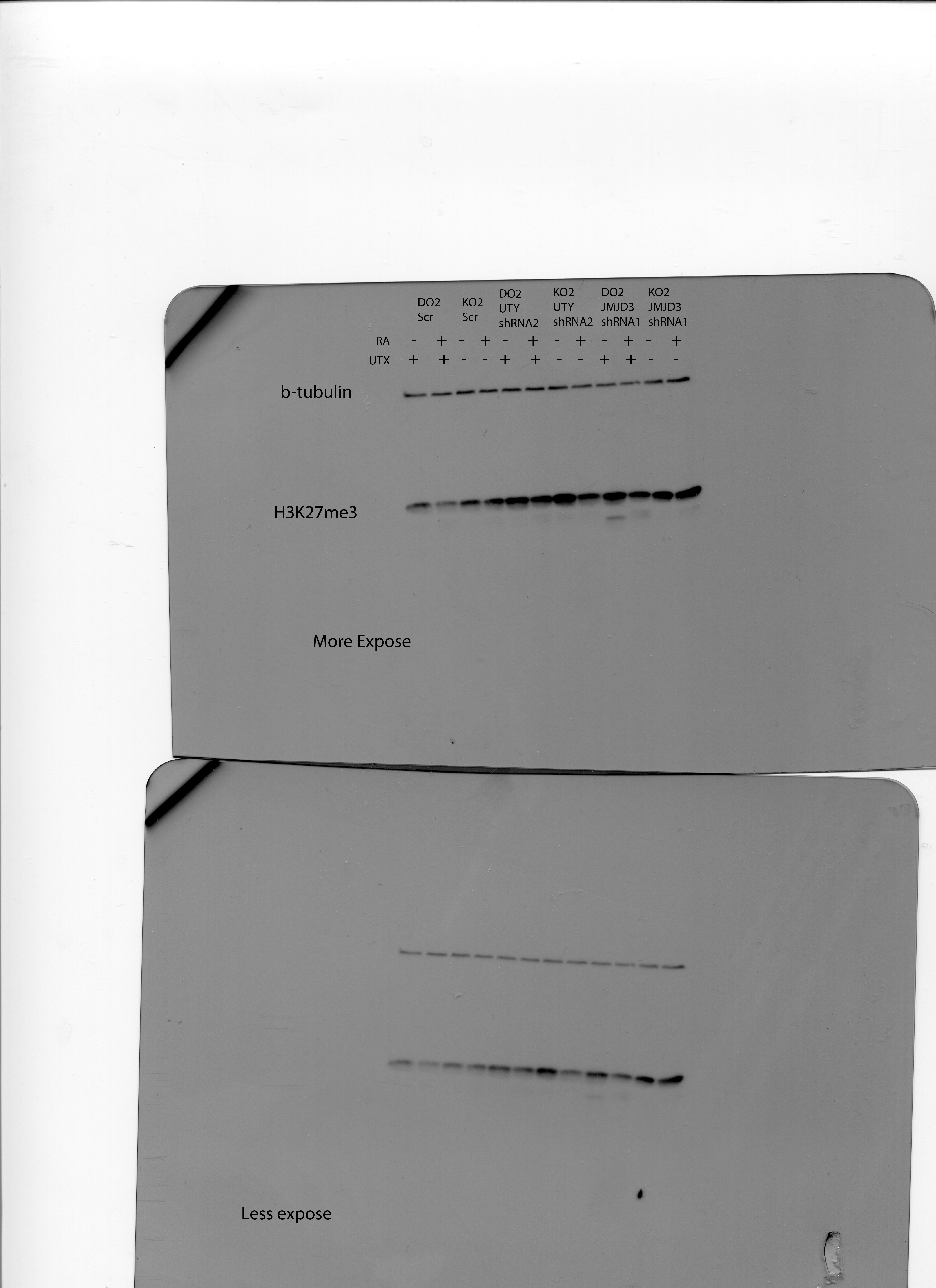

Supplement: S3 File — (ZIP) [file pone.0306360.s003.zip › S3 File/Figure 7G/Figure 7G_H3k27me3 and b tubulin control labelled.tif]

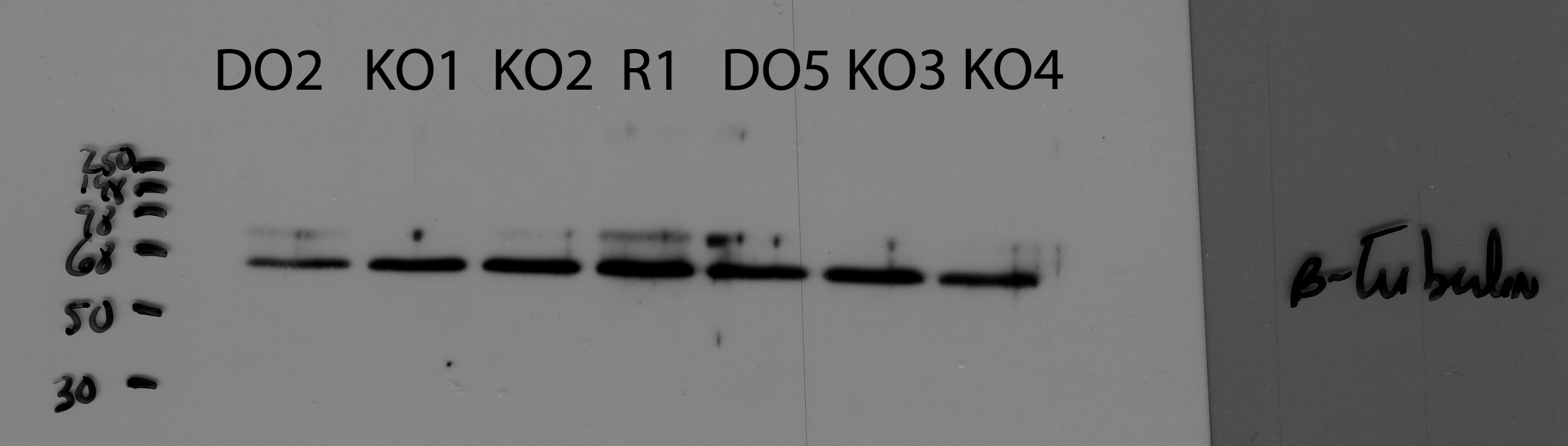

Supplement: S4 File — (ZIP) [file pone.0306360.s004.zip › File S4/Figure 7C and Figure S2i left panel b tubulin control labelled.tif]

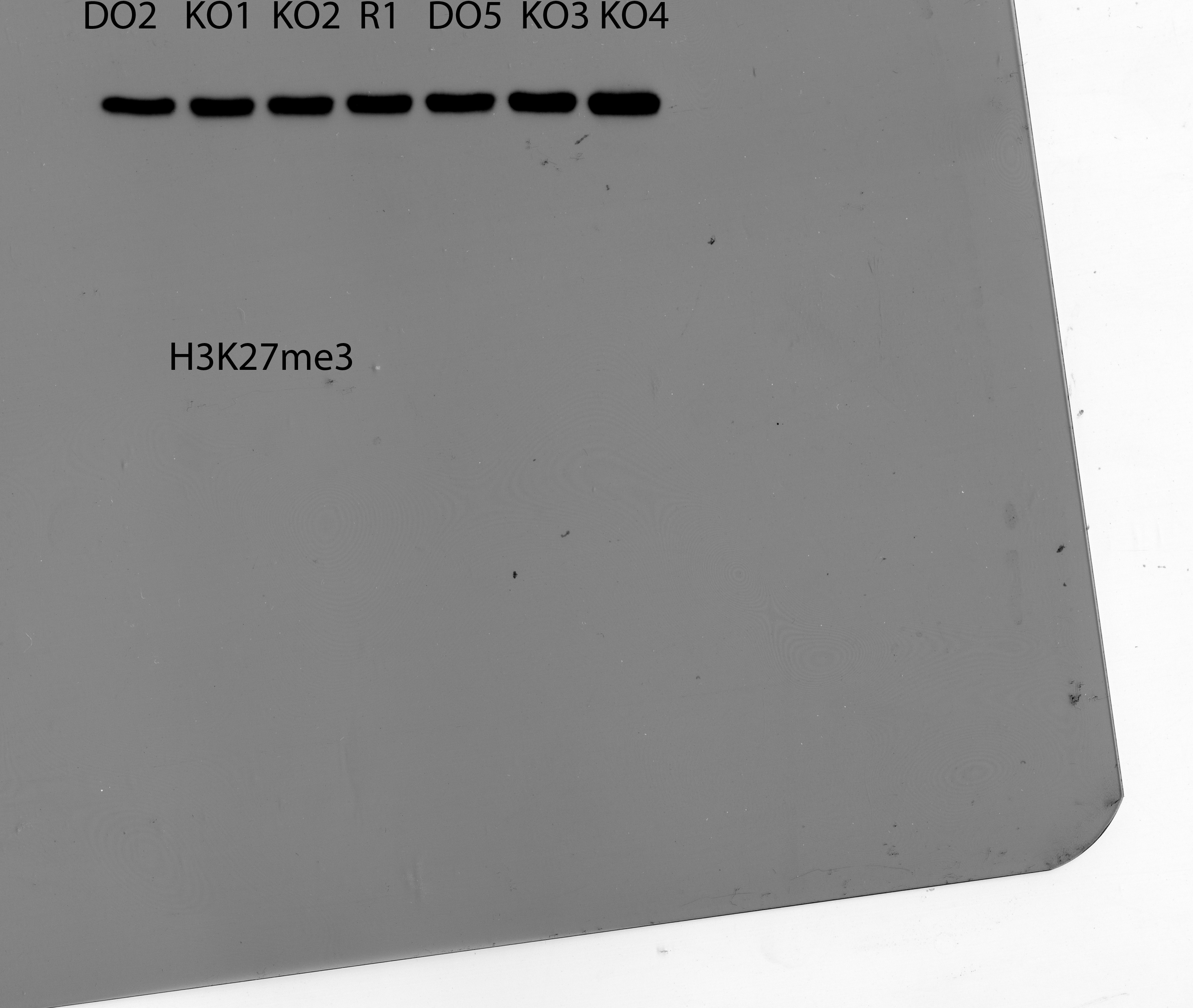

Supplement: S4 File — (ZIP) [file pone.0306360.s004.zip › File S4/Figure 7C and Figure S2I left panel H3K27 labelled.tif]

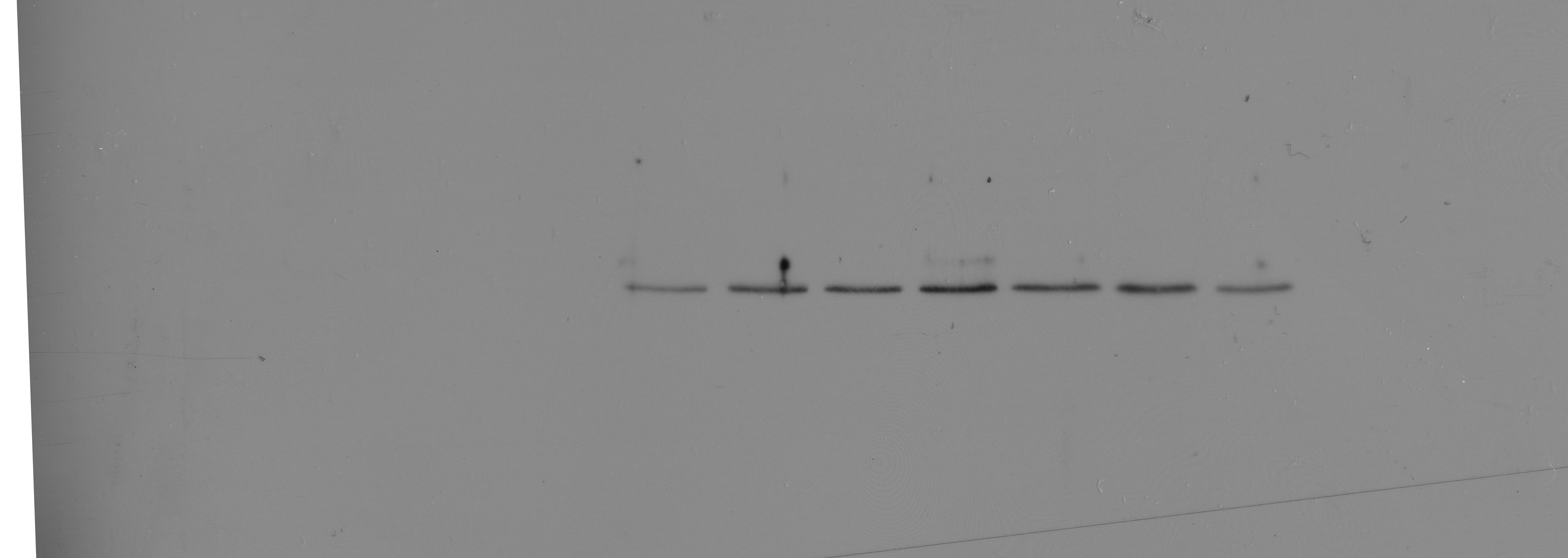

Supplement: S5 File — (ZIP) [file pone.0306360.s005.zip › File S5/Figure 7D and Figure S2I right pane b tubulin control.tif]

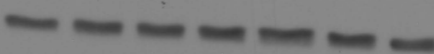

H3 of H3K4me3

R1 DO5 KO3 KO4

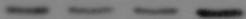

H3K4me3

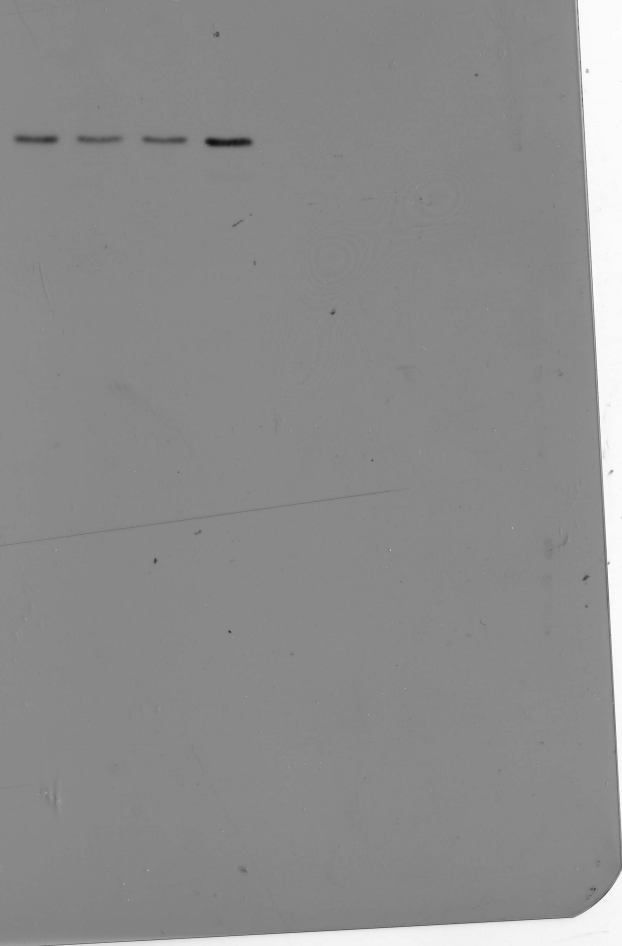

DO2 KO1 KO2 R1 DO5 KO3 KO4

H3 of H3K4me3

DO2 KO1 KO2 R1 DO5 KO3 KO4

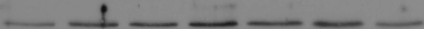

b tubulin

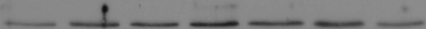

Supplement: S6 File — (ZIP) [file pone.0306360.s006.zip › File S6/Figure S2I Right Panel H3K4me3 H3 B tubulina.pdf]

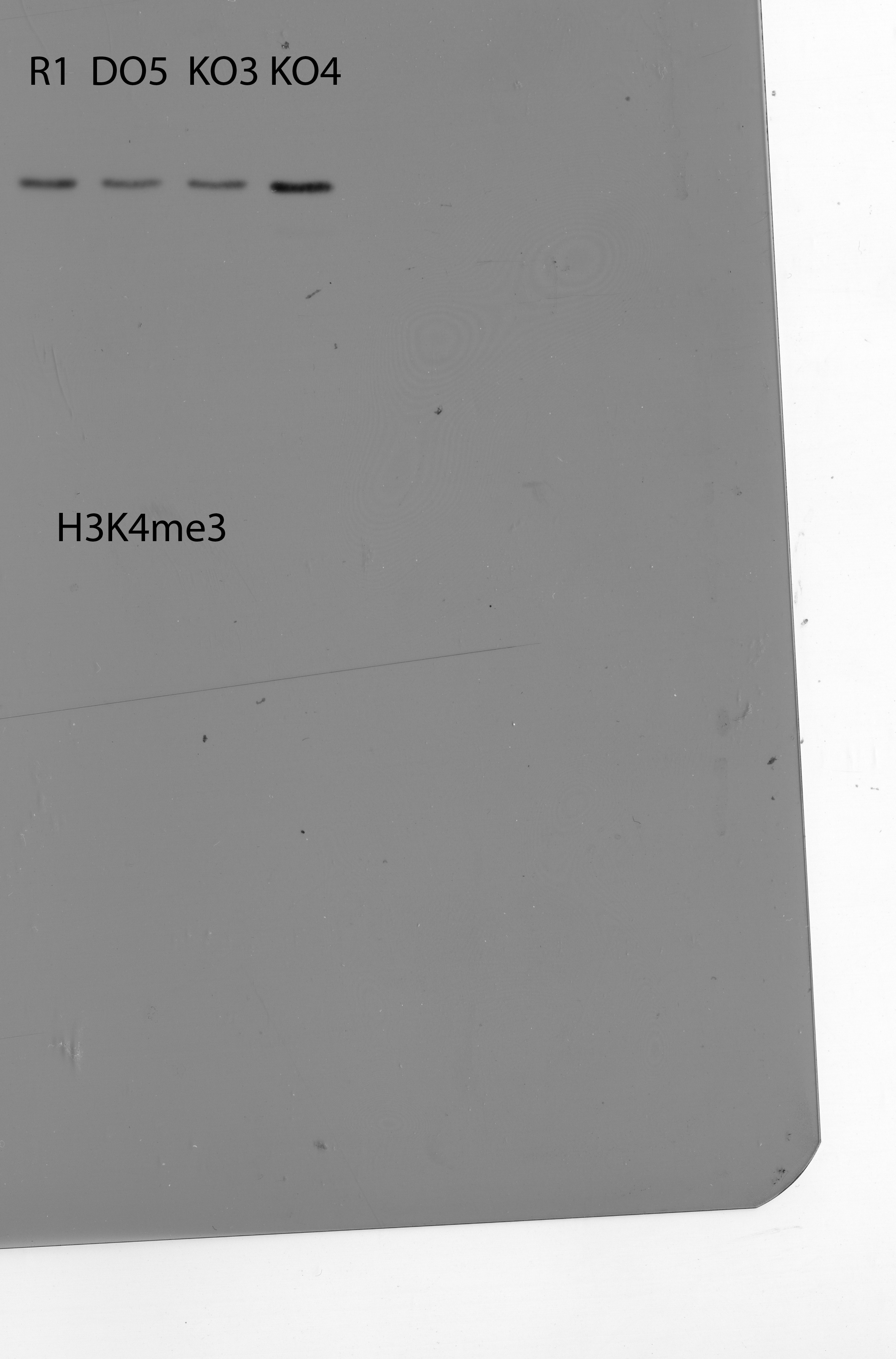

Supplement: S6 File — (ZIP) [file pone.0306360.s006.zip › File S6/Figure S2I right panel H3K4me3 Labelled.tif]

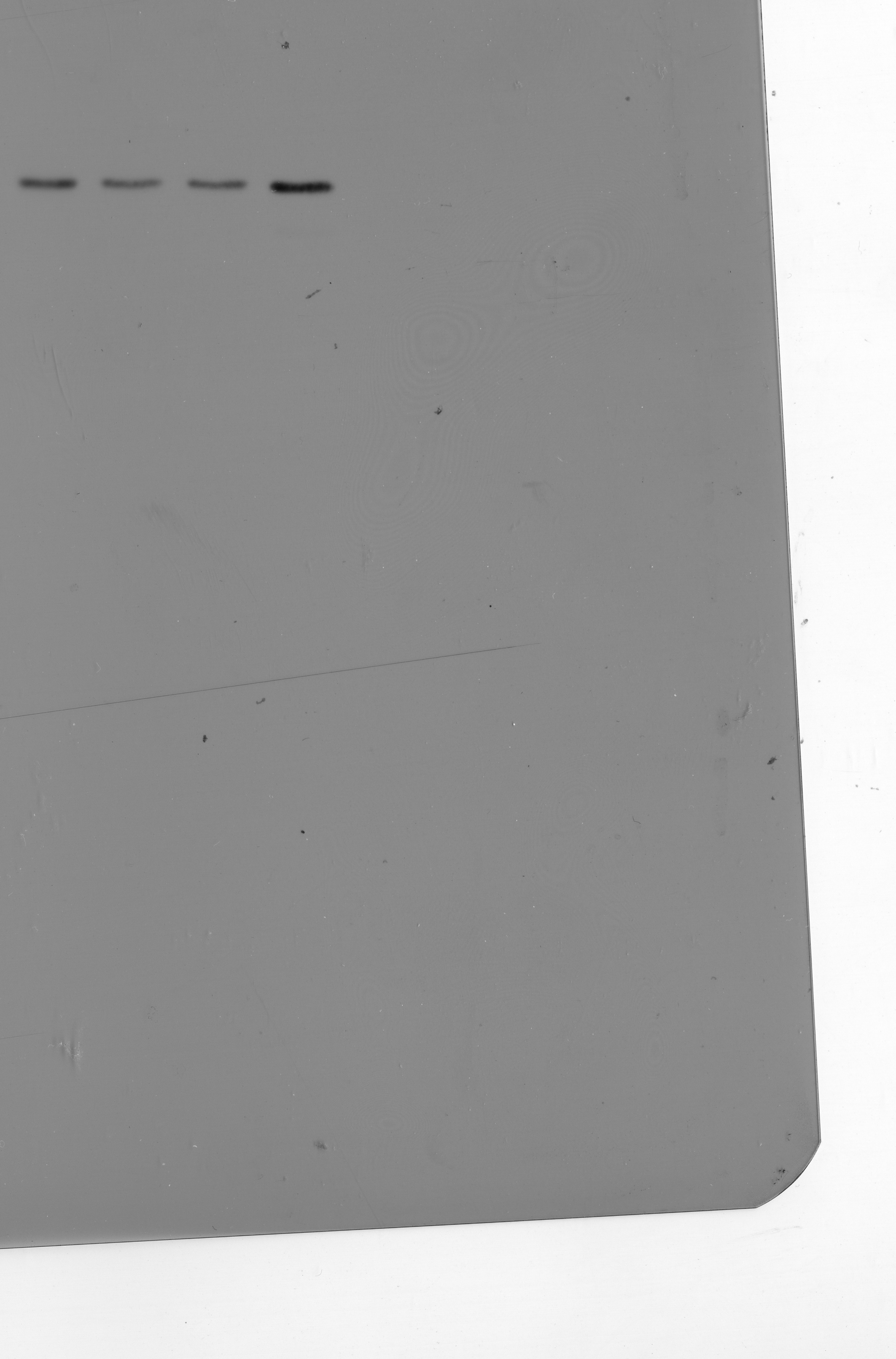

Supplement: S6 File — (ZIP) [file pone.0306360.s006.zip › File S6/Figure S2I right panel H3K4me3.tif]
