# Supplementary material for: Correction: Utx Is Required for Proper Induction of Ectoderm and Mesoderm during Differentiation of Embryonic Stem Cells
Source: PLoS One. 2024 Jun 27;19(6):e0306360. doi: 10.1371/journal.pone.0306360 (PMC11210840; doi:10.1371/journal.pone.0306360)

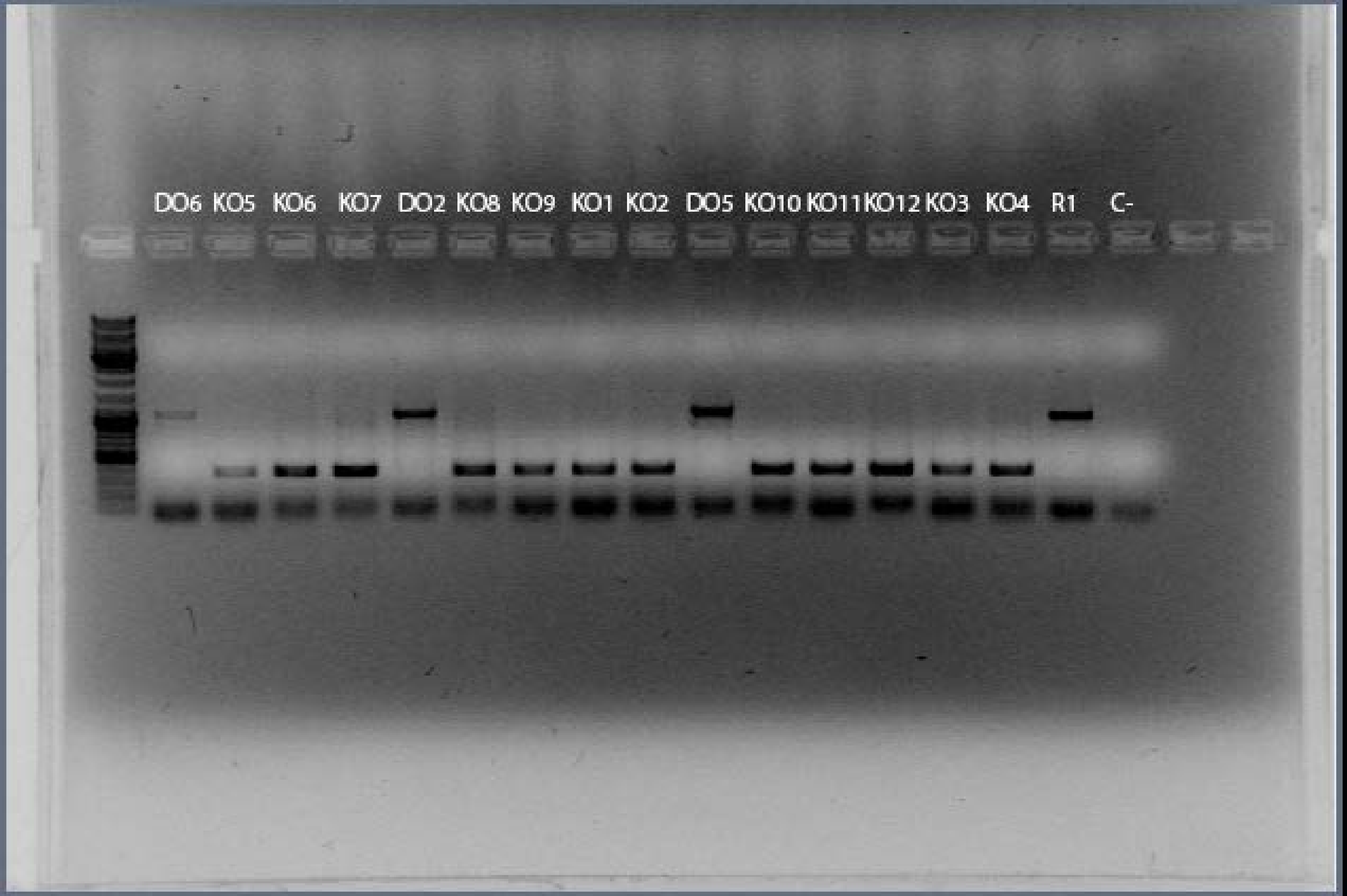
DO6 KO5 KO6 KO7 DO2 KO8 KO9 KO1 KO2 DO5 KO10 KO11 KO12 KO3 KO4 R1 C-

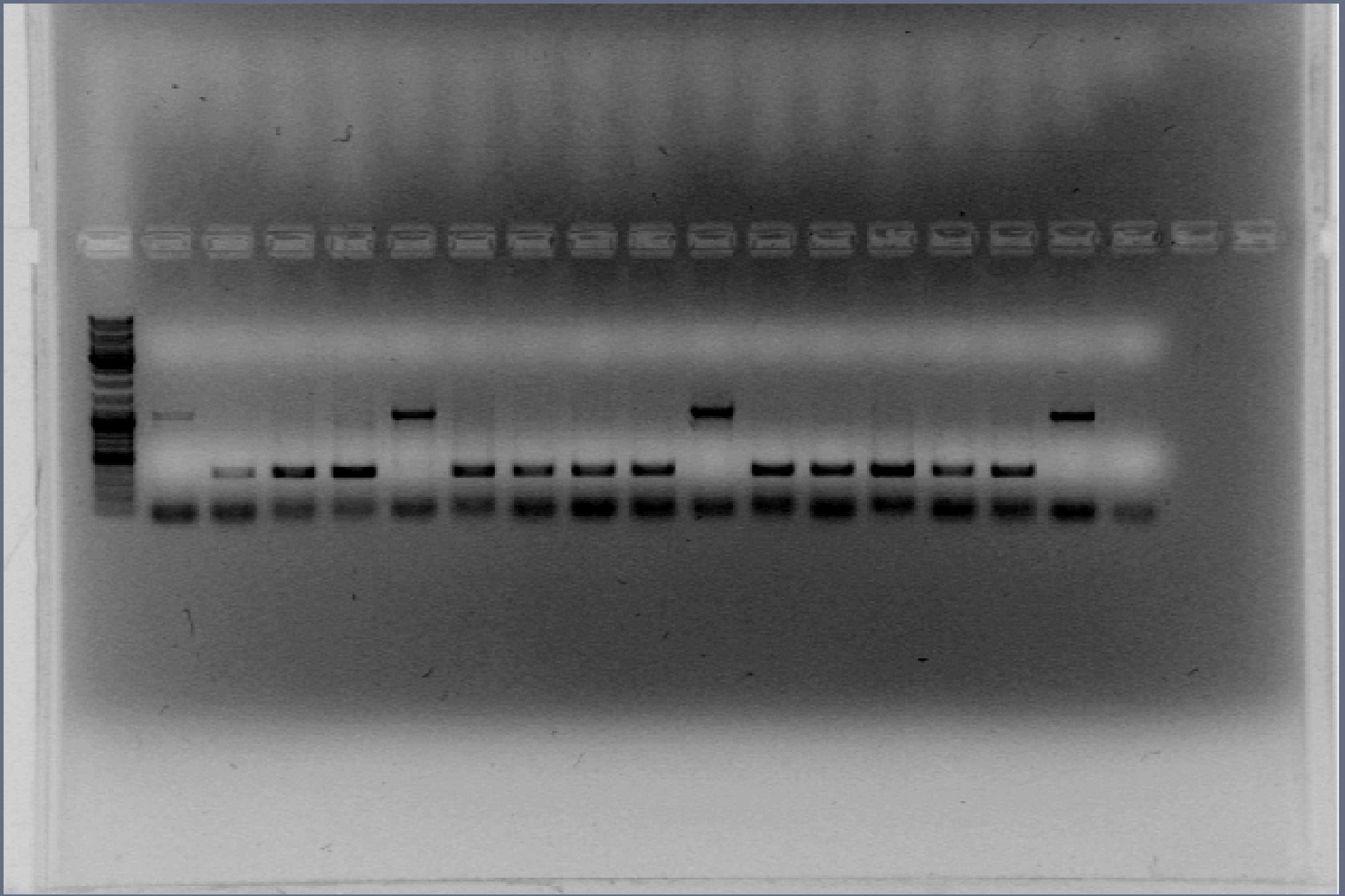

Supplement: S1 File — (ZIP) [file pone.0306360.s001.zip › S1 File/Figure 1.zip!/Figure 1/Figure 1D/Figure 1 D_UTX KO genotype PCR full gel.pdf]
