# Supplementary material for: Correction: Utx Is Required for Proper Induction of Ectoderm and Mesoderm during Differentiation of Embryonic Stem Cells
Source: PLoS One. 2024 Jun 27;19(6):e0306360. doi: 10.1371/journal.pone.0306360 (PMC11210840; doi:10.1371/journal.pone.0306360)

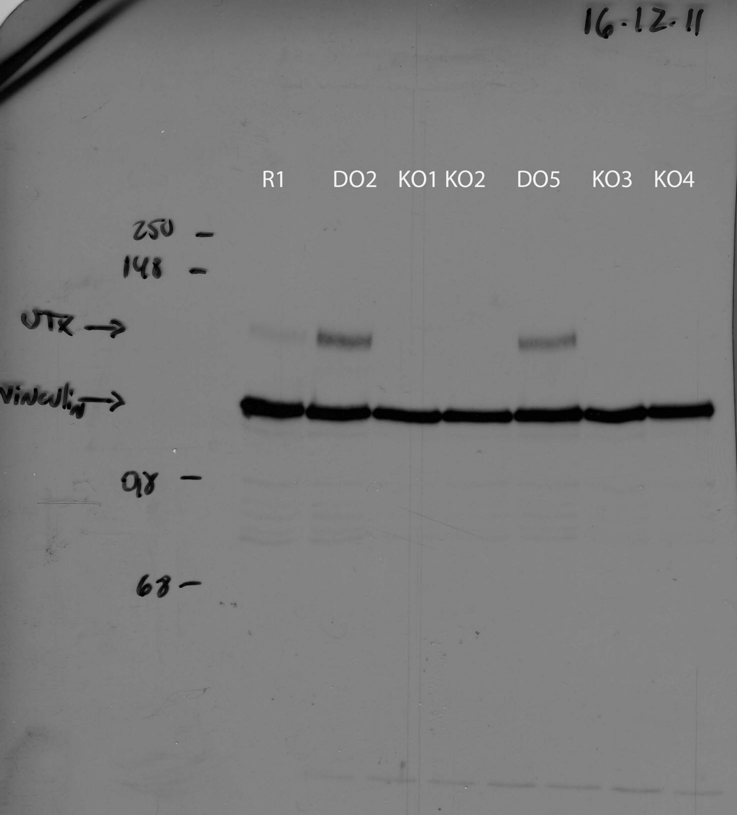
16.12.11
R1
DO2
KO1
KO2
DO5
KO3
KO4
250
148
UTX
Vinculin
98
68

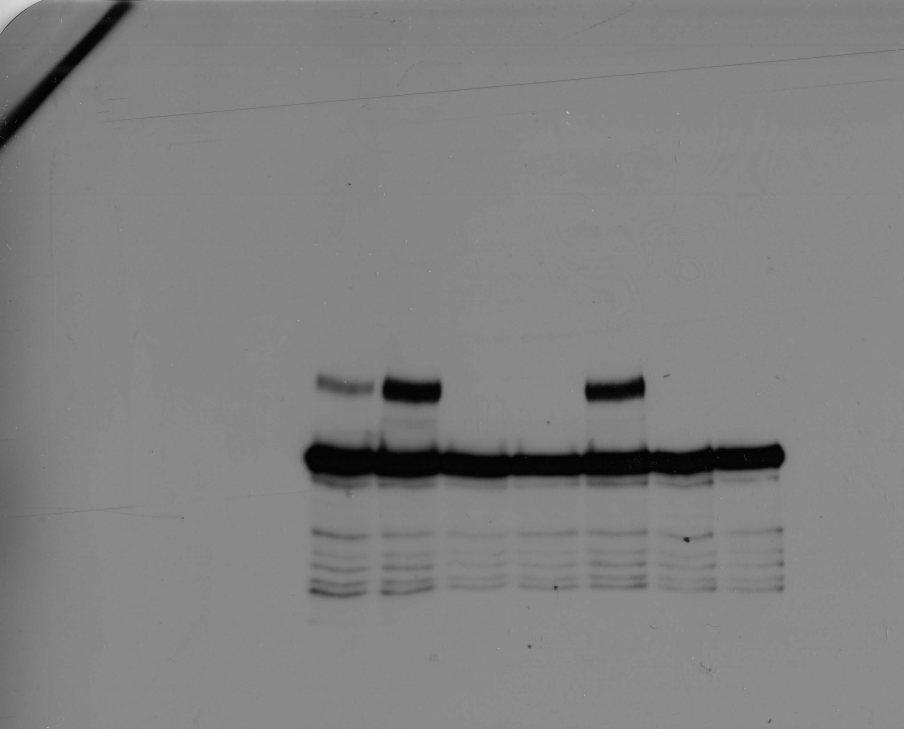

Supplement: S1 File — (ZIP) [file pone.0306360.s001.zip › S1 File/Figure 1.zip!/Figure 1/Figure 1F/Figure 1F And Figure S2B_Full gel_UTX and Vinculin.pdf]
