# Supplementary material for: Correction: Utx Is Required for Proper Induction of Ectoderm and Mesoderm during Differentiation of Embryonic Stem Cells
Source: PLoS One. 2024 Jun 27;19(6):e0306360. doi: 10.1371/journal.pone.0306360 (PMC11210840; doi:10.1371/journal.pone.0306360)

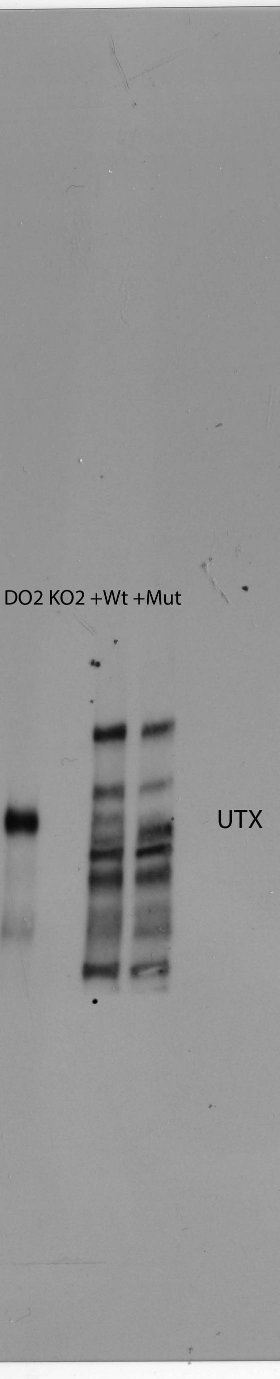
DO2 KO2 +Wt +Mut
UTX

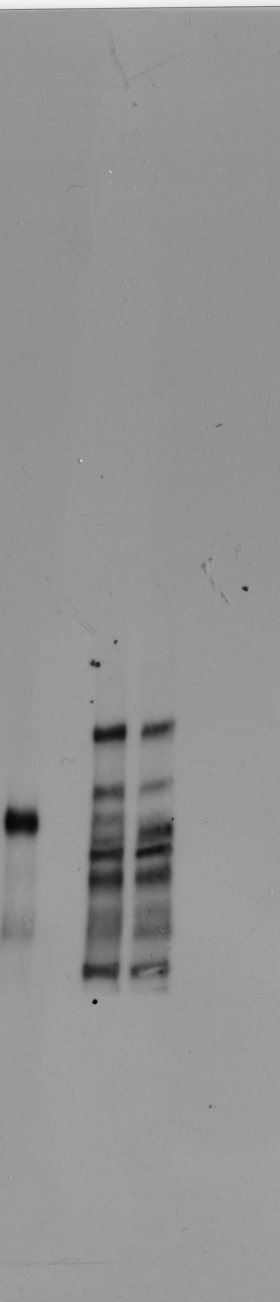

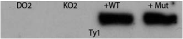
DO2
KO2
+WT
+ Mut
Ty1

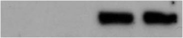

Supplement: S1 File — (ZIP) [file pone.0306360.s001.zip › S1 File/Figure 1.zip!/Figure 1/Figure 1H/Figure 1H UTX and Ty1.pdf]
