# Supplementary material for: Correction: Utx Is Required for Proper Induction of Ectoderm and Mesoderm during Differentiation of Embryonic Stem Cells
Source: PLoS One. 2024 Jun 27;19(6):e0306360. doi: 10.1371/journal.pone.0306360 (PMC11210840; doi:10.1371/journal.pone.0306360)

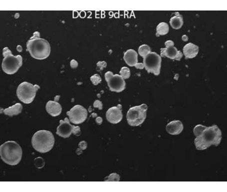
DO2 EB 9d-RA

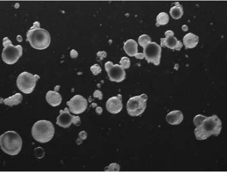

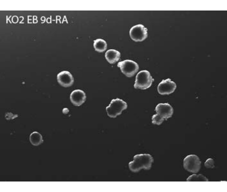
KO2 EB 9d-RA

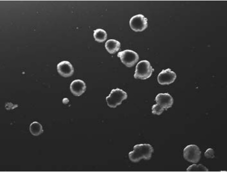

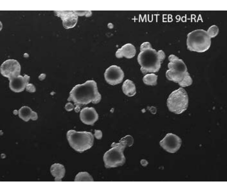
+MUT EB 9d-RA

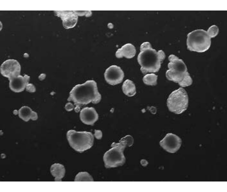

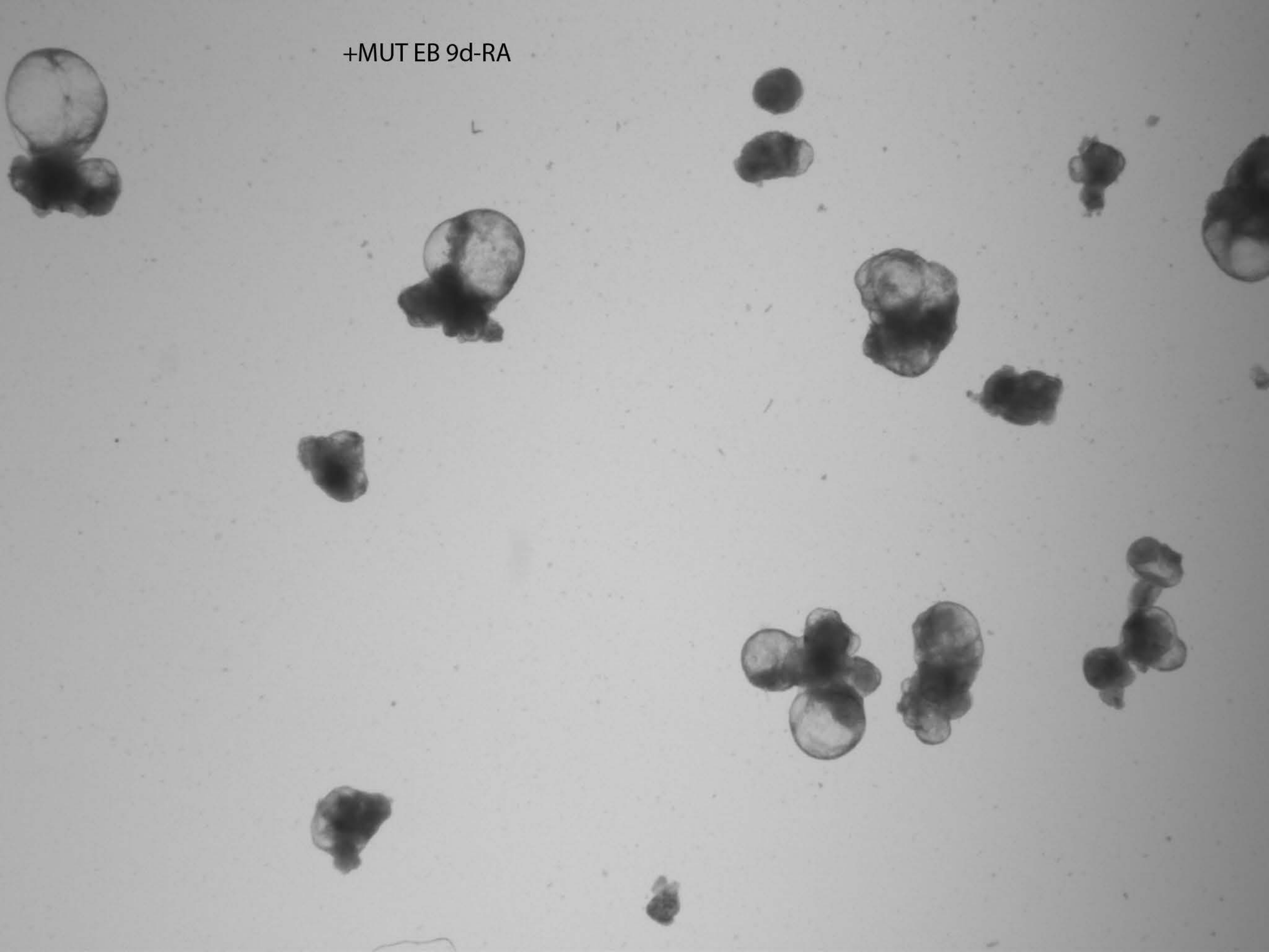
+MUT EB 9d-RA

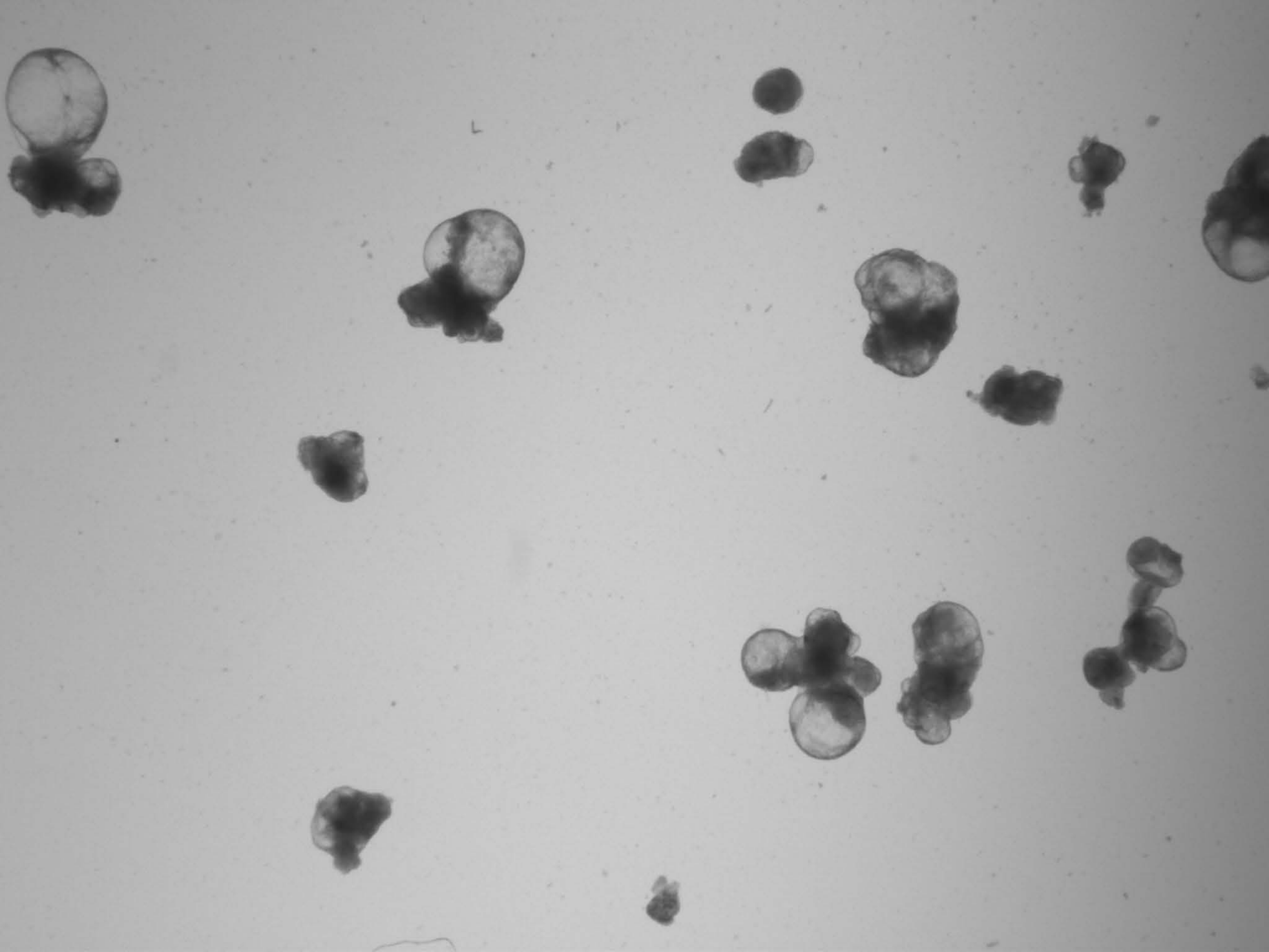

Supplement: S1 File — (ZIP) [file pone.0306360.s001.zip › S1 File/Figure 5.zip!/Figure 5/Figure 5A/Figure 5A EB 9d-RA.pdf]
