# Supplementary material for: Correction: Utx Is Required for Proper Induction of Ectoderm and Mesoderm during Differentiation of Embryonic Stem Cells
Source: PLoS One. 2024 Jun 27;19(6):e0306360. doi: 10.1371/journal.pone.0306360 (PMC11210840; doi:10.1371/journal.pone.0306360)

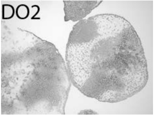
DO2

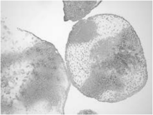

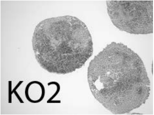
KO2

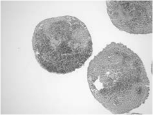

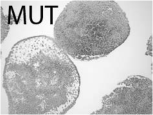
MUT

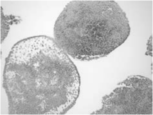

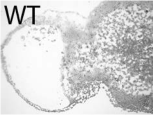
WT

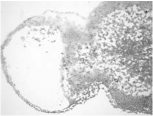

Supplement: S1 File — (ZIP) [file pone.0306360.s001.zip › S1 File/Figure 5.zip!/Figure 5/Figure 5B/Figure 5B H&E EB.pdf]
