# Supplementary material for: Correction: Utx Is Required for Proper Induction of Ectoderm and Mesoderm during Differentiation of Embryonic Stem Cells
Source: PLoS One. 2024 Jun 27;19(6):e0306360. doi: 10.1371/journal.pone.0306360 (PMC11210840; doi:10.1371/journal.pone.0306360)

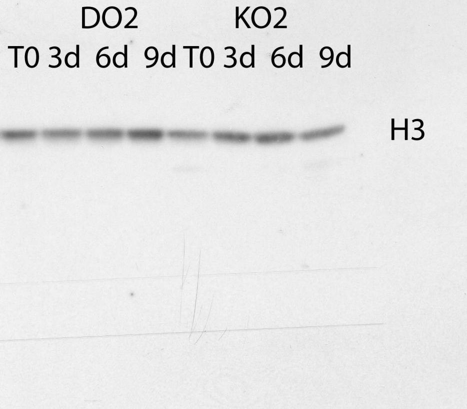
DO2
KO2
T0 3d 6d 9d T0 3d 6d 9d
H3

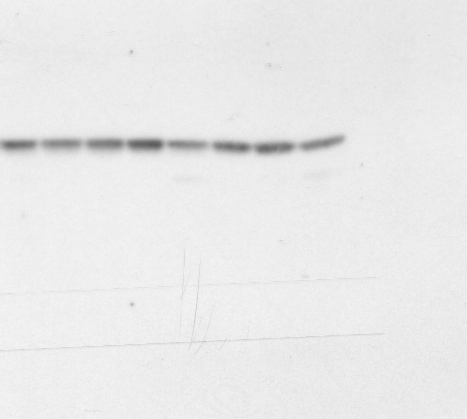

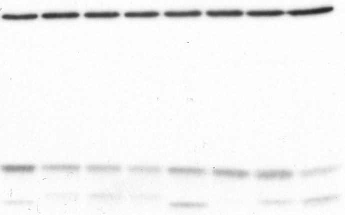

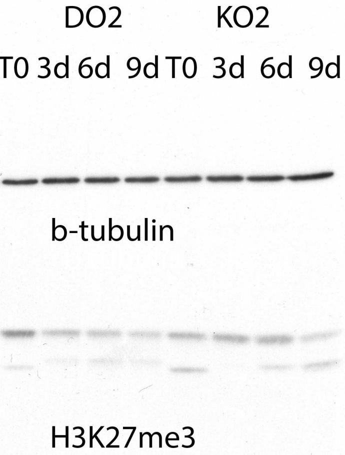
DO2
KO2
T0 3d 6d 9d T0 3d 6d 9d
b-tubulin
H3K27me3

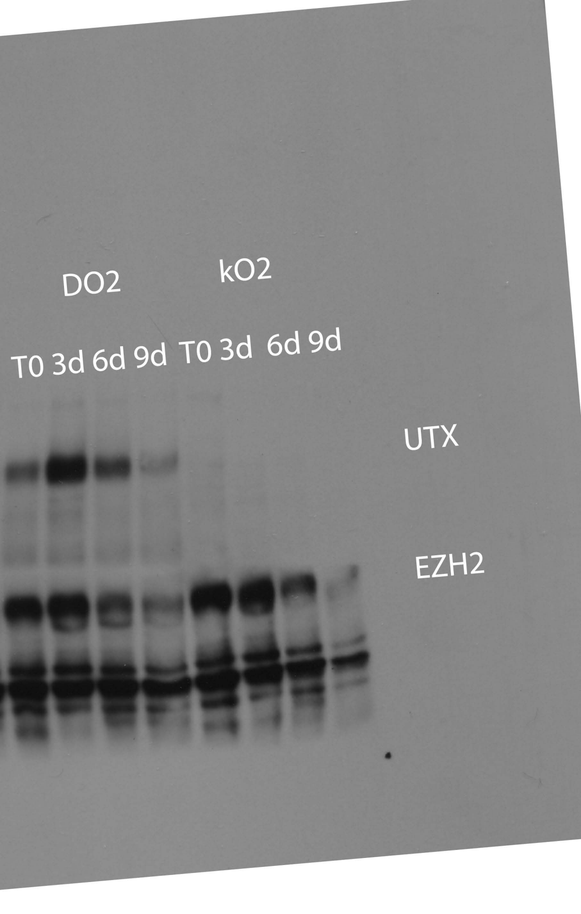
DO2
kO2
T0 3d 6d 9d T0 3d 6d 9d
UTX
EZH2

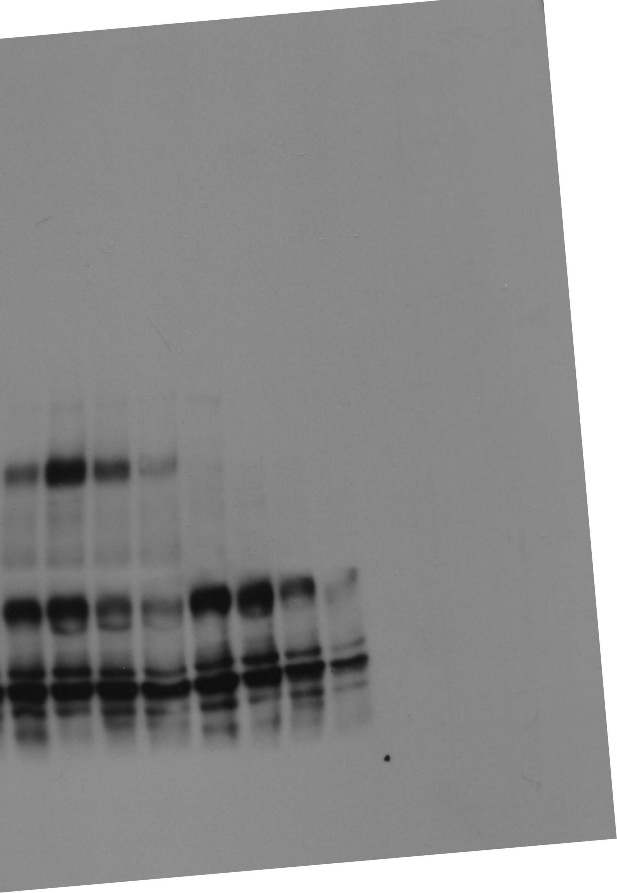

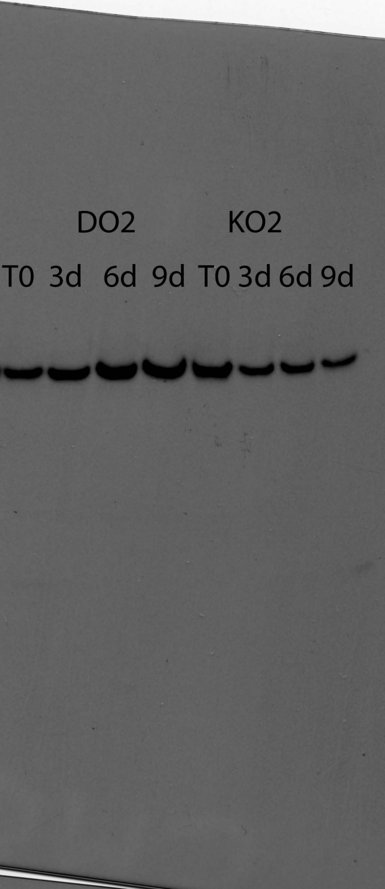
DO2
KO2
T0 3d 6d 9d T0 3d 6d 9d

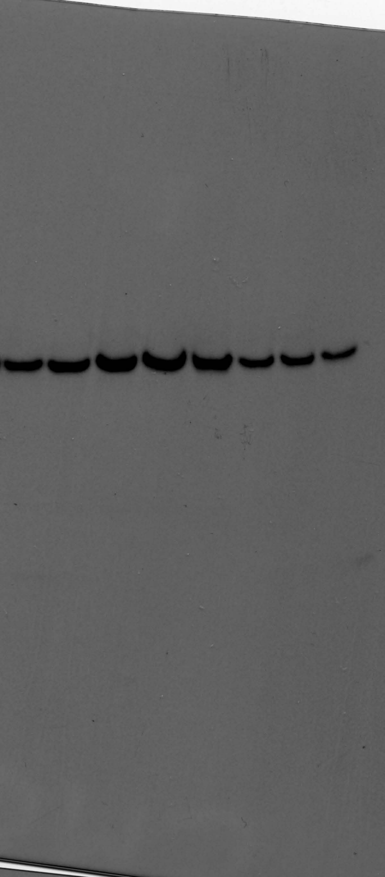

Supplement: S1 File — (ZIP) [file pone.0306360.s001.zip › S1 File/Figure 5.zip!/Figure 5/Figure 5E/Figure 5E_UT_EZH2_H3_H3K27_Vinculin_b tubulin.pdf]
