# Supplementary material for: Correction: Utx Is Required for Proper Induction of Ectoderm and Mesoderm during Differentiation of Embryonic Stem Cells
Source: PLoS One. 2024 Jun 27;19(6):e0306360. doi: 10.1371/journal.pone.0306360 (PMC11210840; doi:10.1371/journal.pone.0306360)
Supplement: S2 File — (ZIP) [file pone.0306360.s002.zip › S2 File/S2 File/Figure 7.zip!/Figure 7/Figure 7C/Figure 7C H3K27m3_H3_B tubulin.pdf]

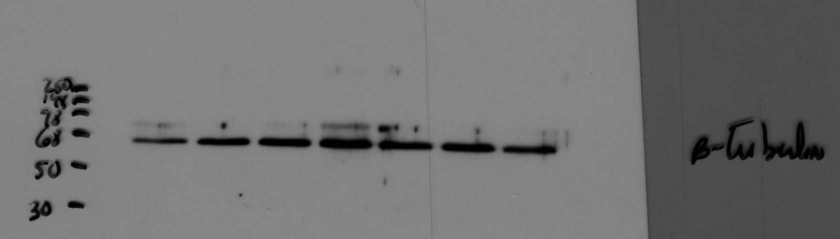
98
68
50
30
β-Tubulin

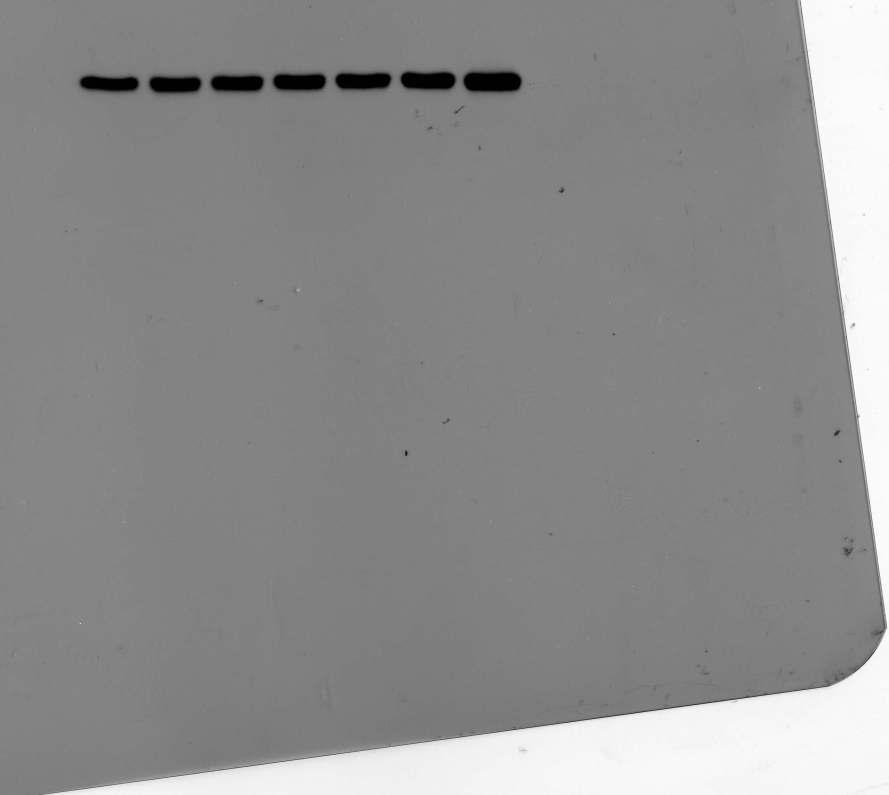

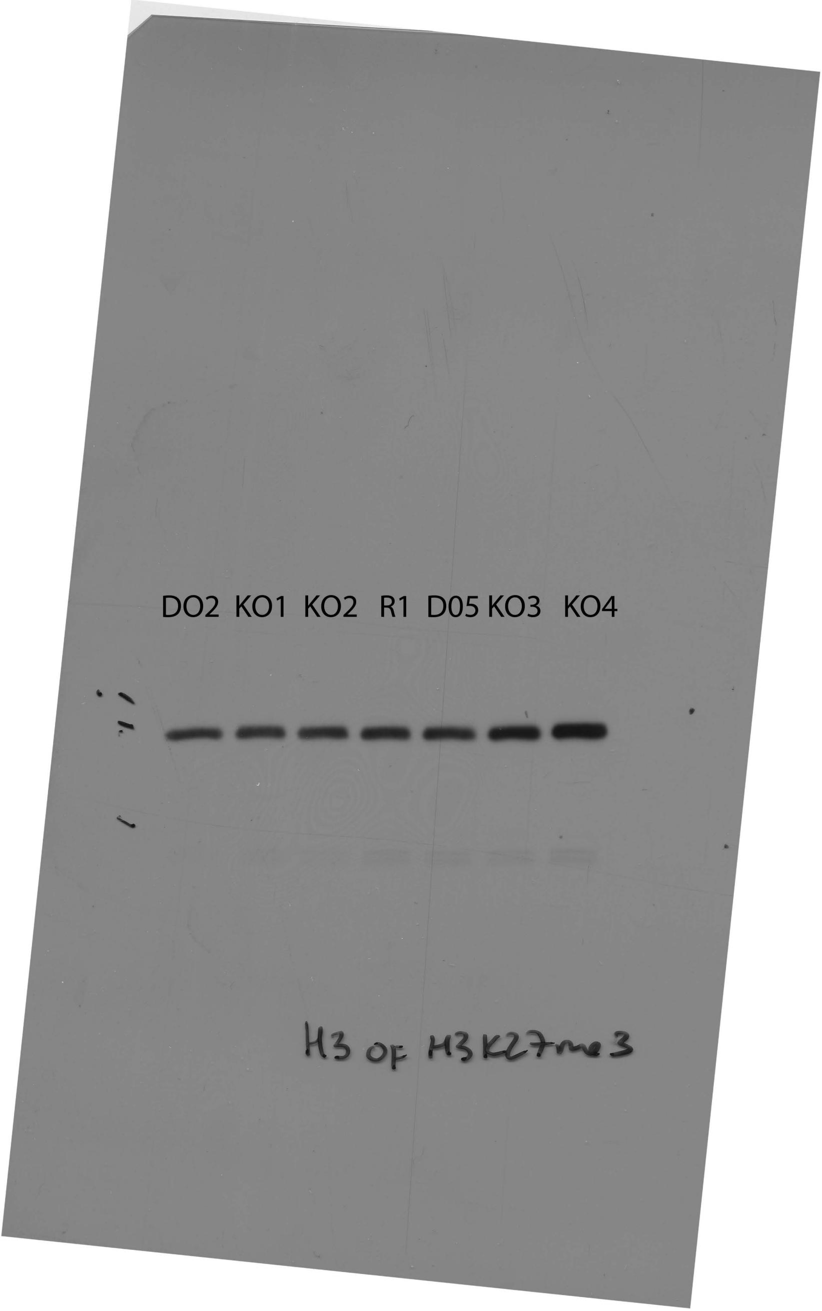
DO2 KO1 KO2 R1 D05 KO3 KO4
H3 of H3K27me3

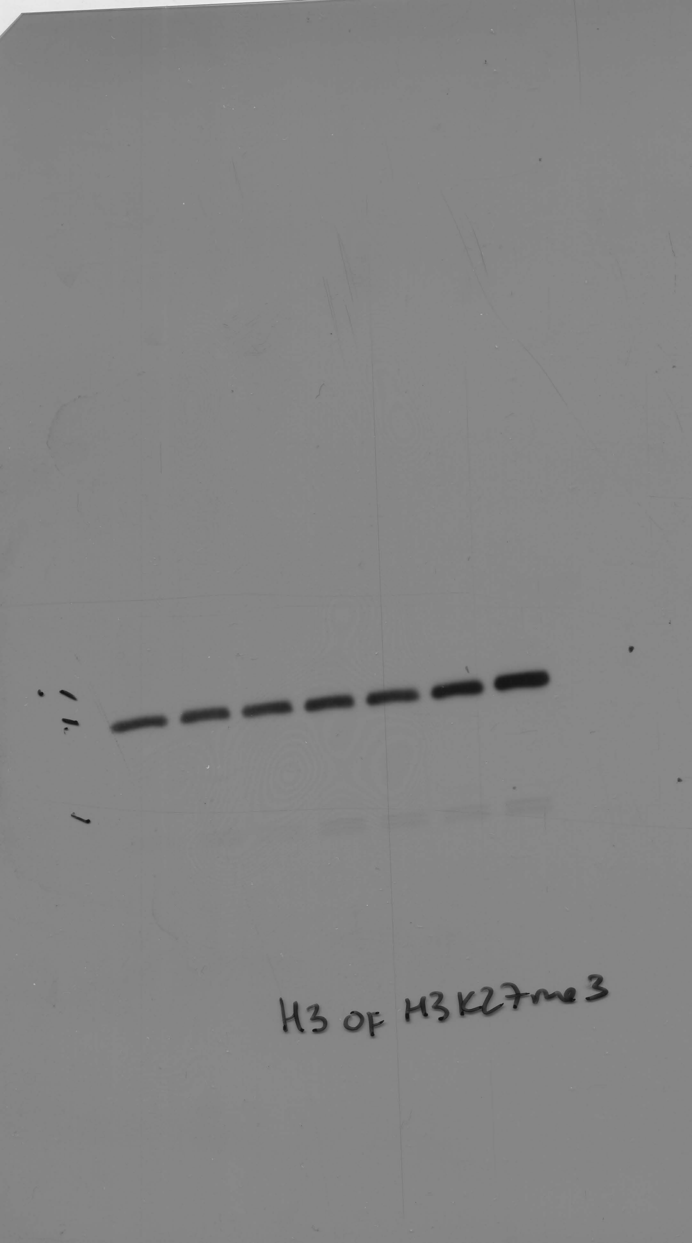
H3 of H3K27me3

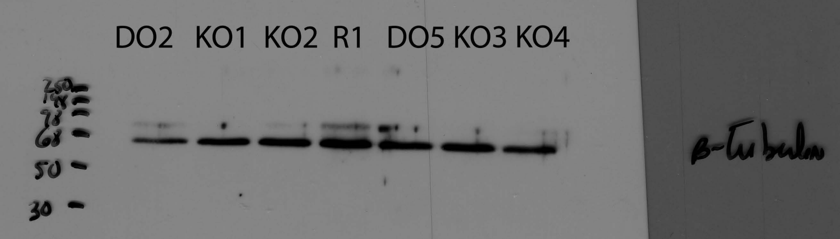
DO2
KO1
KO2
R1
DO5
KO3
KO4
250
98
68
50
30
β-Tubulin

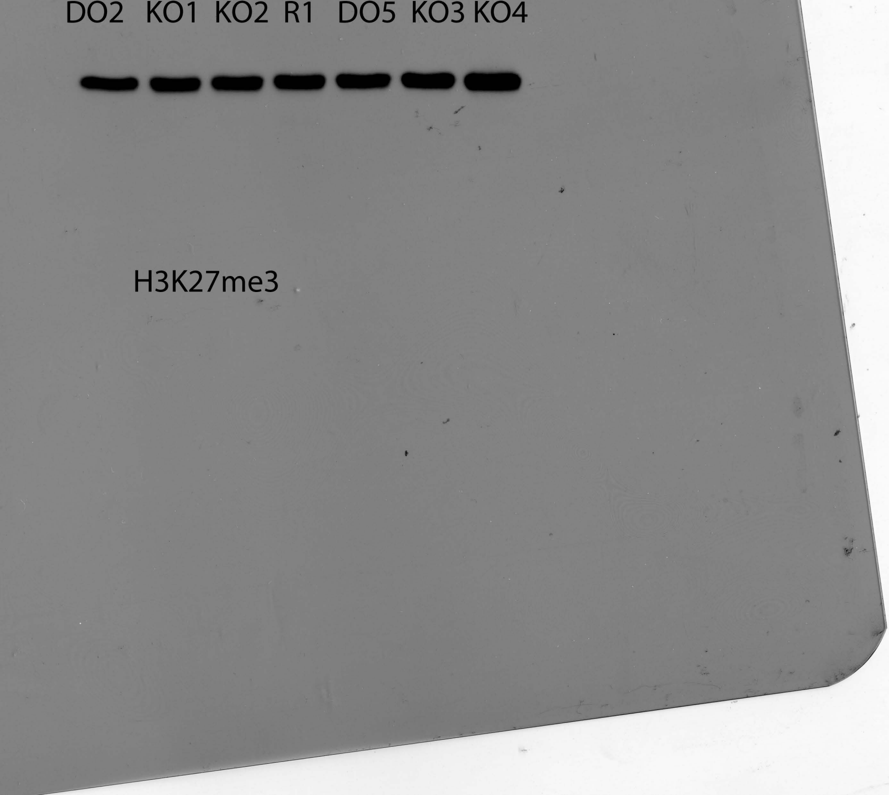
DO2 KO1 KO2 R1 DO5 KO3 KO4
H3K27me3
